# Electrophysiology-based screening identifies neuronal HtrA serine peptidase 2 (HTRA2) as a synaptic plasticity regulator participating in tauopathy

**DOI:** 10.1038/s41398-025-03227-4

**Published:** 2025-01-10

**Authors:** Naizhen Zheng, Kun Li, Jing Cao, Zijie Wang, Liang Zhang, Zihao Zhao, Jiawei He, Yong Wang, Xiang Zhu, Yiqing Chen, Jian Meng, Dongdong Zhao, Mengxi Niu, Hong Luo, Xian Zhang, Hao Sun, Yun-wu Zhang

**Affiliations:** Xiamen Key Laboratory of Brain Center, The First Affiliated Hospital of Xiamen University, and Fujian Provincial Key Laboratory of Neurodegenerative Disease and Aging Research, Institute of Neuroscience, School of Medicine, Xiamen University, Xiamen, Fujian 361102 China

**Keywords:** Hippocampus, Molecular neuroscience

## Abstract

Long-term potentiation (LTP) and long-term depression (LTD) are widely used to study synaptic plasticity. However, whether proteins regulating LTP and LTD are altered in cognitive disorders and contribute to disease onset remains to be determined. Herein, we induced LTP and LTD in the hippocampal CA3-CA1 Schaffer collateral pathway, respectively, and then performed proteomic analysis of the CA1 region. We identified 20 differentially expressed proteins (DEPs) shared by the LTP and the LTD processes. Among them, we found that HtrA serine peptidase 2 (HTRA2) was mainly expressed in neurons and that HTRA2 levels were increased in both the LTP and the LTD processes in C57BL/6 mice. HTRA2 downregulation impaired synapses and reduced ATP production in cultured primary neurons. Furthermore, adeno-associated virus (AAV)-mediated HTRA2 downregulation in the hippocampus impaired synaptic plasticity and cognitive function in C57BL/6 mice. Moreover, we found that HTRA2 expression decreased in the brains of Alzheimer’s disease patients, frontotemporal lobar degeneration with ubiquitin inclusions patients, and tauopathy model mice. Finally, we showed that lentivirus-mediated HTRA2 overexpression in the hippocampus rescued PP2B reduction, alleviated tau hyperphosphorylation, and partially attenuated synaptic plasticity and cognitive deficits in the PS19 tauopathy model mice. Our study not only indicates that HTRA2 in neurons plays an important role in regulating synaptic plasticity under both physiological and pathological conditions, but also provides a novel, electrophysiology-based strategy to identify proteins regulating synaptic plasticity systematically.

## Introduction

Long-term potentiation (LTP) and long-term depression (LTD) are phenomena in which high-frequency stimulation (HFS) and low-frequency stimulation (LFS) via activation of presynaptic neurons lead to rapid and long-lasting synaptic strength and depression in the postsynaptic terminal, respectively [1]. LTP and LTD are cellular mechanisms underlying synaptic plasticity [1]. LTP and LTD impairments have been found in cognitive disorders such as Alzheimer’s disease (AD) and related tauopathies [2, 3], which are a series of neurodegenerative disorders that share the same pathology of tau protein aggregation in the brain [4].

LTP and LTD formation requires the activation of NMDA and AMPA receptors [5, 6], as well as the participation of other proteins for appropriate regulation [7]. Therefore, identifying new proteins regulating LTP and LTD will enhance our understanding of the molecular mechanisms underlying synaptic plasticity and the pathogenesis of cognitive disorders. Because protein expression in postsynaptic neurons changes in response to HFS-induced LTP and LFS-induced LTD [8], we hypothesize that although the change of most proteins may be an accompanying phenomenon, some changed proteins may participate in the induction and maintenance of LTP and LTD, and therefore be necessary for synaptic plasticity. Moreover, if these proteins are dysregulated in cognitive disorders, they may contribute to disease pathogenesis.

Based on this hypothesis, we induced LTP and LTD in the hippocampal CA3-CA1 Schaffer collateral pathway and then studied protein changes in the CA1 region. We identified a series of proteins possibly involved in synaptic plasticity. Among them, we found that the protein expression of HtrA serine peptidase 2 (HTRA2) increased upon both LTP and LTD inductions. Furthermore, we showed that HTRA2 downregulation in neurons impaired synaptic function in vitro and synaptic plasticity and cognitive function in vivo. Moreover, we found that HTRA2 expression decreased in the brains of tauopathy patients and tauopathy model mice, whereas HTRA2 overexpression in neurons partially attenuated memory and synaptic plasticity deficits in the PS19 tauopathy model mice.

## Materials and methods

### Human samples

Human brain tissue lysates were from National Developmental and Functional Human Brain Bank, Chinese Academy of Medical Sciences, National Health and Disease Human Brain Tissue Resource Center, and Brain Bank and Neurodegenerative Disease Research Center in China. The primary neuropathologic diagnosis, age at death, and gender of the human subjects are listed in Table S1.

### Animals and ethics statement

PS19 mice (IMSR_JAX:008169, B6;C3 background) were initially obtained from the Jackson Laboratory and maintained by backcrossing with C57BL/6 mice for over ten generations. C57BL/6 WT and PS19 male mice were used in this study. Animal procedures were performed in accordance with the guidelines of the National Institutional Animal Health Guide for the Care and Used of Laboratory Animals and approved by the Animal Ethics Committee of Xiamen University (XMULAC20190074 and XMULAC20210188).

### Electrophysiological recordings

LTP and LTD were performed as previously described [9, 10]. Briefly, mice were anesthetized with isoflurane. Mouse brain was rapidly removed and put into an ice-cold solution (64 mM NaCl, 2.5 mM KCl, 10 mM glucose, 1.25 mM NaH_2_PO_4_, 10 mM MgSO_4_, 26 mM NaHCO_3_, 120 mM sucrose, and 0.5 mM CaCl_2_). Brain slices were sectioned (400 μm) on a Leica VT1200S vibratome, incubated at 32°C for 1 h, and recovered at room temperature for at least 1 h before recording in artificial cerebrospinal fluid (126 mM NaCl, 3.5 mM KCl, 1.25 mM NaH_2_PO_4_, 1.3 mM MgSO_4_, 2.5 mM CaCl_2_, 26 mM NaHCO_3_, and 10 mM glucose) with 95% O_2_/5% CO_2_. A stimulating electrode was placed in the hippocampal CA3. Field excitatory postsynaptic potentials (fEPSPs) in the hippocampal CA1 were recorded using a micropipette. LTP was induced by two trains of high-frequency stimulation (HFS, 100 Hz, 1 s) with an interval of 30 s. LTD was induced by 900 trains of low-frequency stimulation (LFS, 1 Hz, 1 s) with an interval of 1 s. Data were acquired with a Multiclamp 700B patch-clamp amplifier (Molecular Devices) and analyzed using pClamp software (Molecular Devices, v10.6).

For whole-cell recording, cultured mouse primary neurons were infected with GFP-expressing viruses on days in vitro (DIV) 2. On DIV15-DIV16, neurons expressing GFP were subjected to electrophysiological recordings of the spontaneous EPSCs (sEPSCs) and the spontaneous IPSCs (sIPSCs), following protocols described previously [11]. sEPSCs and sIPSCs were recorded at holding potentials of −70 mV and 0 mV, respectively. The frequency and amplitude of sEPSC and sIPSC events were analyzed using Clampfit 10.4 software (Molecular Devices) and Mini Analysis Program (Synaptosoft).

### Hippocampal CA1 tissue acquisition

After LTP or LTD recordings, the hippocampal CA1 region on brain slices was rapidly collected by a capillary-based vacuum-assisted cell and tissue acquisition system, UnipicK^TM^ (NeuroInDx). The CA1 region was recognized using an ECLIPSE Ts2 inverse microscope and captured by a disposable capillary unit with a 40 μm inner diameter tip.

### Proteomic analysis

Collected hippocampal CA1 tissues were subjected to microproteomics performed by Beijing Genomics Institute (BGI, Shenzhen, China). Briefly, the high-resolution mass spectrometer Orbitrap Fusion Lumos Tribrid (Thermo Fisher Scientific) was used to detect the data dependent acquisition (DDA) mode of library samples and microsamples. The analysis was performed using the integrated MaxQuant’s Andromeda engine. DEPs were identified by quantitative analysis based on peak intensity, peak area, and retention time of liquid chromatography related to MS1. Screening was performed based on the multiple of difference > 1.2 and *P* value < 0.05 as the criteria.

The latest KEGG pathway gene annotations were obtained from the KEGG database (https://www.genome.jp/kegg/) and served as the background. The differential genes were mapped to the background set. Both Gene Ontology and KEGG enrichment analyses were performed using the R software package clusterProfiler (v3.14.3), with a *P* value < 0.05 considered to be statistically significant.

### Primary cell cultures

Primary neurons, astrocytes and microglia were cultured following previously reported protocols [12, 13]. Additional details are provided in Supplemental Methods.

### Stereotactic injection of viruses

To downregulate HTRA2 in the hippocampus, WT male mice at different ages were injected bilaterally with AAV-shNC-EGFP or AAV-shHTRA2-EGFP at the following coordinates (anterior posterior, −2.0 mm; medial lateral, ±1.6 mm; dorsal ventral, −1.8 mm).

For HTRA2 overexpression in the hippocampus, WT and PS19 mice at 6 months of age were injected bilaterally with Lentivirus-EGFP-NC or Lentivirus-EGFP-HTRA2-Flag at the above-mentioned coordinates. All viruses used in this study were packaged by Obio Technology Corp, Ltd.

### Immunoblotting

Samples were lysed in RIPA lysis buffer (25 mM Tris-HCl [pH7.6], 150 mM NaCl, 0.1% SDS, 1% sodium deoxycholate, and 1% Non-idet P-40) supplemented with protease inhibitors. 20 microgram protein lysates were subjected to SDS-PAGE separation. Proteins were immunoblotted with primary antibodies overnight at 4 °C and with secondary antibodies for 1 h at room temperature, and then detected with enhanced chemiluminescent reagent.

Antibodies used included: anti-NeuN (94403S, 1:1000), anti-β-actin (8457S, 1:10000), anti-GluN1 (5704S, 1:1000), anti-PSD93 (19046S, 1:1000), anti-p35/25 (2680, 1:1000), anti-PSD95 (3450S, 1:1000), anti-tau pS181 (12885S, 1:1000), and anti-SYN1 (5297S, 1:1000) from Cell Signaling Technology; anti-GFAP (16825-1-AP, 1:1000), anti-HTRA2 (15775-1-AP, 1:1000), anti-GluN2A (19953-1-AP, 1:1000), anti-GluN2B (19954-1-AP, 1:1000), anti-GSK3β (51065-1-AP, 1:1000), anti-GluA2 (11994-1-AP, 1:1000), anti-pT216-GSK3β (51065-1-AP, 1:1000), and anti-flag (66008-4-lg, 1:1000) from Proteintech; anti-PP2B-Aα (sc-17808, 1:1000) and anti-CDK5 (sc-173, 1:1000) from Santa Cruz Biotechnology; anti-PP1α (43-8100, 1:1000) and HRP-conjugated secondary antibodies (31460/31430, 1:5000) from Thermo Fisher Scientific; anti-Iba1 (016-20001, 1:500) from Wako; anti-tau pS202/T205 (MN1020, 1:500), anti-tau pS396 (44752 G, 1:1000), and anti-Total tau (Tau5, AHB0042, 1:1000) from Invitrogen; anti-Synaptophysin (S5768, 1:2000) from Sigma-Aldrich; anti-GluA1 (MAB2263, 1:1000) from Millipore; anti-GAPDH (AB0037, 1:5000) from Abways; and anti-PP2A-α (A6702, 1:1000) from ABclonal. Protein band intensities were quantified by ImageJ (NIH).

### Immunostaining

Mice were anesthetized and perfused with 1×PBS. Mouse brains were dissected and fixed in 4% paraformaldehyde for 16 h at 4 °C. Brain sections (15 μm thick) were treated with Citrate Antigen Retrieval Solution for 10 min and blocked in blocking buffer (3% BSA and 0.2% Triton X-100) at room temperature for 1 h.

For immunostaining of HTRA2 in response to HFS and LFS, Brain slices were first sectioned at 400 μm for HFS and LFS treatments. Slices were then quickly put into 4% paraformaldehyde for fixation overnight at 4 °C, cryoprotected in PBS containing 25% sucrose for 2 d and in PBS containing 30% sucrose for another 4 d, and frozen in OCT at −80 °C. Frozen slices were further sectioned into 18-μm slices using Leica microtome.

Sections were incubated with primary antibodies overnight at 4 °C and with fluorescence-conjugated secondary antibodies at room temperature for 1 h. The nuclei were stained with DAPI for 1 h. Antibodies used included: anti-NeuN (Cell Signaling Technology, 94403S, 1:200), anti-Iba1 (Wako, 016-20001, 1:200), anti-GFAP (Proteintech, 16825-1-AP, 1:200), anti-HTRA2 (Proteintech, 15775-1-AP, 1:200), and secondary antibodies (Thermo Fisher Scientific, A-11005 or A-11012, 1:400). Images were captured with the A1R (Nikon) or FV1000MPE-B (Olympus) confocal microscope.

### ATP assay

Mouse primary neurons were infected with AVV-shHTRA2 or a scrambled negative control shRNA (NC) for 12 d. Cells were measured for ATP levels using an ATP assay kit (S0026, Beyotime), following the manufacturer’s instructions.

### Behavioral tests

Treated mice were subjected to various behavioral studies, including Y-maze, T-maze, novel object recognition, and Morris water maze tests, following previously reported protocols [9, 11]. Additional details are provided in Supplemental Methods.

### Statistical analysis

All behavioral analyses were carried out in a double-blind manner. Other experiments were studied in a single-blind manner. Statistical analysis was performed using GraphPad Prism 8. Sample sizes were determined based on the assumption of a normal distribution and similar variability between experimental groups. No animals or samples were excluded from or randomized in the analyses. Parametric or nonparametric tests were performed based on the acceptance of normality. For comparisons between two groups, a normality test was conducted first. Samples with a normal distribution were compared using Student’s *t* test, whereas samples with no normal distribution were compared using Mann-Whitney test. For comparisons in more than two groups, differences were analyzed using one-way ANOVA or two-way ANOVA. Samples with a normal distribution were compared using one-way ANOVA with Tukey’s post hoc analysis, whereas samples with no normal distribution were compared using one-way ANOVA with Dunn’s post hoc analysis. All data represent mean ± SEM. *P* < 0.05 was considered statistically significant. Detailed statistical method for each comparison was described in figure legends.

## Results

### LTP and LTD-based screening identifies proteins involved in synaptic function

**Fig. 1:**
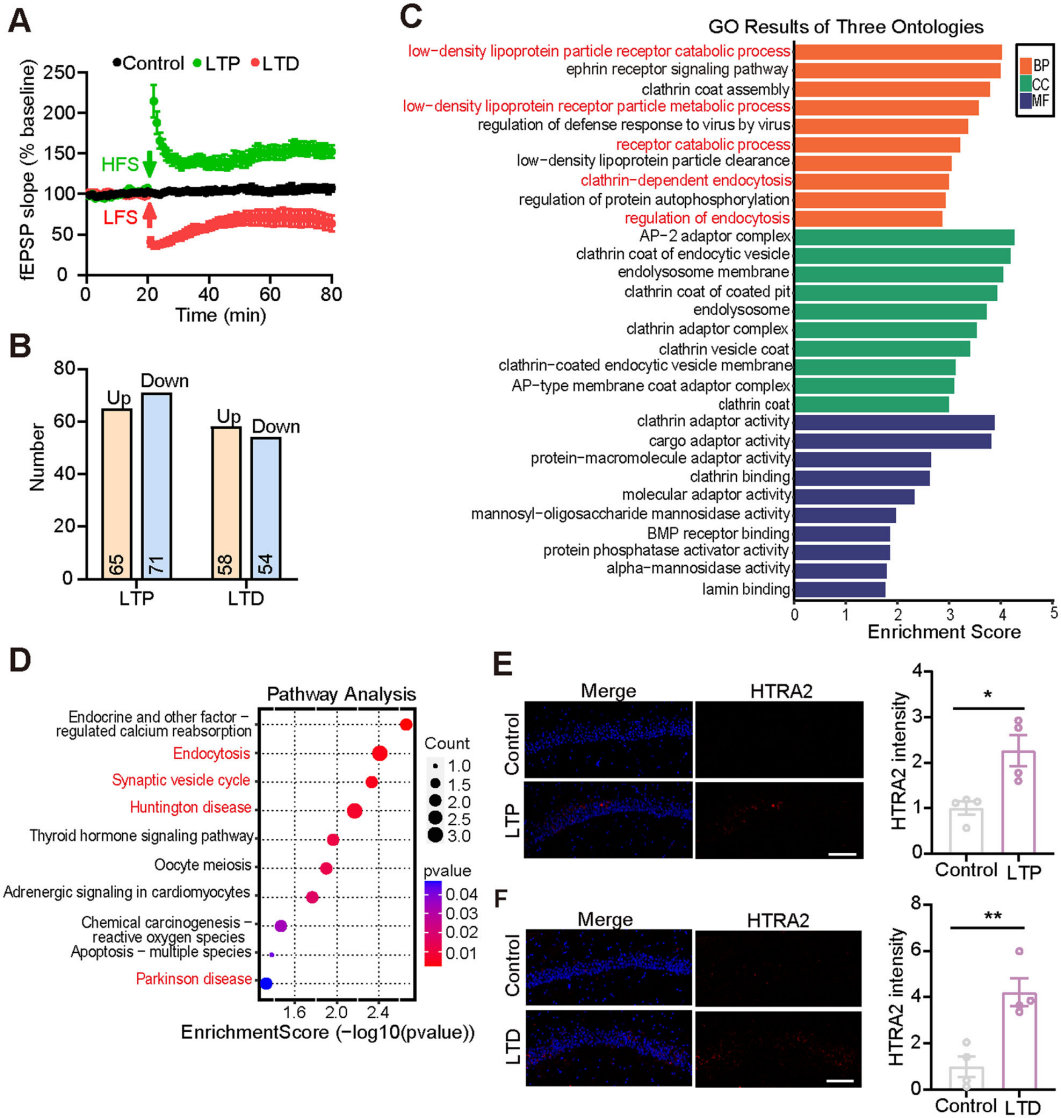
LTP- and LTD-based screening to identify proteins involved in synaptic plasticity. **A** Hippocampal slices from WT mice at 8 months of age were stabilized for 20 min. The hippocampal CA3 region was subjected to high-frequency stimulation (HFS) to induce LTP (in green), low-frequency stimulation (LFS) to induce LTD (in red), or no stimulation as controls (in black). The fEPSPs in the hippocampal CA1 region were then recorded for another 60 min. *n* = 8 slices from 8 mice for each group. **B** The numbers of upregulated (Up) and downregulated (Down) proteins in CA1 induced by LTP or LTD. GO (**C**) and KEGG (**D**) enrichment analysis of the 20 DEPs shared by both the LTP and the LTD processes. Representative images of HTRA2 (red) and DAPI (blue) in CA1 of 8-month-old WT mice after LTP (**E**) and LTD (**F**) inductions (*n* = 4 slices from 4 mice). Scale bars, 100 μm. HTRA2 immunostaining intensity was quantified for comparison. A significant difference was determined using the Mann-Whitney test (**E**), and an unpaired *t*-test (**F**). Data represent means ± SEM. *p < 0.05; **p < 0.01.

We first studied LTP and LTD in the hippocampal CA3-CA1 Schaffer collateral pathway in WT mice at 8-month-old. LTP is routinely used to study learning and memory in adult mice. Although some previous reports suggested that LTD was difficult to induce using LFS in adult mice [14, 15], successful examples still exist [16, 17]. We were able to induce both LTP and LTD in WT mice at 8-month-old (Fig. 1A).

Next, we collected the hippocampal CA1 region after LTP and LTD inductions for proteomic analysis. 136 differentially expressed proteins (DEPs, with 65 upregulated and 71 downregulated) were identified in samples subjected to LTP (Fig. 1B; Table S2), and 112 DEPs (with 58 upregulated and 54 downregulated) were identified in samples subjected to LTD (Fig. 1B; Table S3), when compared to the controls without stimulation.

For DEPs identified in LTP, Gene Ontology (GO) enrichment analysis showed that many were involved in biological processes related to metabolic processes of receptors and mitochondrial respiration (Fig. S1A). KEGG enrichment analysis suggested that many of them participated in neurodegenerative diseases such as amyotrophic lateral sclerosis, AD, and Huntington’s disease (HD) (Fig. S1B). For DEPs identified in LTD, GO enrichment analysis showed that many were involved in biological processes related to protein localization and transport (Fig. S1C), and KEGG enrichment analysis found that many participated in pathways such as dopaminergic synapse, oxidative phosphorylation, and LTD (Fig. S1D). These results suggest that LTP and LTD have different mechanisms, consistent with their different induction methods and electrophysiological patterns. Nevertheless, LTP and LTD share some common molecular pathways, such as the activation of NMDA and AMPA receptors [5, 6]. Therefore, to screen proteins mediating both LTP and LTD, we intersected DEPs in LTP with those in LTD and acquired 20 shared DEPs (Table S4). Among the 20 DEPs, five were upregulated in both LTP and LTD, and the other 15 were downregulated also in both (Table S4). GO enrichment analysis of the 20 DEPs revealed that many were involved in biological processes related to the metabolic processes of receptors and endocytosis (Fig. 1C). KEGG enrichment analysis suggested that many of them participated in pathways such as endocytosis, synaptic vesicle cycle, and neurodegenerative diseases such as HD and Parkinson’s disease (PD) (Fig. 1D).

Among the 20 DEPs identified, HTRA2 has been linked to AD, one of the most common cognitive disorders [18, 19]. However, so far there is no report on whether HTRA2 participates in synaptic plasticity to our knowledge. Therefore, for proof-of-concept purpose, we selected HTRA2 for further functional studies. We confirmed that HTRA2 levels were increased in the hippocampal CA1 region after LTP and LTD inductions (Fig. 1E, F).

### HTRA2 downregulation impairs synaptic transmission in mouse primary neurons

**Fig. 2:**
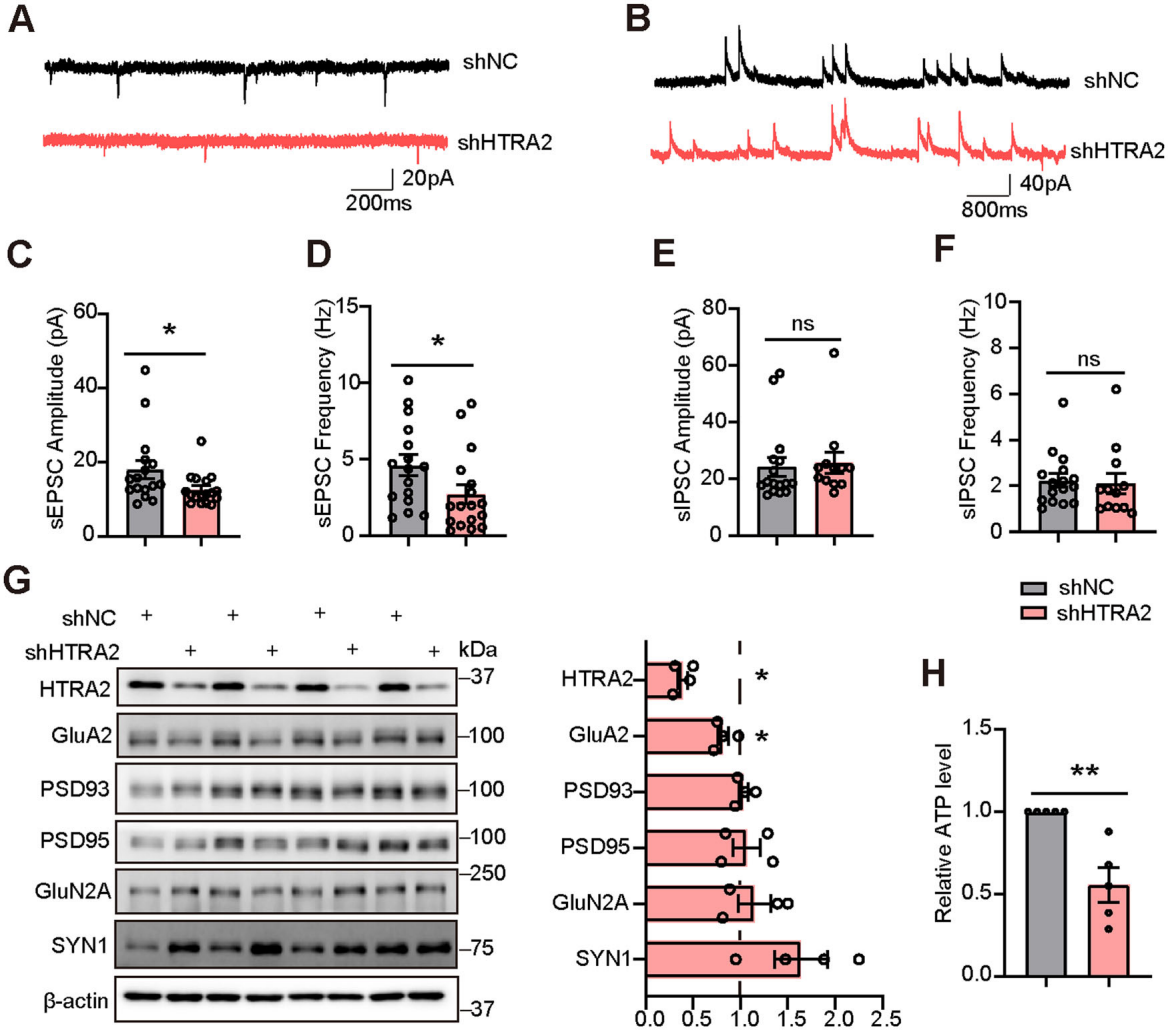
Knockdown of HTRA2 impairs synaptic transmission and ATP production in mouse primary neurons. Representative sEPSC (**A**) and sIPSC (**B**) traces in neurons infected with shNC and shHTRA2 viruses. Histogram plots of amplitudes (**C**, **E**) and frequencies (**D**, **F**) of sEPSCs (**C**, **D**) and sIPSCs (**E**, **F**) (*n* = 12–17 neurons from 3 independent experiments). **G** Representative Western blotting and quantification comparison of indicated proteins in neurons infected with shNC and shHTRA2 viruses (*n* = 4 per group, controls were set to one arbitrary unit and indicated by a dashed line). **H** Comparison of ATP levels in neurons infected with shNC and shHTRA2 viruses (*n* = 5 per group). A significant difference was determined using the Mann-Whitney test. Data represent means ± SEM. *p < 0.05; **p < 0.01; ns, not significant.

We found that HTRA2 was mainly expressed in cultured primary neurons and astrocytes, with less abundance in microglia (Fig. S2A, B). To study the function of HTRA2 in neurons, we first infected WT primary neurons with AAV2/9 carrying various *HTRA2* shRNAs (AAV-shHTRA2#1, AAV-shHTRA2#2, and AAV-shHTRA2#3) and found that all of them reduced HTRA2 protein levels (Fig. S3A, B). We then used the most effective one (AAV-shHTRA2#1, referred to as AAV-shHTRA2 hereafter) and its control (AAV-shNC) for in vitro and *vivo* studies. When cultured WT primary neurons were infected with AAV-shHTRA2 and AAV-shNC, we found that HTRA2 knockdown significantly decreased the frequency and amplitude of sEPSCs (Fig. 2A, C, D), without affecting those of sIPSCs (Fig. 2B, E, F). AMPA and NMDA receptors are known to regulate synaptic function [5, 6]. We also assayed AMPA and NMDA receptor subunits and found that HTRA2 knockdown reduced protein levels of GluA2 in cultured primary neurons (Fig. 2G). Since HTRA2 plays a role in maintaining mitochondrial homeostasis [20], we studied and found that HTRA2 knockdown also reduced adenosine triphosphate (ATP) production in cultured primary neurons (Fig. 2H). These results indicate that HTRA2 downregulation impairs mitochondrial function in neurons, which may be a mechanism underlying the synaptic function regulation by HTRA2.

### HTRA2 downregulation in hippocampal neurons impairs synaptic and cognitive function in WT mice

**Fig. 3:**
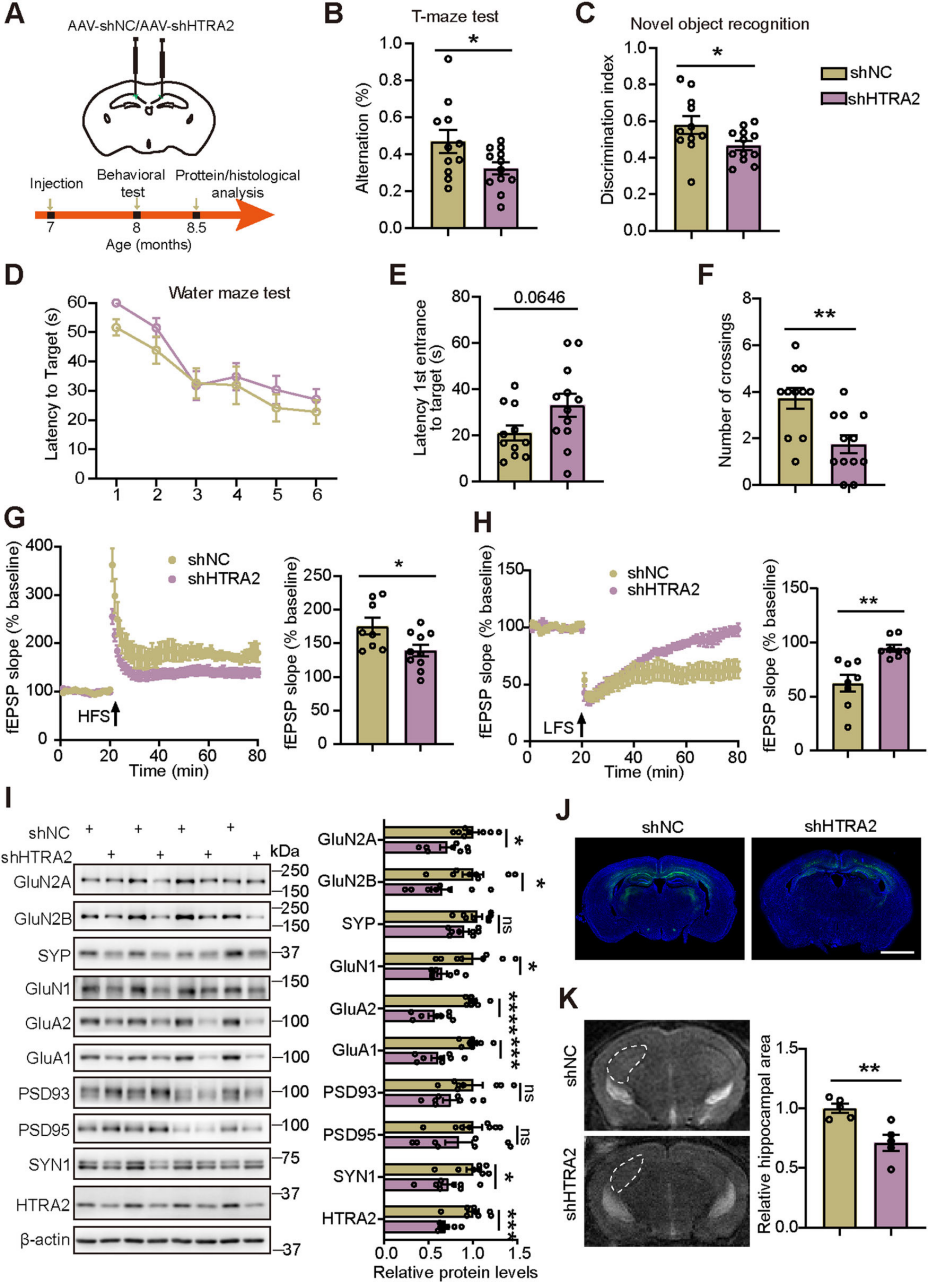
Knockdown of HTRA2 in hippocampal neurons of 7-month-old WT mice impairs synaptic and cognitive function. **A** The scheme of AAV2/9 constructs expressing *HTRA2* shRNA (AAV-shHTRA2) and scrambled negative control shRNA (AAV-shNC) injection in the hippocampal CA1 region and experimental procedures. **B** In the T maze test, the spontaneous alteration percentage of mice was studied for comparison. **C** In the novel object recognition test, the discrimination index of novel versus familiar objects was studied for comparison. In the Morris water maze test, the escape latency during a six-day training was recorded for comparison for every day (**D**). On the seventh day, the time spent for the first entry into the target quadrant (**E**) and the number of platform crossings (**F**) were recorded for comparison. AAV-shNC=11 mice, and AAV-shHTRA2 = 12 mice for all behavioral tests. LTP (**G**) and LTD (**H**) recordings in the hippocampal CA1 region and quantification of the mean fEPSP slope in the last 10 min of recordings (*n* = 8–10 slices from 5 mice per group). **I** Representative western blotting and quantification comparison of indicated proteins (shNC=8 mice, and shHTRA2 = 8 mice). **J** Representative images of the hippocampal region of WT mice injected with AAV-shNC or AAV-shHTRA2. Scale bar; 2000 μm. **K** Representative images of brain MRI scan and quantification comparison of the hippocampal region size (shNC = 5 mice, and shHTRA2 = 5 mice). Hippocampal regions were indicated with white dashed lines. A significant difference was determined using an unpaired *t*-test. Data represent means ± SEM. *p < 0.05; **p < 0.01; ***p < 0.001; ****p < 0.0001; ns, not significant.

Next, we delivered AAV-shHTRA2 and AAV-shNC into the hippocampal CA1 region of 2- (Fig. S4A) or 7-month-old WT mice (Fig. 3A). EGFP signal was found to colocalize with NeuN-positive cells predominantly (Fig. S3C) but not with GFAP-positive cells (Fig. S3D) or Iba1-positive cells (Fig. S3E) in the hippocampal CA1 region, suggesting that *HTRA2* shRNA specifically knocked down HTRA2 in neurons. We also confirmed that HTRA2 protein levels were significantly downregulated in the hippocampal region by immunoblotting (Figs. 3I, S4J).

For mice injected with AAV at 7-month-old, we studied their behaviors one month later. We found that AAV-shHTRA2 mice showed no significant change in total travel distance compared to controls in the open field test (Fig S5A), suggesting that HTRA2 downregulation does not affect locomotor activity. In both T and Y maze tests, AAV-shHTRA2 mice had significantly reduced spontaneous alteration percentages compared to controls (Figs. 3B, S5B). In the novel object recognition test, AAV-shHTRA2 mice showed a significantly reduced discrimination index (Fig. 3C). In the Morris water maze test, although AAV-shHTRA2 mice showed no differences in the escape latency during a six-day training and in the latency of the first entry into the target quadrant during the testing phase on the 7th day (Fig. 3D, E), they had decreased platform crossing numbers during the testing phase on the 7th day (Fig. 3F). We also examined LTP and LTD in the CA3-CA1 pathway and found that both were impaired in AAV-shHTR2 mice compared to controls (Fig. 3G, H). Moreover, we assayed synaptic proteins and found that protein levels of GluN2A, GluN2B, GluN1, GluA2, GluA1, and SYN1 in the hippocampal region were markedly reduced upon HTRA2 downregulation (Fig. 3I). Finally, immunofluorescence (Fig. 3J) and magnetic resonance imaging (MRI) (Fig. 3K) analysis revealed that HTRA2 downregulation resulted in hippocampal atrophy.

We also found consistent results in mice injected with AAV at 2-month-old and studied two months later. Mice injected with AAV-shHTRA2 had no change in total travel distance in the open field test (Fig. S4B). However, they showed significantly reduced spontaneous alteration percentage in the Y maze test (Fig. S4C) and reduced discrimination index in the novel object recognition test (Fig. S4D). In the Morris water maze test, mice injected with AAV-shHTRA2 exhibited significantly increased escape latency during a six-day training, and increased latency of the first entry into the target quadrant and decreased platform crossing numbers during the testing phase on the 7th day (Fig S4E-G). Impaired LTP and LTD (Fig. S4H, I), reduced protein levels of GluN2A, GluN1, GluA2, PSD93, and PSD95 in the hippocampal region, and hippocampal atrophy (Fig. S4J-L) were also found in mice injected with AAV-shHTRA2 compared to controls.

Together, these results indicate that HTRA2 downregulation in the hippocampal region impairs synaptic and cognitive function in WT mice.

### HTRA2 expression is decreased in the brains of AD and FTLD-U patients and different tauopathy model mice

**Fig. 4:**
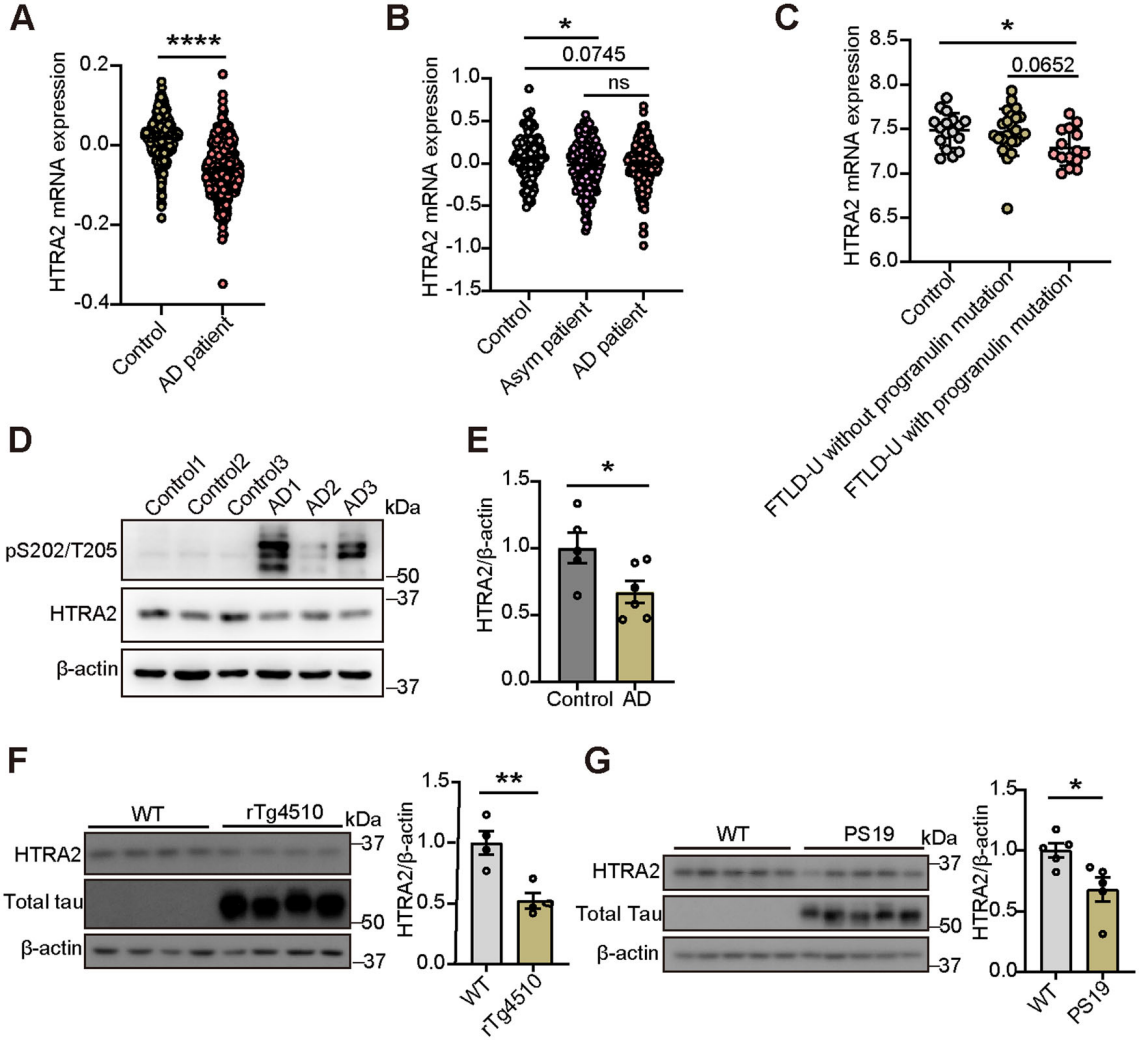
HTRA2 expression is decreased in the brain of tauopathy patients and tauopathy model mice. **A** Comparison of *HTRA2* mRNA expression in AD patients (*n* = 387) and controls (*n* = 303). Data from NCBI’s Gene Expression Omnibus (Accession number GEO: GSE44772). **B** Comparison of *HTRA2* mRNA expression in the dorsolateral prefrontal cortex of AD patients (*n* = 202), asymptomatic (Asym) AD patients (*n* = 204), and controls (*n* = 125) from the RNA-seq data from ref. [21]. **C** Comparison of *HTRA2* mRNA expression in FLTD-U patients with progranulin mutation patients (*n* = 15), FLTD-U patients without progranulin mutation patients (*n* = 24), and controls (*n* = 17). Data from NCBI’s Gene Expression Omnibus (Accession number GEO: GSE13162). **D**, **E** Representative Western blotting and quantification of HTRA2 protein levels in postmortem AD brains and controls (control: *n* = 5, and AD: *n* = 6). **F**, **G** Representative western blotting and quantification of HTRA2 protein levels in 7-month-old rTg4510 (*n* = 4 mice per group) and 10-month-old PS19 mouse hippocampal lysates (*n* = 5 mice per group). A significant difference was determined using the Mann-Whitney test (**A**), one-way ANOVA with Dunn’s post hoc analysis (**B**), one-way ANOVA with Tukey’s post hoc analysis (**C**), and an unpaired *t*-test (**E**, **F**, **G**). Data represent means ± SEM. *p < 0.05; **p < 0.01; ****p < 0.0001; ns, not significant.

We further explored whether HTRA2 expression was altered in AD and related tauopathies. In one study comparing gene expression of AD and control brain tissues (Accession number GEO: GSE44772), we noticed that *HTRA2* mRNA expression significantly decreased in AD (Fig. 4A). In another study comparing gene expression in brain tissues of controls, asymptomatic AD, and AD [21], we also found that *HTRA2* mRNA expression significantly decreased in both asymptomatic AD and AD (Fig. 4B). Furthermore, in a study comparing gene expression of brain tissues from controls, FTLD-U patients with progranulin mutations, and FTLD-U patients without progranulin mutations patients (Accession number GEO: GSE13162), we found that *HTRA2* mRNA expression significantly decreased in FTLD-U patients with progranulin mutations when compared to controls (Fig. 4C). We also studied limited postmortem brain tissues of AD patients and controls and found that HTRA2 protein levels decreased in AD samples (Fig. 4D, E). Moreover, we found that HTRA2 protein levels decreased in the hippocampal tissue samples of rTG4510 and PS19 mice, two animal models of tauopathy (Fig. 4F, G). However, HTRA2 protein levels were unaltered in the hippocampus and cortex of 5×FAD mice that exhibit AD-related β-amyloid (Aβ) pathology (Fig. S6A, B). These results indicate a link between HTRA2 alteration and tauopathy.

### HTRA2 overexpression ameliorates neurological dysfunction in PS19 mice

We also delivered lentiviruses expressing HTRA2 or EGFP (as controls) into the hippocampal CA1 region of WT and PS19 mice at six months of age (Fig. S7A). We found that EGFP expression predominantly colocalized with NeuN-positive cells but not with GFAP-positive cells or Iba1-positive cells in the hippocampal CA1 region (Fig. S7B), suggesting that exogenous HTRA2 was predominantly expressed in neurons.

**Fig. 5:**
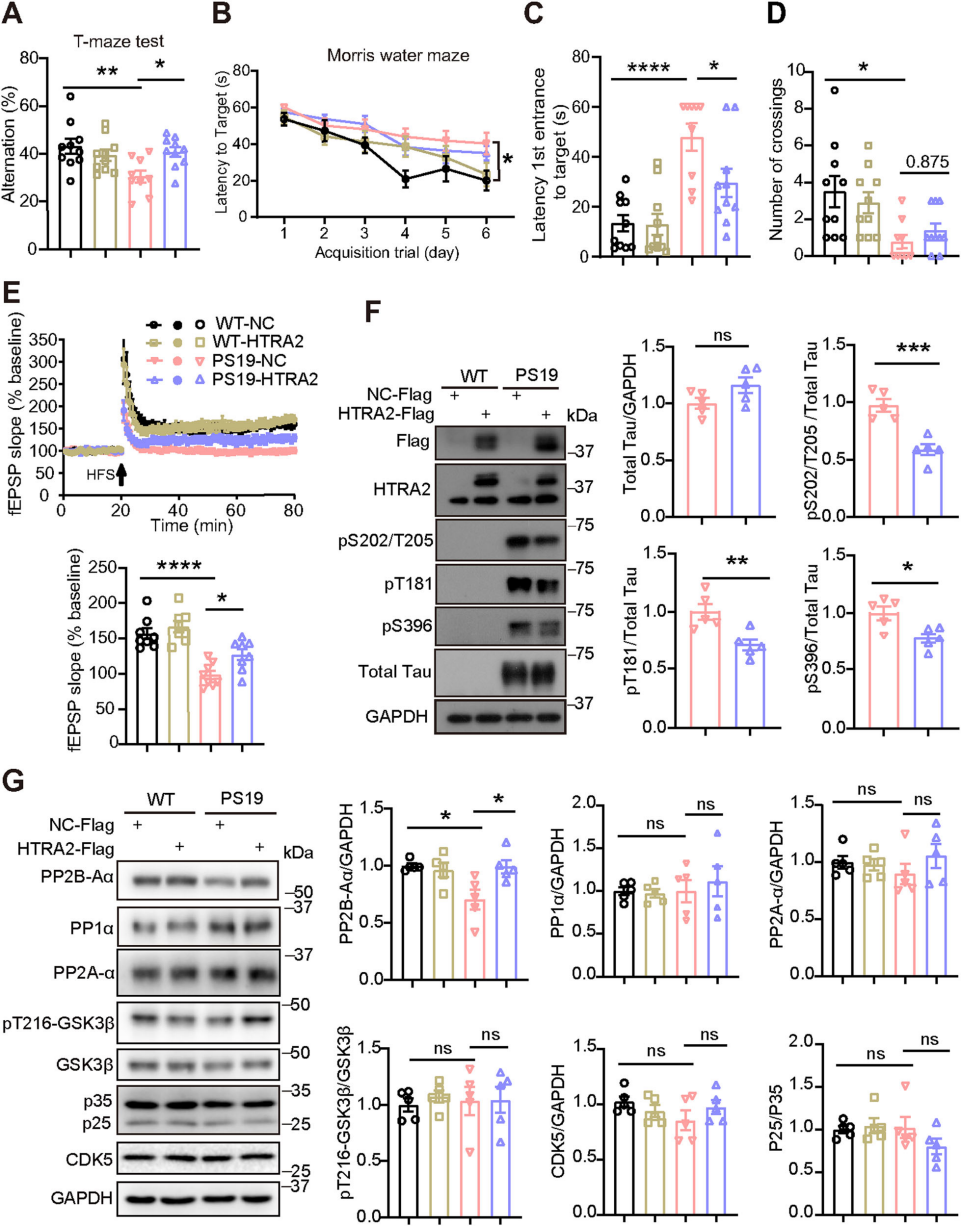
Overexpression of HTRA2 ameliorates tauopathy-related impairments in PS19 mice. **A** In the T maze test, spontaneous alteration percentage was studied for comparison. In the Morris water maze test, the escape latency during a six-day training was recorded for comparison for every day (**B**). On the seventh day, the time spent for the first entry into the target quadrant (**C**) and the number of platform crossings (**D**) were recorded for comparison (WT-NC = 10 mice, WT-HTRA2 = 10 mice, PS19-NC = 9 mice, and PS19-HTRA2 = 10 mice for all behavioral tests). **E** LTP recordings in the hippocampal CA1 region and quantification of the mean fEPSP slope in the last 10 min of recordings (*n* = 8 slices from 4 mice per group). **F** Representative Western blotting of tau and phosphorylated (p-) tau in the hippocampal lysates of treated mice. Comparison of levels of total tau and phosphorylated tau at sites S202/T205, T181, and S396 (*n* = 5 per group). **G** Representative Western blotting of tau kinases and phosphatases in the hippocampal lysates of treated mice. Comparison of levels of PP2B-Aα, PP1α, PP2A-α, phosphorylated GSK3β, CDK5, and P25 (*n* = 5 per group). A significant difference was determined using one-way ANOVA with Tukey’s post hoc analysis (**A**, **C**, **D**, **E**, **G**), two-way ANOVA with Tukey’s post hoc analysis (**B**) and an unpaired *t*-test (**F**). Data represent means ± SEM. *p < 0.05; **p < 0.01; ***p < 0.001; ****p < 0.0001; ns, not significant.

Following the lentivirus injection, we conducted behavioral tests. In the open field test, although the total travel distance of PS19 mice was much more than that of WT mice, HTRA2 overexpression had no effect on the total travel distance of either WT or PS19 mice (Fig. S7C), suggesting that HTRA2 overexpression does not affect locomotor activity. In the novel object recognition test, PS19 mice showed no decreased discrimination index compared to WT mice, and HTRA2 overexpression had no effect on the discrimination index of either WT or PS19 mice (Fig. S7D). In the T maze test, PS19 mice showed reduced spontaneous alteration percentage, whereas HTRA2 overexpression reversed this reduction (Fig. 5A). In the Morris water maze test, PS19 mice showed increased escape latency during a six-day training, and increased latency of the first entry into the target quadrant and decreased numbers of platform crossings during the testing phase on the 7th day (Fig. 5B-D), whereas HTRA2 overexpression significantly decreased the latency of first entry into the target in PS19 mice (Fig. 5C). Together, these results suggest that HTRA2 overexpression partially attenuates memory deficits in PS19 mice.

Moreover, we found that PS19 mice exhibited impaired LTP in the CA3-CA1 pathway, whereas HTRA2 overexpression partially rescued LTP impairment in PS19 mice (Fig. 5E).

### HTRA2 overexpression attenuates PP2B reduction and tau hyperphosphorylation in PS19 mice

Tau hyperphosphorylation is a classic pathological feature in AD and tauopathies and a crucial cause of these neurodegenerative diseases [22, 23]. We measured phosphorylated-tau levels and found that although HTRA2 overexpression did not affect total tau levels, it significantly reduced tau phosphorylation at sites S202/T205, T181, and S396 (Fig. 5F).

The balance of tau phosphorylation is regulated by kinase and phosphatase activities. We assayed several phosphatases and kinases important for tau phosphorylation and observed no changes in protein levels of phosphatases including PP1 subunit α and PP2A subunit α, and of tau kinases including GSK3β T216 phosphorylation form and CDK5 and its cofactors p25/p35 in PS19 mice with HTRA2 overexpression (Fig. 5G). However, we found that protein levels of the PP2B phosphatase catalytic subunit Aα were significantly decreased in PS19 mice and reversed upon HTRA2 overexpression (Fig. 5G). These results implicate that HTRA2 overexpression attenuates tau hyperphosphorylation possibly through promoting the PP2B phosphatase activity.

We also explored the expression pattern of *MAPT*, the gene encoding tau, and the potential correlation between *HTRA2* and *MAPT* expressions in available data. In the (GSE44772) data, *MAPT* expression decreased in AD (Fig. S8A), and *HTRA2* and *MAPT* expressions were positively correlated in controls but not in AD (Fig. S8B). In the RNA-seq data from [21], *MAPT* expression decreased in AD compared to asymptomatic AD but not compared to controls (Fig. S8C), and *HTRA2* and *MAPT* expressions were negatively correlated in controls, asymptomatic AD, and AD (Fig. S8D). In the GEO (GSE13162) data, MA*PT* expression decreased in FTLD-U patients with progranulin mutations compared to those without mutations but not compared to controls (Fig. S8E). However, there was no correlation between *HTRA2* and *MAPT* expressions in controls, FTLD-U patients with progranulin mutations, or those without mutations (Fig. S8F). These inconsistent results implicate that HTRA2 does not affect *MAPT* expression, further supporting that HTRA2 may regulate tau at post-transcriptional levels.

## Discussion

**Fig. 6:**
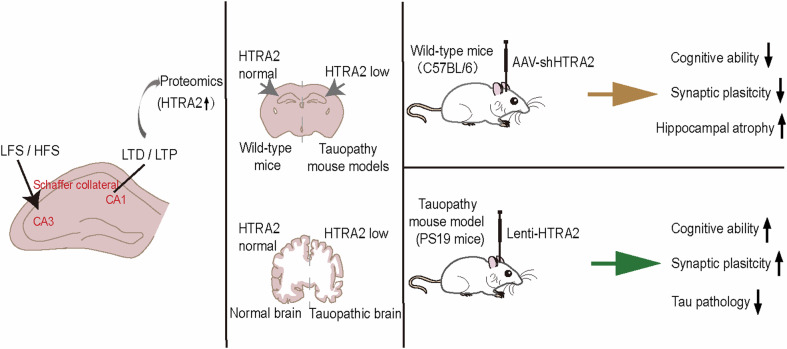
Targeting HTRA2 ameliorates tau pathology in PS19 mice. Long-term potentiation (LTP) and long-term depression (LTD) encode learning and memory through cellular mechanisms. The protein levels of HTRA2 increase in response to HFS-induced LTP and LFS-induced LTD in the postsynaptic neurons. However, the protein levels of HTRA2 decrease in the tauopathy mouse models and AD patients, and downregulation of HTRA2 impairs cognitive ability and synaptic function in wild-type (WT) mice. While overexpression of HTRA2 attenuates cognitive impairment and tau pathology in PS19 mice. These findings suggest the effectiveness of this strategy through showing that an identified protein, HTRA2 can regulate synaptic plasticity and learning and memory in both WT and tauopathy model mice.

Synaptic plasticity is crucial for learning and memory [1]. Synaptic plasticity deficits can cause cognitive impairment and have been associated with various cognitive disorders [2, 3]. However, the detailed molecular mechanisms underlying synaptic plasticity and cognitive disorders have yet to be fully elucidated. We previously developed a novel method to screen genes mediating synaptic plasticity in AD [11]. Herein, based on the LTP and the LTD induction procedures, we expanded this method to screen proteins that regulate synaptic plasticity. We applied HFS and LFS in the hippocampal CA3 region to induce LTP and LTD, respectively, in the hippocampal CA1 region and then collected CA1 tissues for proteomics analysis (Fig. 6). We identified 136 DEPs in LTP and 112 DEPs in LTD. Bioinformatic analysis revealed that LTP-related DEPs are more likely involved in biological processes related to metabolic processes of receptors and mitochondrial respiration, whereas LTD-related DEPs are more likely involved in biological processes related to protein transport. These findings indicate a difference between biological processes involved in LTP and LTD.

We further identified 20 DEPs shared by LTP and LTD. Bioinformatic analysis of the 20 DEPs showed that many were involved in metabolic processes of receptors, endocytosis, and synaptic vesicle cycle, all of which are already known to be important for synaptic plasticity. Moreover, many of them were related to neurodegenerative diseases. For example, HTRA2 has been linked to neurodegenerative diseases such as PD and AD [18–20, 24]. However, the role of HTRA2 in regulating synaptic plasticity remains unclear. Herein, we found that HTRA2 was expressed in various neural cells especially neurons, and that HTRA2 expression was increased in both LTP and LTD. Moreover, HTRA2 downregulation impaired synaptic and mitochondrial functions in cultured primary neurons. AAV-mediated HTRA2 knockdown in the hippocampal neurons also impaired LTP and LTD, and caused learning and memory deficits in WT mice of different ages. These results demonstrate that HTRA2 regulates synaptic plasticity and cognition, possibly through modulating mitochondrial function.

On the other hand, we found that HTRA2 expression was decreased in the brains of AD and FLTD-U patients and different tauopathy model mice but not altered in the brain of 5×FAD mice. The major neuropathological hallmarks of AD are the pathological formation of Aβ-enriched plaques and neurofibrillary tangles comprising hyperphosphorylated tau [25, 26]. Our results suggest that HTRA2 may be associated with tau pathology but not with Aβ pathology. Tauopathy is a group of neurodegenerative diseases characterized by tau pathology and synaptic and cognitive impairments. Therefore, we studied the effect of HTRA2 overexpression on synaptic plasticity and cognitive behaviors in the PS19 tauopathy model mice. We found that overexpression of HTRA2 partially attenuated memory deficits and LTP impairment in PS19 mice. Moreover, HTRA2 overexpression reduced tau hyperphosphorylation.

Tau phosphorylation is dependent on the activities of kinases such as GSK3β and CDK5-P35 and phosphatases such as PP1α, PP2A, and PP2B [27–29]. We found that PP2B-Aα protein levels were decreased in PS19 mice and reversed upon HTRA2 overexpression, implicating that HTRA2 overexpression partially attenuates synaptic plasticity impairment, cognitive deficits, and pathologies of tauopathy model mice possibly by affecting the PP2B phosphatase. PP2B, also called calcineurin, has been suggested to participate in synaptic plasticity, especially LTD, through dephosphorylating AMPA receptors [30, 31]. However, although PP2B is known to dephosphorylate tau [32, 33], studies on PP2B dysregulation in AD brain generated inconsistent results with decreased [34, 35], increased [36], or unaltered [37] PP2B activities all reported. Moreover, it is unclear whether there is a correlation between HTRA2 and PP2B. Therefore, the exact underlying molecular mechanism requires further scrutiny.

Together, in this study we outline a novel strategy for identifying new proteins that can regulate synaptic plasticity. Moreover, we demonstrate the effectiveness of this strategy through showing that an identified protein, HTRA2 can regulate synaptic plasticity and learning and memory in both WT and tauopathy model mice (Fig. 6).

## Supplementary information

Supplemental Fgiure legends

Supplemental Figure 1

Supplemental Figure 2

Supplemental Figure 3

Supplemental Figure 4

Supplemental Figure 5

Supplemental Figure 6

Supplemental Figure 7

Supplemental Figure 8

Supplemental Table 1

Supplemental Table 2

Supplemental Table 3

Supplemental Table 4

raw WB data

## Data Availability

Data are available from the corresponding author on reasonable request.
