# Supplementary material for: Electrophysiology-based screening identifies neuronal HtrA serine peptidase 2 (HTRA2) as a synaptic plasticity regulator participating in tauopathy
Source: Transl Psychiatry. 2025 Jan 10;15:5. doi: 10.1038/s41398-025-03227-4 (PMC11724108; doi:10.1038/s41398-025-03227-4)
Supplement: Supplementary file 1 — Supplemental Fgiure legends [file 41398_2025_3227_MOESM1_ESM.docx]

**Supplemental information**

**Electrophysiology-based screening identifies neuronal HtrA serine peptidase 2 (HTRA2) as a synaptic plasticity regulator participating in tauopathy**

**Naizhen Zheng et al.**

**Supplemental Figure legends**

**Fig. S1** GO and KEGG enrichment analysis of DEPs identified LTP and LTD. (A-D) GO (A, C) and KEGG (B, D) enrichment analysis of DEPs identified in the LTP (A, B) and the LTD (C, D) processes using 8-month-old mice.

**Fig. S2** HTRA2 expression in different neural cells. (A, B) Representative Western blotting (A) and quantification of HTRA2 protein levels (B) in various primary neural cells. NeuN, Iba1, and GFAP are markers for neurons, microglia, and astrocytes, respectively. n=6 independent experiments. A significant difference was determined using one-way ANOVA followed by Tukey’s post hoc test. Data represent means ± SEM. **p* < 0.05; ****p* < 0.001; ns, not significant.

**Fig. S3** AAV-mediated HTRA2 downregulation predominantly localizes in neurons. (A, B) Cultured primary neurons of WT mice were infected with AAV NC, AAV-HTRA2#1, AAV-HTRA2#2, or AAV-HTRA2#3 for 10 days. Equal amounts of protein lysates were immunoblotted (A), and HTRA2 protein levels were quantified for comparison (B) (n=3 per group). (C-E) Representative immunostaining images of NeuN (red in C), GFAP (red in D), and Iba1 (red in E) and staining images of DAPI (blue) in the hippocampal CA1 region of WT mouse brain after injection with AAV-NC and AAV shHTRA2 (represented by EGFP in green). Scale bars, 100μm. Zoom-in image scale bars, 50 μm. A significant difference was determined using one-way ANOVA with Tukey’s post hoc analysis. Data represent means ± SEM. **p* < 0.05; ***p* < 0.01.

**Fig.** **S4** Knockdown of HTRA2 in hippocampal neurons of 2-month-old WT mice impairs synaptic and cognitive function. (A) The scheme of AAV2/9 constructs expressing *HTRA2* shRNA (AAV-shHTRA2) and scrambled negative control shRNA (AAV-shNC) injection in hippocampal CA1 region and experimental procedures. (B) In the open field test, total travel distance was studied for comparison. (C) In the Y maze test, the spontaneous alteration percentage of mice was studied for comparison. (D) In the novel object recognition test, the discrimination index of novel versus familiar objects was studied for comparison. (E-G) In the Morris water maze test, the escape latency during a six-day training was recorded for comparison for every day (E). On the seventh day, the time spent for the first entry into the target quadrant (F) and the number of platform crossings (G) were recorded for comparison. AAV-shNC=15 mice, and AAV-shHTRA2=14 mice for all behavioral tests. (H, I) LTP (H) and LTD (I) recordings in the hippocampal CA1 region and quantification of the mean fEPSP slope in the last 10 min of recordings (n=7 slices from 4 mice per group). (J) Representative Western blotting and quantification comparison of indicated proteins (shNC=4 mice, and shHTRA2=4 mice). (K) Representative images of the hippocampal region of WT mice injected with AAV-shNC or AAV-shHTRA2. Scale bar; 2000 μm. (L) Representative images of brain MRI scan and quantification comparison of the hippocampal region size (shNC=4 mice, and shHTRA2=4 mice). Hippocampal regions were indicated with white dashed lines. A significant difference was determined using two-way ANOVA with Tukey’s post hoc analysis (E), the Mann-Whitney test (C, F, G), and unpaired *t*-test (B, D, H, I, J, L). Data represent means ± SEM. **p* < 0.05; ***p* < 0.01; ns, not significant.

**Fig. S5** Knockdown of HTRA2 in hippocampal neurons of 7-month-old WT mice impairs spontaneous alternation. (A) In the open field test, total travel distance was studied for comparison (shNC=11 mice, and shHTRA2=12 mice). (B) In the Y maze test, the spontaneous alteration percentage of mice was studied for comparison (shNC=11 mice, and shHTRA2=12 mice). A significant difference was determined using the Mann-Whitney test (A), and unpaired *t*-test (B). Data represent means ± SEM. **p* < 0.05; ns, not significant.

**Fig. S6** HTRA2 expression is unaltered in the brain of 5×FAD mice. (A, B) Representative western blotting and quantification of HTRA2 protein levels in the hippocampal (A) and cortical regions (B) of 7-month-old 5×FAD mice and WT controls (n=7 mice per group). A significant difference was determined using unpaired *t*-test. Data represent means ± SEM. ns, not significant.

**Fig. S7** Lentivirus-mediated HTRA2 expression in PS19 mice. (A) The schematic of lentiviral constructs expressing HTRA2 or control (NC) injection in hippocampal CA1 region and experimental analysis. (B) Representative immunostaining images of NeuN (red), GFAP (red), and Iba1 (red) and staining images of DAPI (blue) of the hippocampal CA1 region of 10-month-old PS19 mouse brain after injection with Lenti-HTRA2 (represented by EGFP in green). Scale bar, 50 μm. (C) The schematic of lentiviral constructs expressing HTRA2 or control (NC) injection in hippocampal CA1 region and experimental analysis. (D) In the novel object recognition test, the discrimination index of novel versus familiar objects was studied for comparison (WT-NC=10 mice, WT-HTRA2=10 mice, PS19-NC=9 mice, and PS19-HTRA2=10 mice for all behavioral tests). A significant difference was determined using one-way ANOVA with Tukey’s post hoc analysis (C, D). Data represent means ± SEM. *****p* < 0.0001; ns, not significant.

**Fig. S8** *MAPT* mRNA expression and correlation between *HTRA2* and *MAPT* expressions in tauopathy patients. (A. B) Comparison of *MAPT* mRNA expression (A) and correlation between the mRNA expression of *HTRA2* and *MAPT* (B) in AD patients (n=387) and controls (n=303). Data from NCBI’s Gene Expression Omnibus (Accession number GEO: GSE44772). (C, D) Comparison of *MAPT* mRNA expression (C) in the dorsolateral prefrontal cortex of AD patients (n=198), asymptomatic (Asym) AD patients (n=202), and controls (n=123) and correlation between the mRNA expression of *HTRA2* and *MAPT* (D) in the dorsolateral prefrontal cortex of AD patients (n=197), asymptomatic (Asym) AD patients (n=202), and controls (n=123) from the RNA-seq data from [21]. (E, F) Comparison of *MAPT* mRNA expression (E) and correlation between the mRNA expression of *HTRA2* and *MAPT* (F) in FLTD-U patients with progranulin mutation patients (n=15), FLTD-U patients without progranulin mutation patients (n=24), and controls (n=17). Data from NCBI’s Gene Expression Omnibus (Accession number GEO: GSE13162). A significant difference was determined using Mann-Whitney test (A), one-way ANOVA with Tukey’s post hoc analysis (C), and one-way ANOVA with Dunn’s post hoc analysis (E). Data represent means ± SEM. **p* < 0.05; ****p* < 0.0001; ns, not significant. Pearson correlation analysis was used for correlation analysis. r: correlation coefficient; p: significance value.
