# Supplementary figures and images for: Electrophysiology-based screening identifies neuronal HtrA serine peptidase 2 (HTRA2) as a synaptic plasticity regulator participating in tauopathy
Source: Transl Psychiatry. 2025 Jan 10;15:5. doi: 10.1038/s41398-025-03227-4 (PMC11724108; doi:10.1038/s41398-025-03227-4)

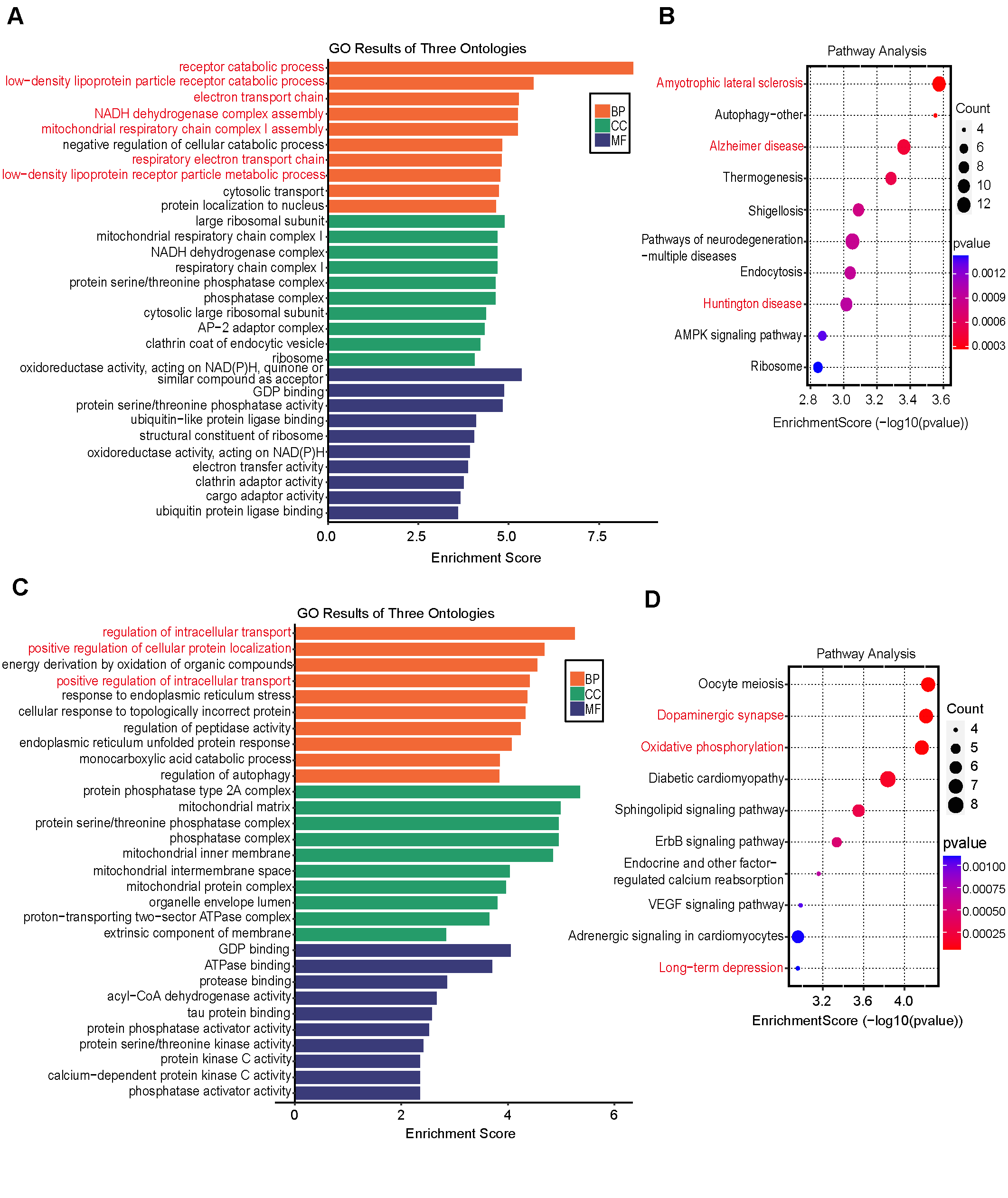

Supplement: Supplementary file 2 — Supplemental Figure 1 [file 41398_2025_3227_MOESM2_ESM.tif]

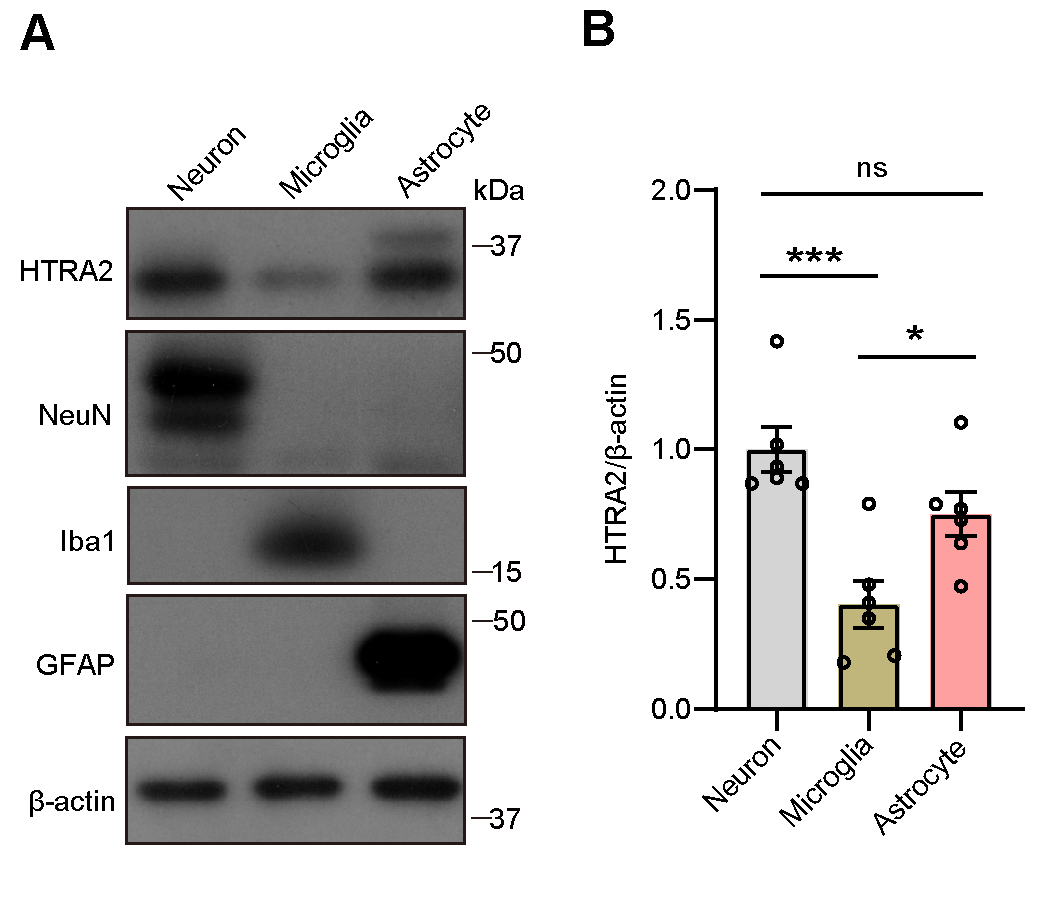

Supplement: Supplementary file 3 — Supplemental Figure 2 [file 41398_2025_3227_MOESM3_ESM.tif]

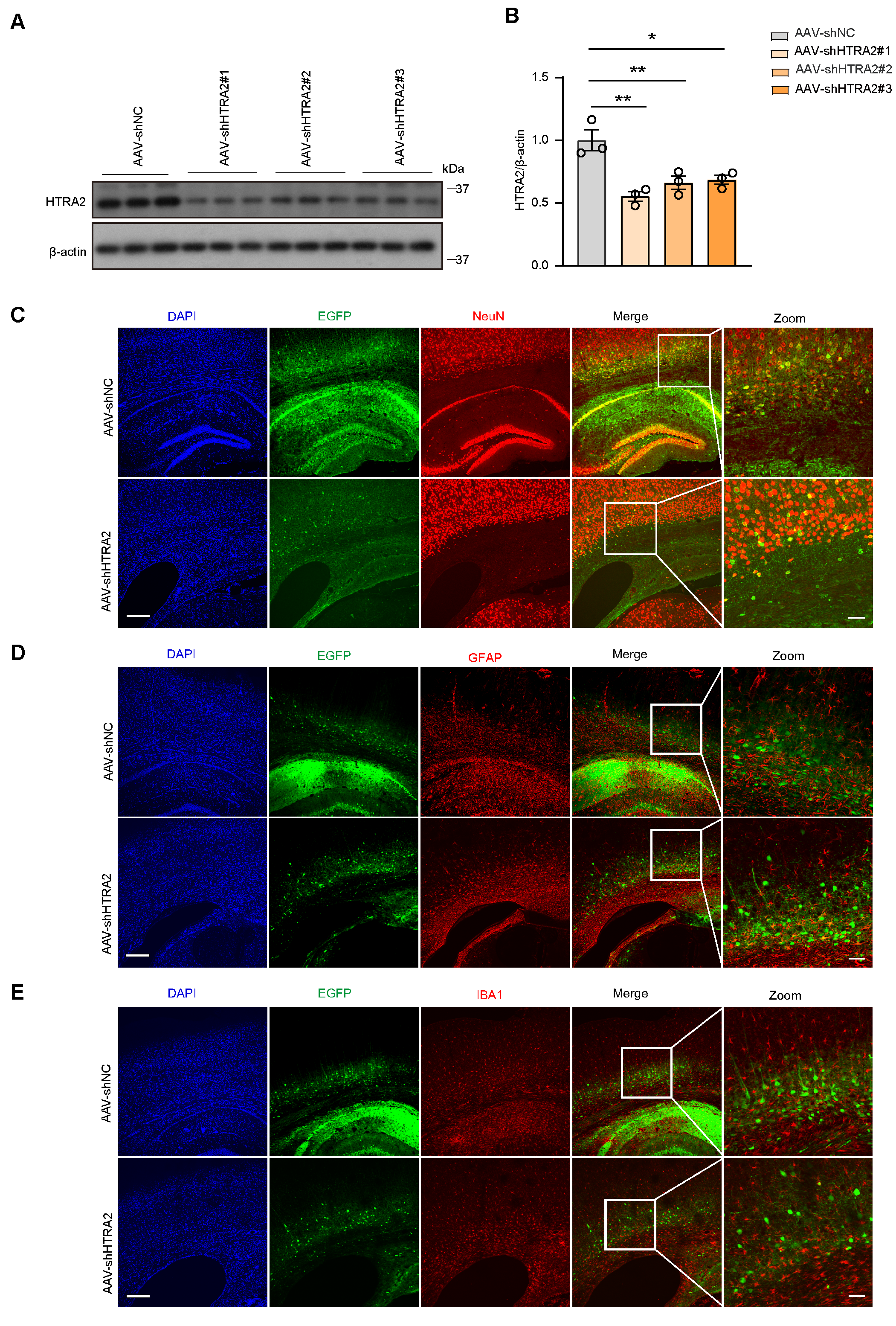

Supplement: Supplementary file 4 — Supplemental Figure 3 [file 41398_2025_3227_MOESM4_ESM.tif]

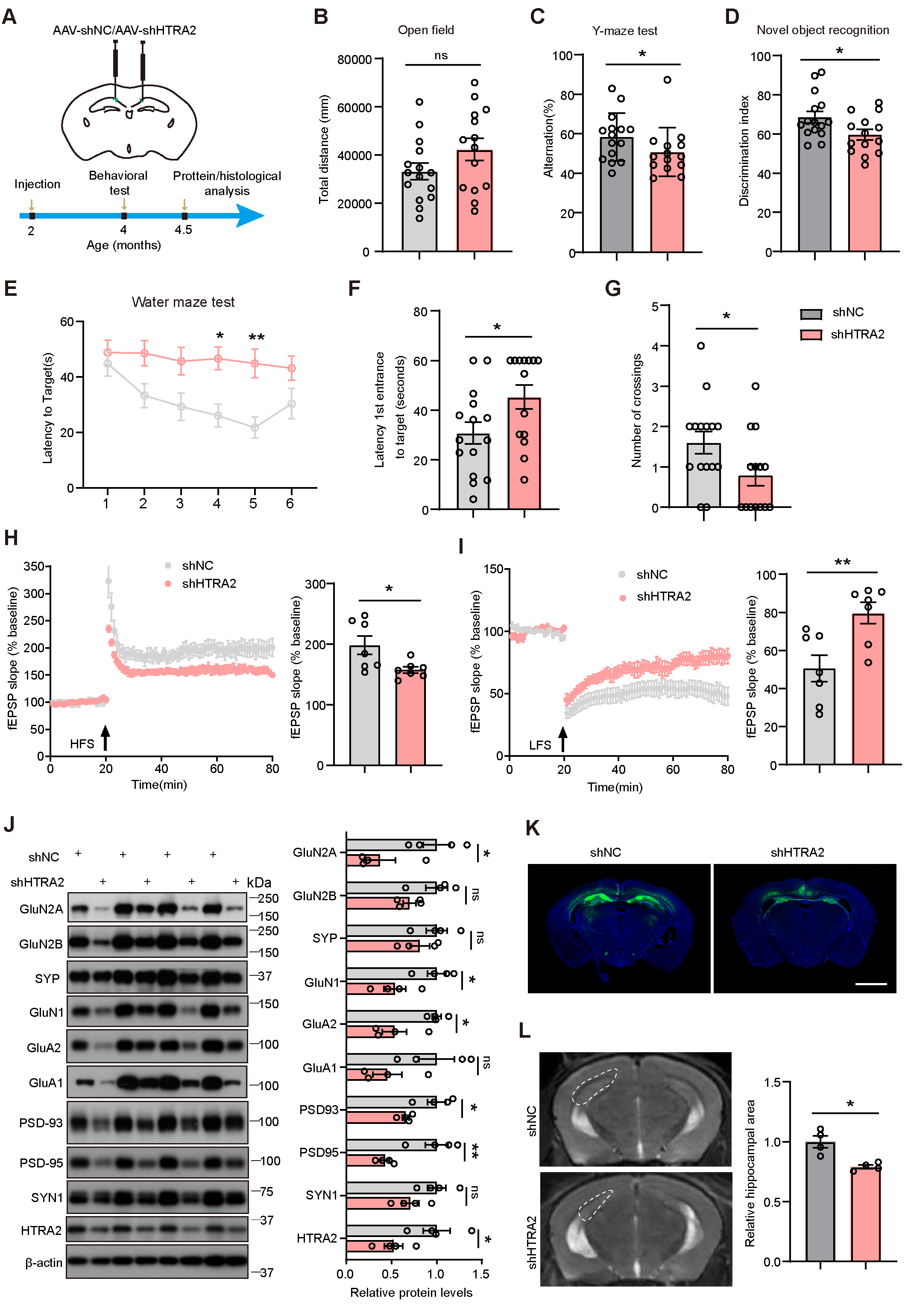

Supplement: Supplementary file 5 — Supplemental Figure 4 [file 41398_2025_3227_MOESM5_ESM.tif]

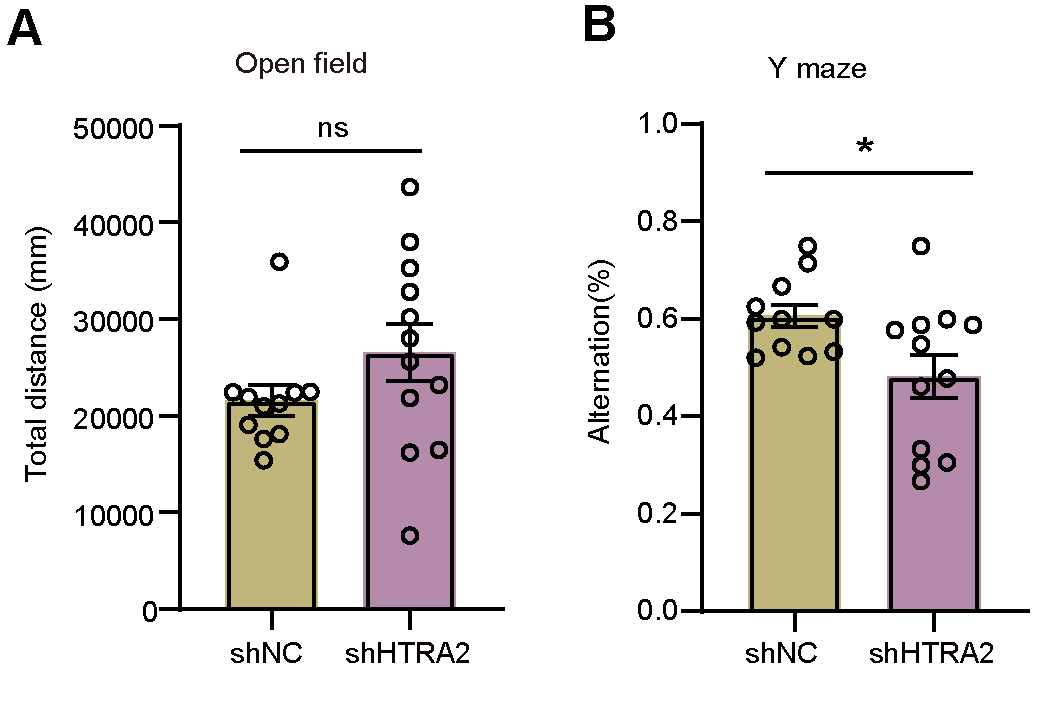

Supplement: Supplementary file 6 — Supplemental Figure 5 [file 41398_2025_3227_MOESM6_ESM.tif]

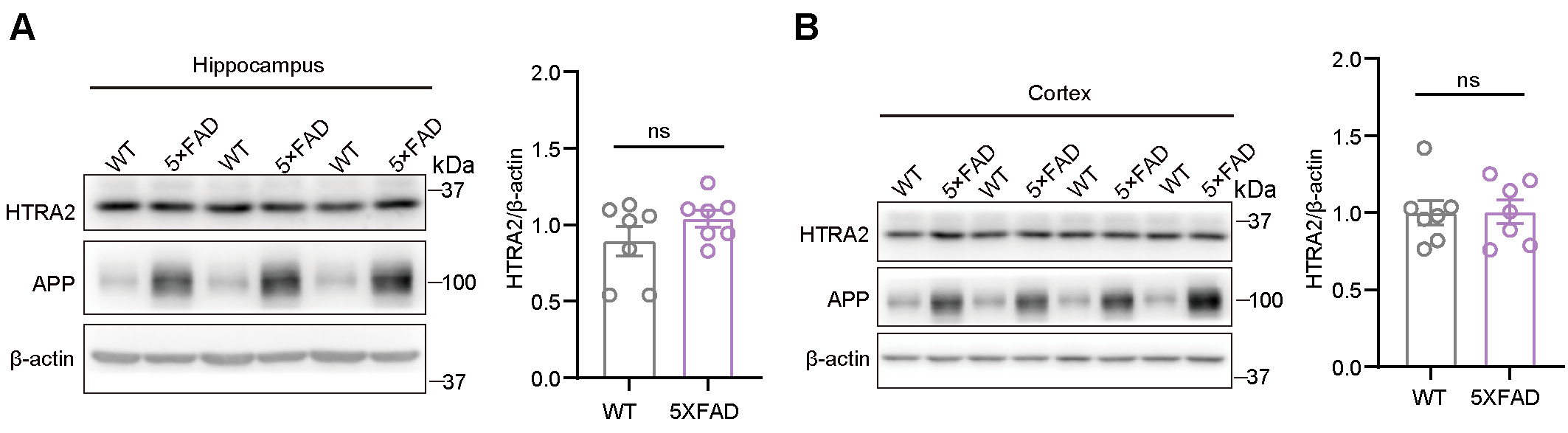

Supplement: Supplementary file 7 — Supplemental Figure 6 [file 41398_2025_3227_MOESM7_ESM.tif]

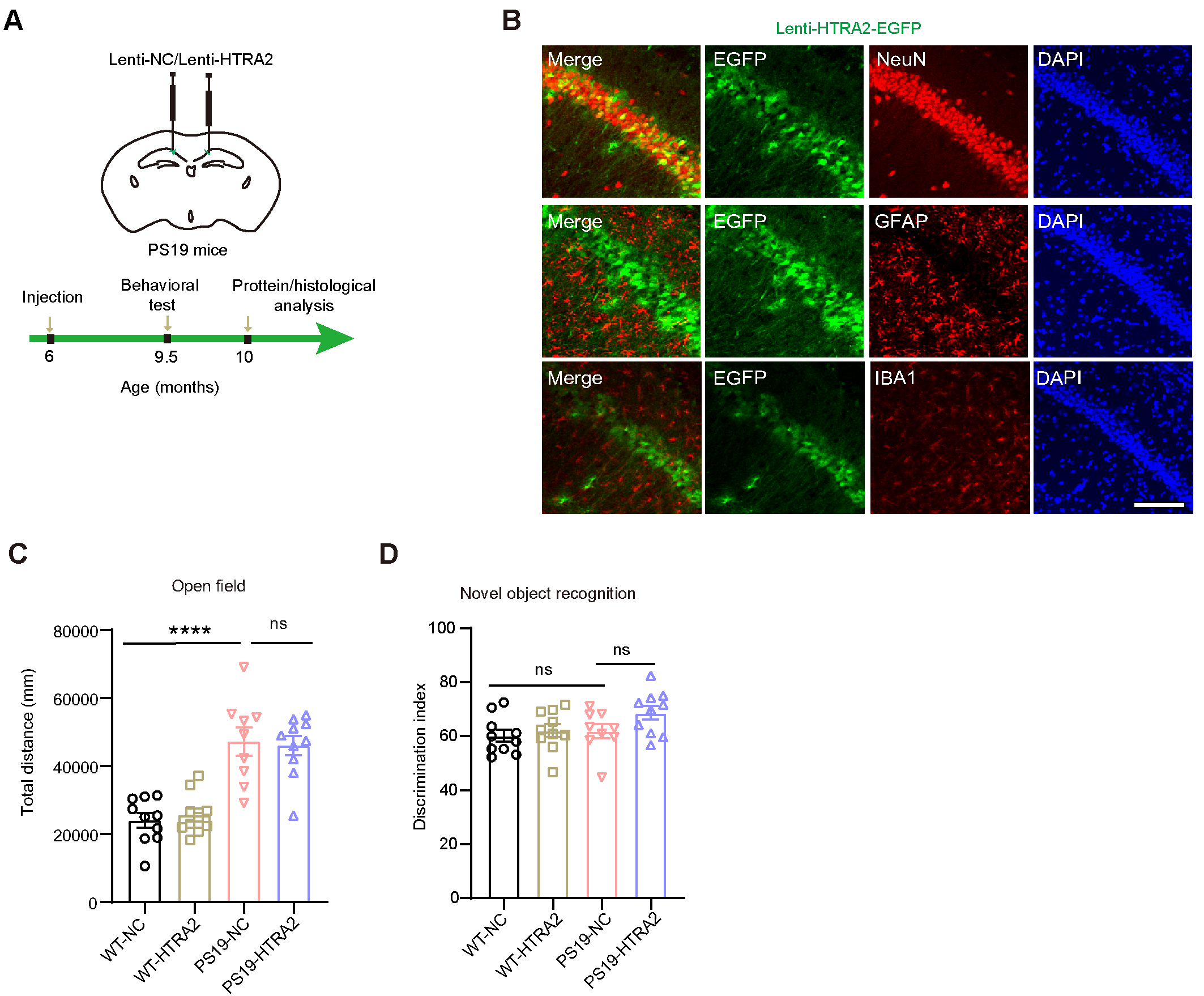

Supplement: Supplementary file 8 — Supplemental Figure 7 [file 41398_2025_3227_MOESM8_ESM.tif]

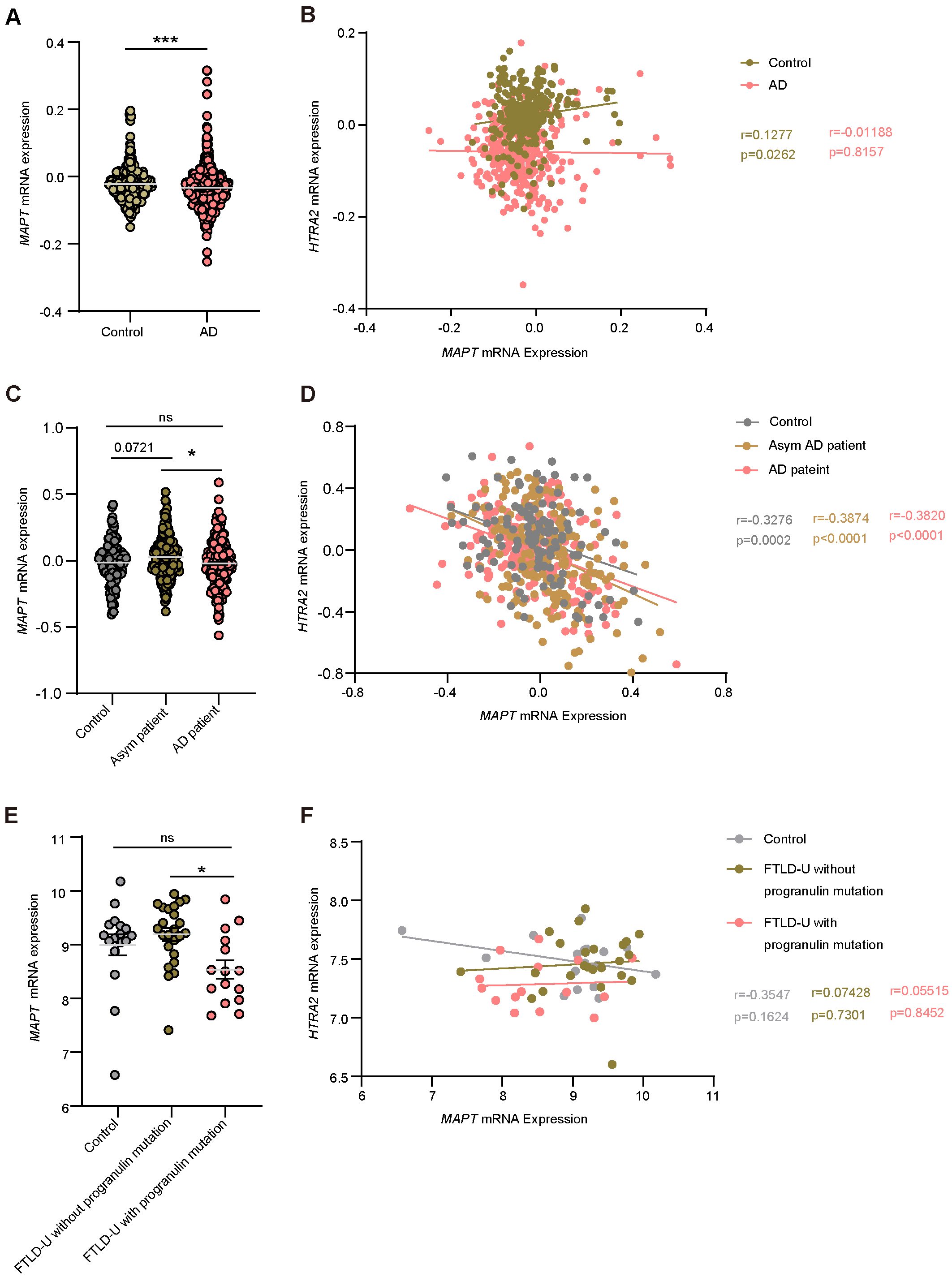

Supplement: Supplementary file 9 — Supplemental Figure 8 [file 41398_2025_3227_MOESM9_ESM.tif]
