# Supplementary material for: Electrophysiology-based screening identifies neuronal HtrA serine peptidase 2 (HTRA2) as a synaptic plasticity regulator participating in tauopathy
Source: Transl Psychiatry. 2025 Jan 10;15:5. doi: 10.1038/s41398-025-03227-4 (PMC11724108; doi:10.1038/s41398-025-03227-4)
Supplement: Supplementary file 10 — Supplemental Table 1 [file 41398_2025_3227_MOESM10_ESM.docx]

**Supplemental Table S1 AD and control brain sample information**

| **NO.** | **Case ID** | **Primary neuropathologic diagnosis** | **Age of death** | **Gender** | **Braak stages** | **Sample** |
| --- | --- | --- | --- | --- | --- | --- |
| 1 | 81 | Control | 80 | Female | N/A | brain tissue lysates |
| 2 | 157 | Control | 86 | Male |  |  |
| 3 | 174 | Control | 93 | Male |  |  |
| 4 | 129 | AD | 83 | Male |  |  |
| 5 | 194 | AD | 93 | Male |  |  |
| 6 | 297 | AD | 85 | Female |  |  |
| 7 | 2019CBB024 | Control | 75 | Male |  |  |
| 8 | 2018CBB006 | Control | 84 | Female |  |  |
| 9 | BB033 | AD | 81 | Male |  |  |
| 10 | BB034 | AD | 75 | Male |  |  |
| 11 | BB010 | AD | 97 | Female |  |  |
