# Supplementary material for: Electrophysiology-based screening identifies neuronal HtrA serine peptidase 2 (HTRA2) as a synaptic plasticity regulator participating in tauopathy
Source: Transl Psychiatry. 2025 Jan 10;15:5. doi: 10.1038/s41398-025-03227-4 (PMC11724108; doi:10.1038/s41398-025-03227-4)
Supplement: Supplementary file 13 — Supplemental Table 4 [file 41398_2025_3227_MOESM13_ESM.docx]

**Supplemental Table S4 DEPs shared by LTP and LTD processes**

| **Primary_protein_ID** | **change in LTP** | **change in LTD** |
| --- | --- | --- |
| sp\|Q6P5G6\|UBXN7_MOUSE | up | up |
| sp\|Q8VDV8\|MITD1_MOUSE | up | up |
| sp\|Q9D666\|SUN1_MOUSE | up | up |
| sp\|Q9D6J6\|NDUV2_MOUSE | up | up |
| sp\|Q9JIY5\|HTRA2_MOUSE | up | up |
| sp\|E9Q6P5\|TTC7B_MOUSE | down | down |
| sp\|P05480\|SRC_MOUSE | down | down |
| sp\|P27046\|MA2A1_MOUSE | down | down |
| sp\|P35293\|RAB18_MOUSE | down | down |
| sp\|P53612\|PGTB2_MOUSE | down | down |
| sp\|P62743\|AP2S1_MOUSE | down | down |
| sp\|P97470\|PP4C_MOUSE | down | down |
| sp\|Q61151\|2A5E_MOUSE | down | down |
| sp\|Q8K2Q5\|CHCH7_MOUSE | down | down |
| sp\|Q8R349\|CDC16_MOUSE | down | down |
| sp\|Q91Z83\|MYH7_MOUSE | down | down |
| sp\|Q923D4\|SF3B5_MOUSE | down | down |
| sp\|Q9DBG3\|AP2B1_MOUSE | down | down |
| sp\|Q9DC63\|FBX3_MOUSE | down | down |
| sp\|Q9Z1W9\|STK39_MOUSE | down | down |
