# Supplementary material for: Electrophysiology-based screening identifies neuronal HtrA serine peptidase 2 (HTRA2) as a synaptic plasticity regulator participating in tauopathy
Source: Transl Psychiatry. 2025 Jan 10;15:5. doi: 10.1038/s41398-025-03227-4 (PMC11724108; doi:10.1038/s41398-025-03227-4)
Supplement: Supplementary file 14 — raw WB data [file 41398_2025_3227_MOESM14_ESM.pdf]

Full unedited gel for Figure 2G

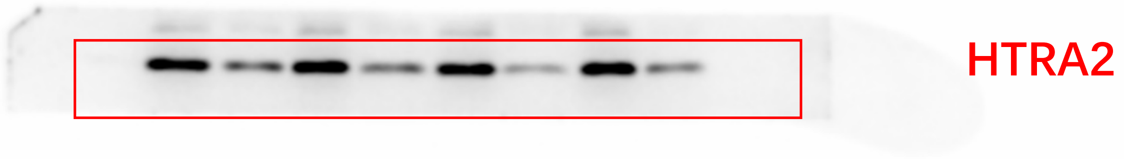

The membrane was imaged with Azure Biosystems 300

Full unedited gel for Figure 2G

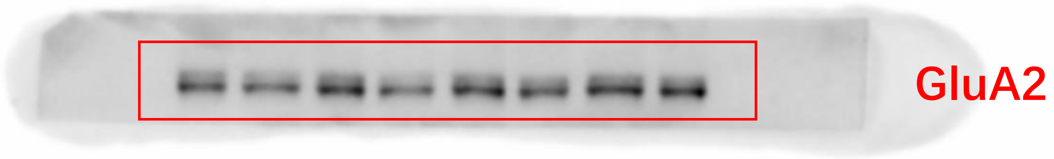

The membrane was imaged with Azure Biosystems 300

Full unedited gel for Figure 2G

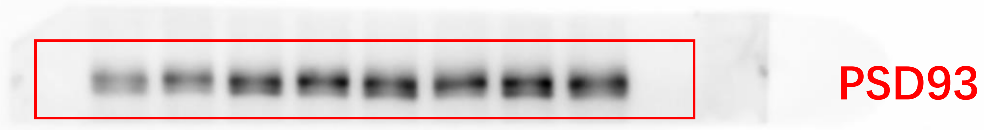

The membrane was imaged with Azure Biosystems 300

Full unedited gel for Figure 2G

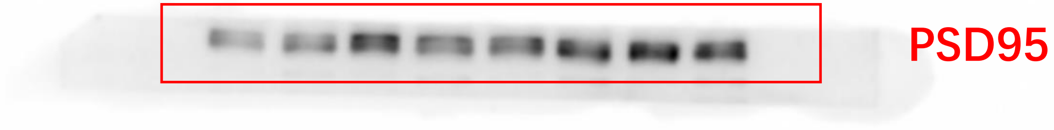

The membrane was imaged with Azure Biosystems 300

Full unedited gel for Figure 2G

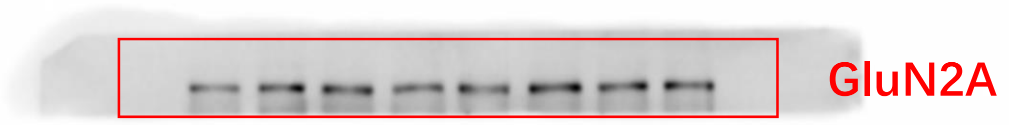

The membrane was imaged with Azure Biosystems 300

Full unedited gel for Figure 2G

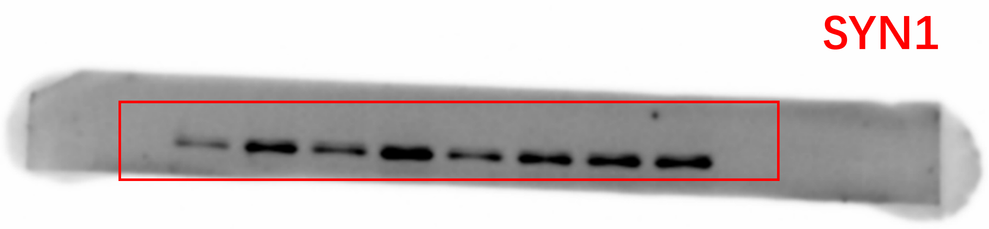

The membrane was imaged with Azure Biosystems 300

Full unedited gel for Figure 2G

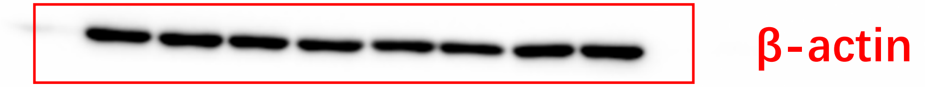

The membrane was imaged with Azure Biosystems 300

Full unedited gel for Figure 3I

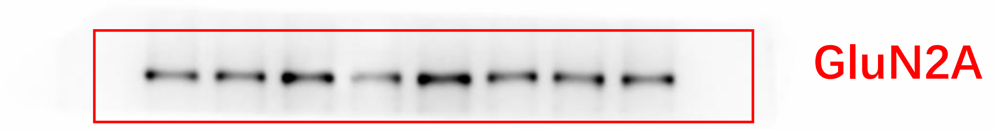

The membrane was imaged with Azure Biosystems 300

Full unedited gel for Figure 3I

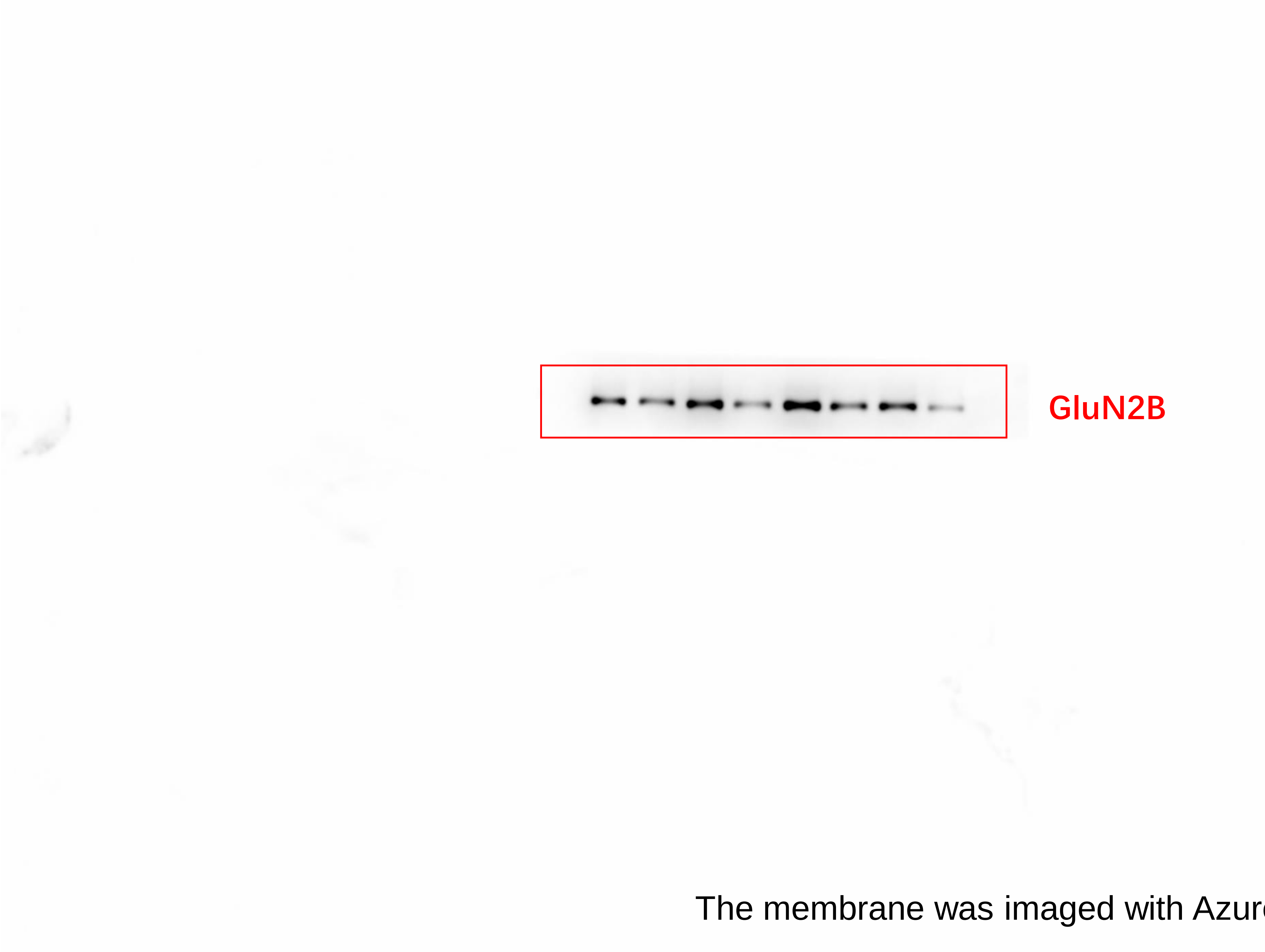

The membrane was imaged with Azure Biosystems 300

Full unedited gel for Figure 3I

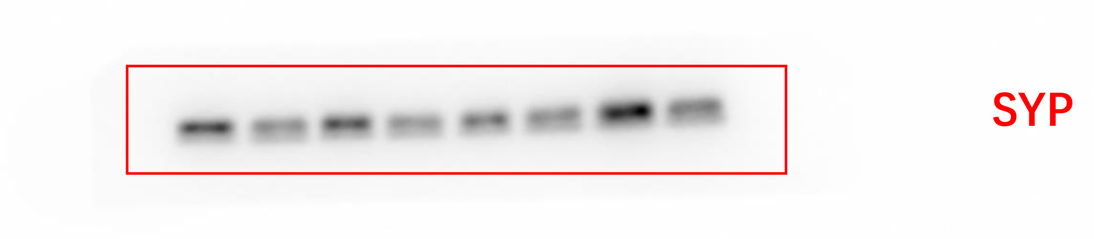

The membrane was imaged with Azure Biosystems 300

Full unedited gel for Figure 3I

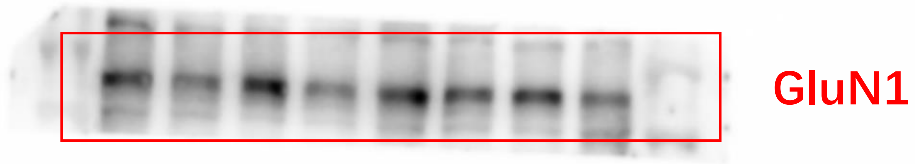

The membrane was imaged with Azure Biosystems 300

Full unedited gel for Figure 3I

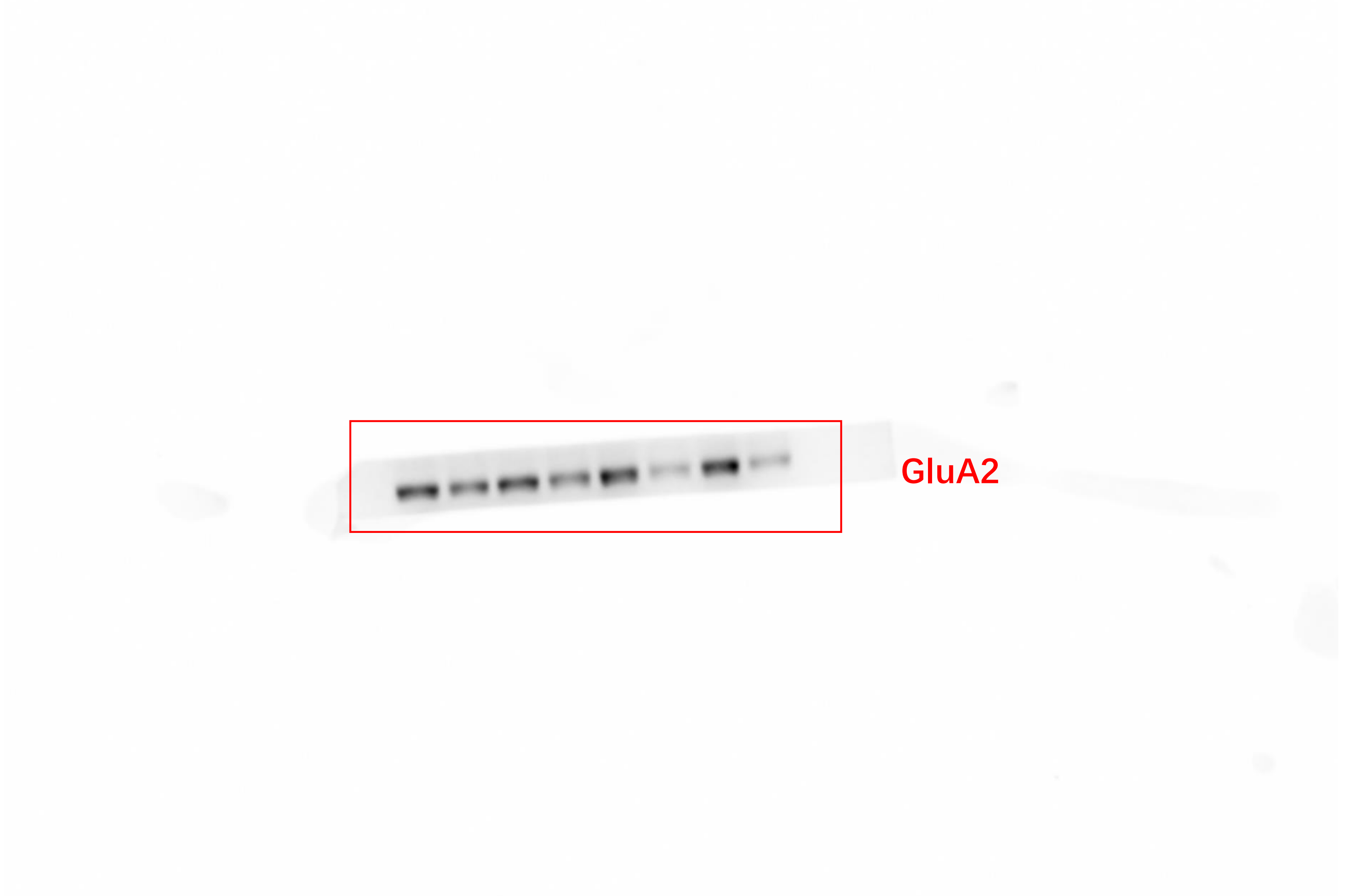

The membrane was imaged with Azure Biosystems 300

Full unedited gel for Figure 3I

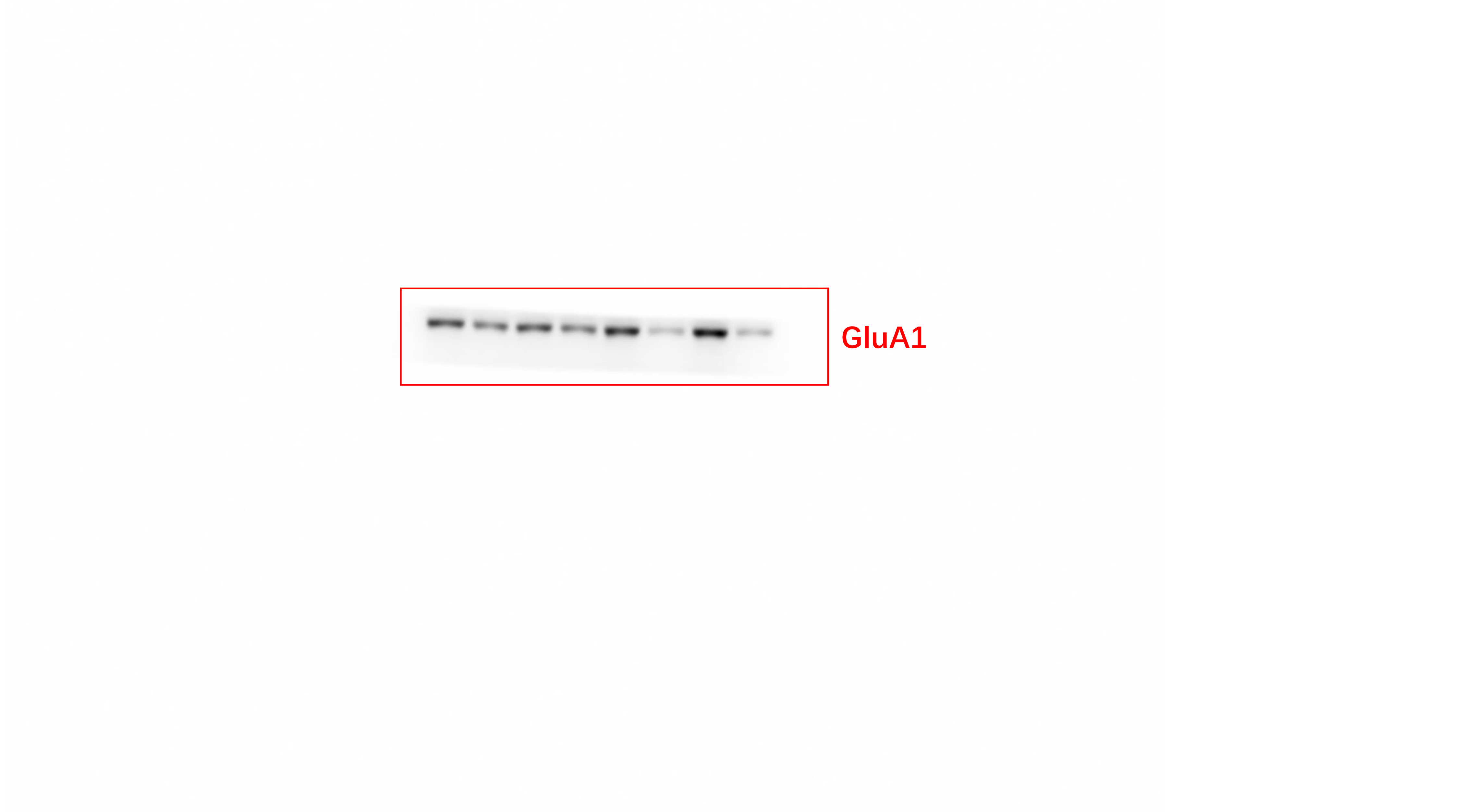

The membrane was imaged with Azure Biosystems 300

Full unedited gel for Figure 3I

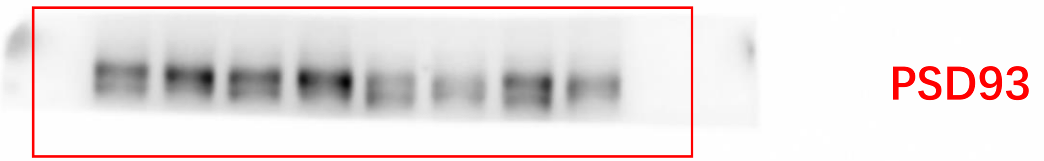

The membrane was imaged with Azure Biosystems 300

Full unedited gel for Figure 3I

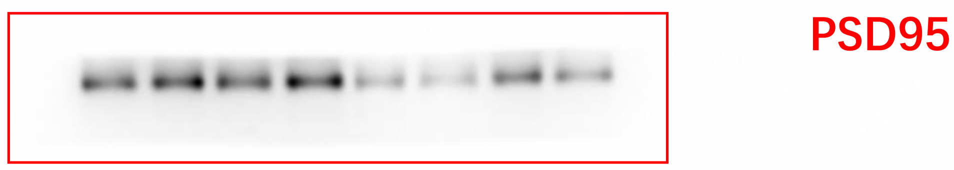

The membrane was imaged with Azure Biosystems 300

Full unedited gel for Figure 3I

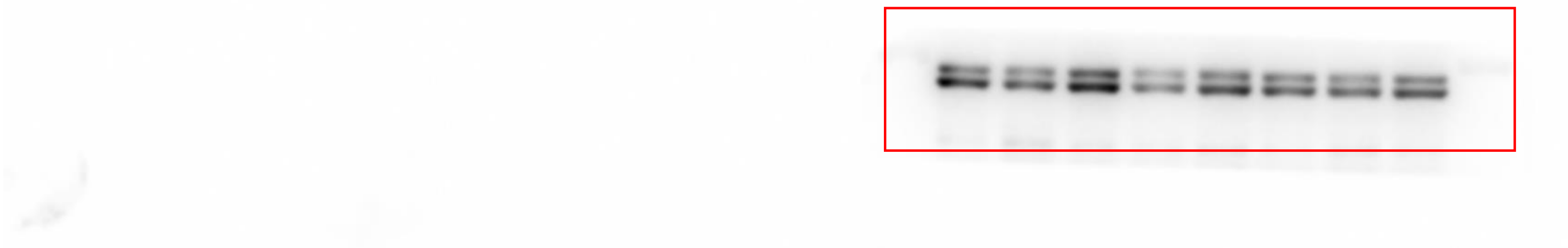

The membrane was imaged with Azure Biosystems 300

Full unedited gel for Figure 3I

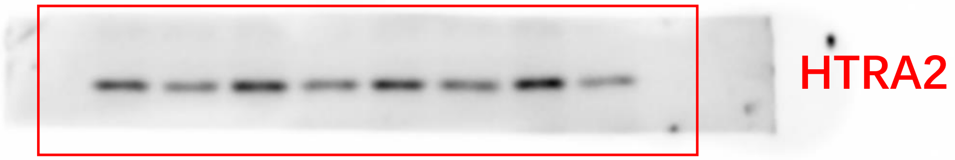

The membrane was imaged with Azure Biosystems 300

Full unedited gel for Figure 3I

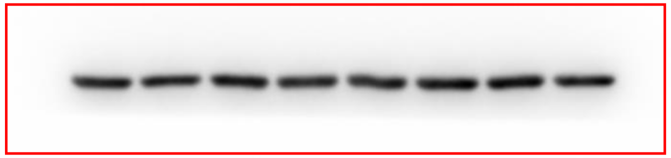

The membrane was imaged with Azure Biosystems 300

Full unedited gel for Figure 4D

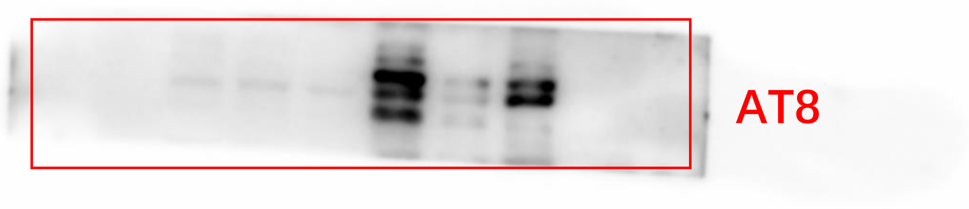

The membrane was imaged with Azure Biosystems 300

Full unedited gel for Figure 4D

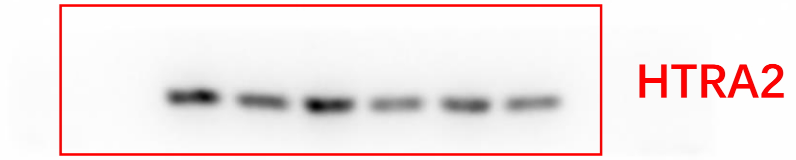

The membrane was imaged with Azure Biosystems 300

Full unedited gel for Figure 4D

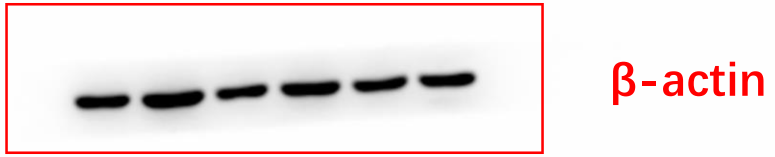

The membrane was imaged with Azure Biosystems 300

Full unedited gel for Figure 4F

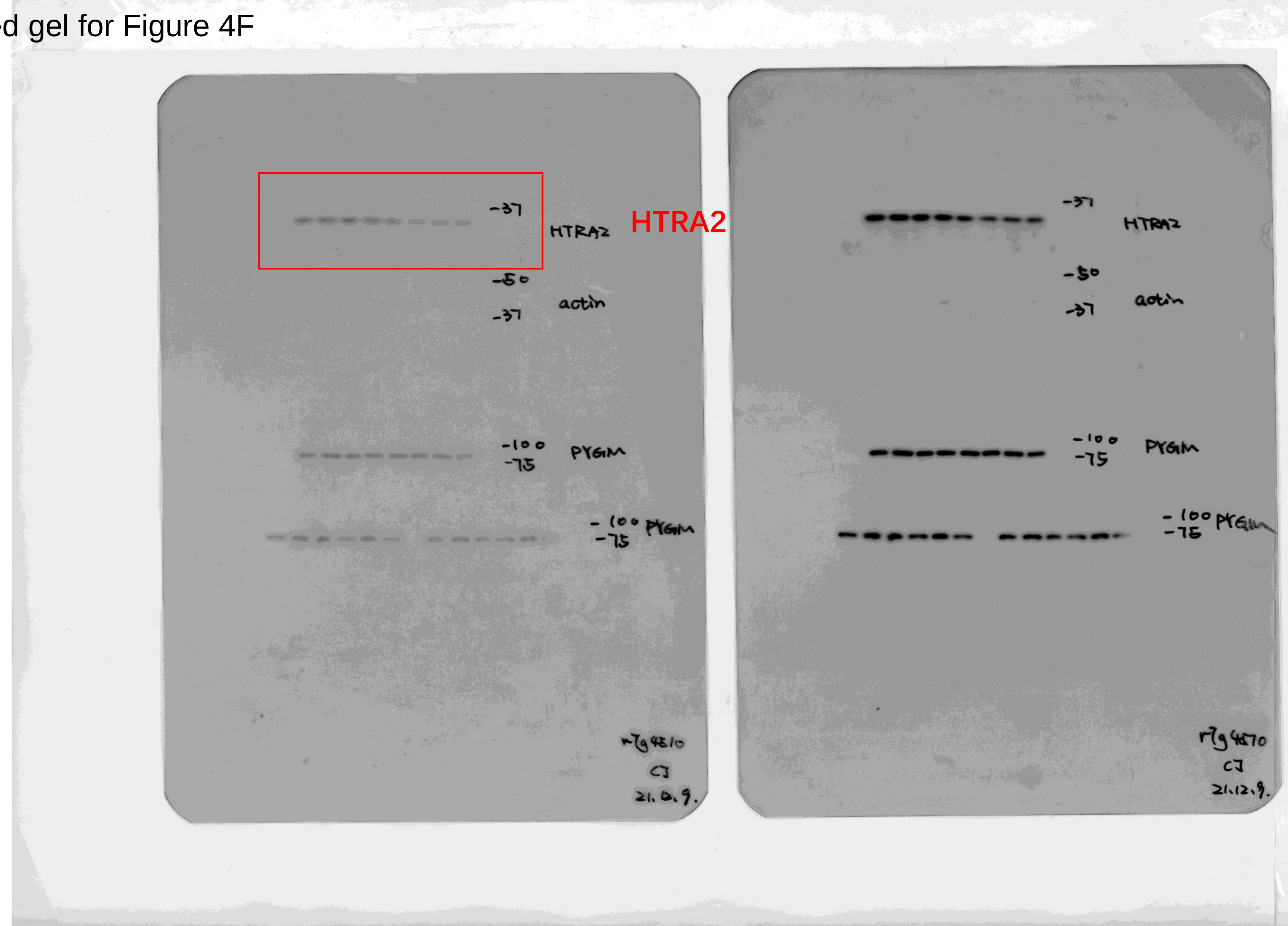

Full unedited gel for Figure 4F

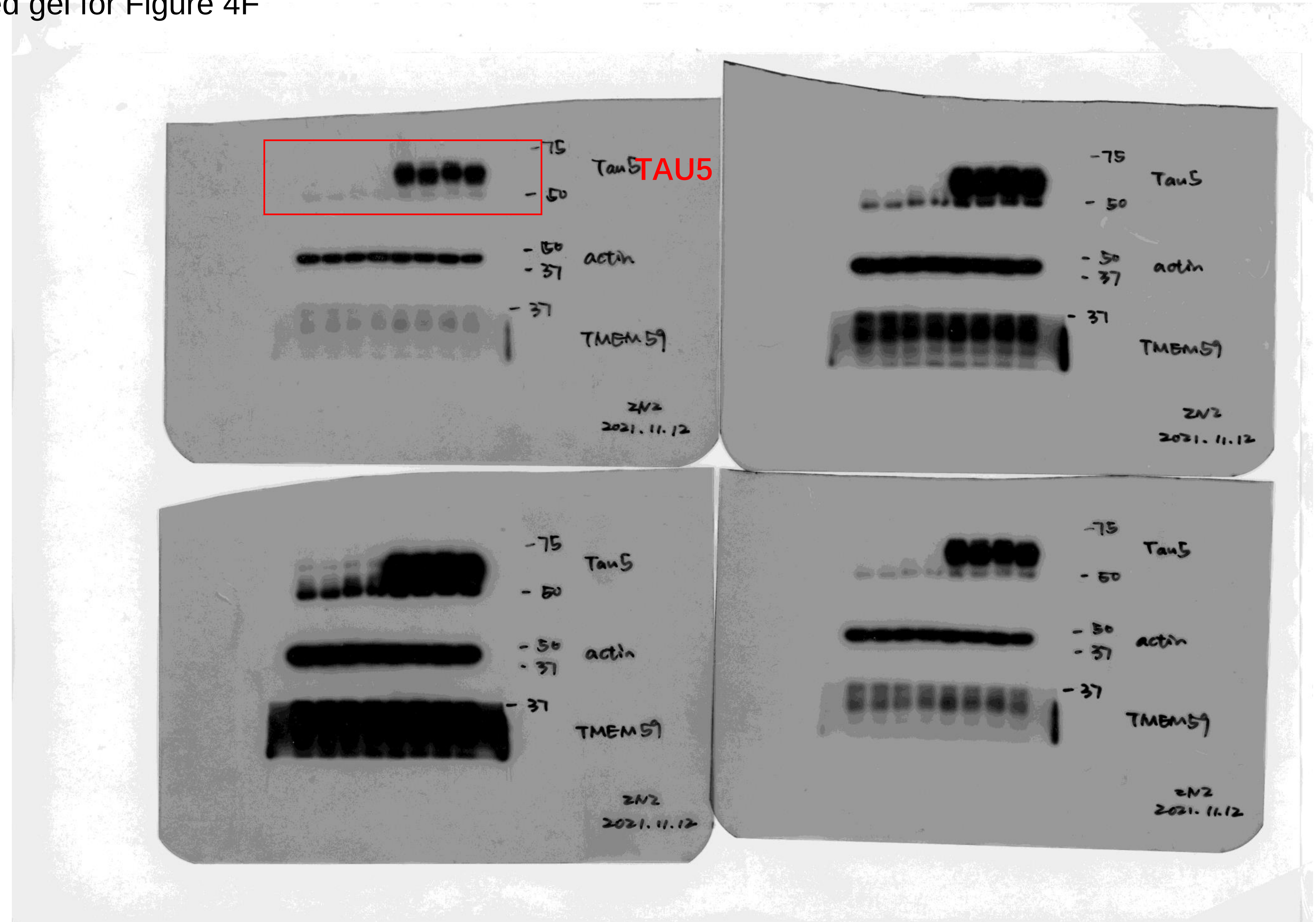

Full unedited gel for Figure 4F

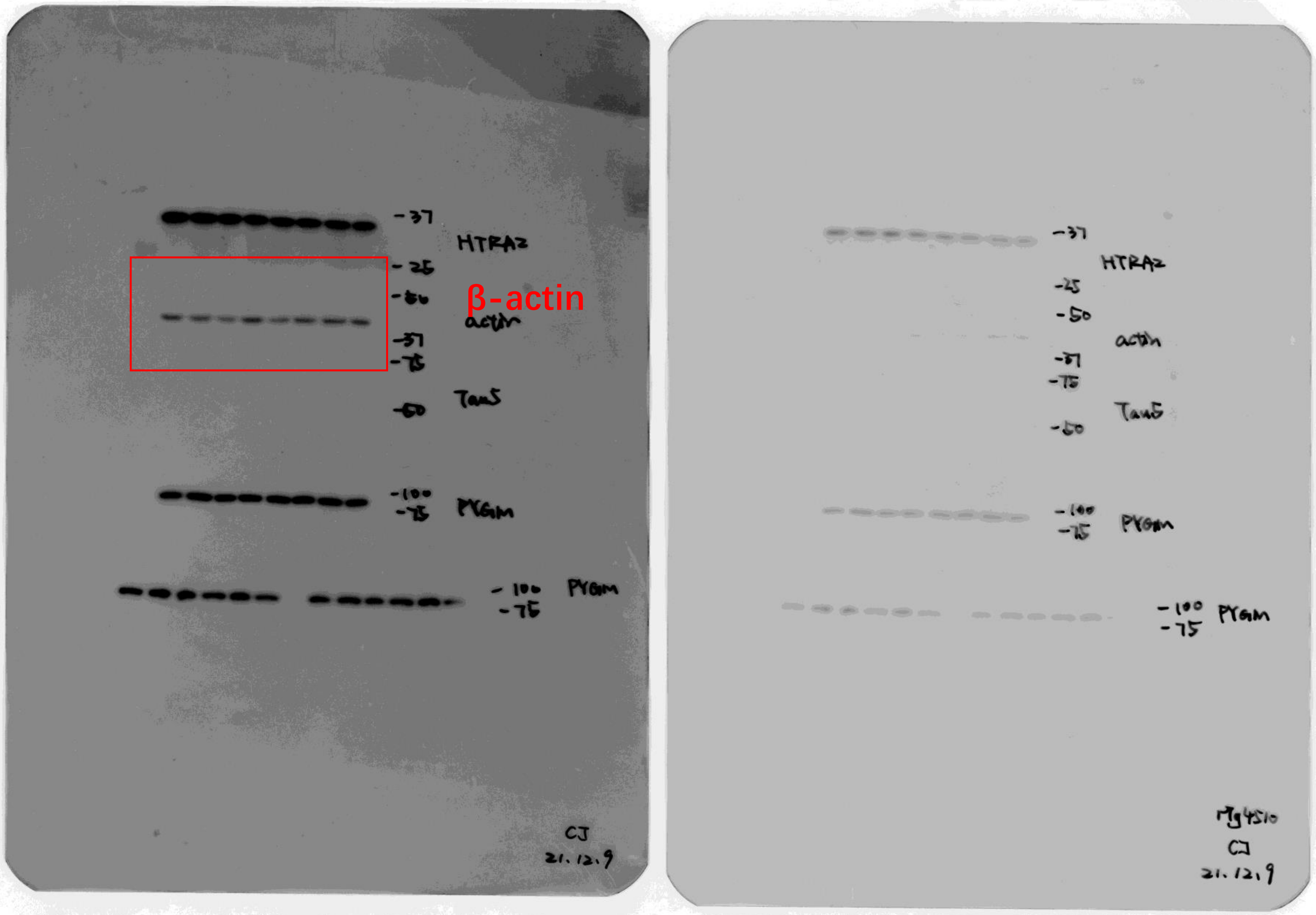

Full unedited gel for Figure 4G

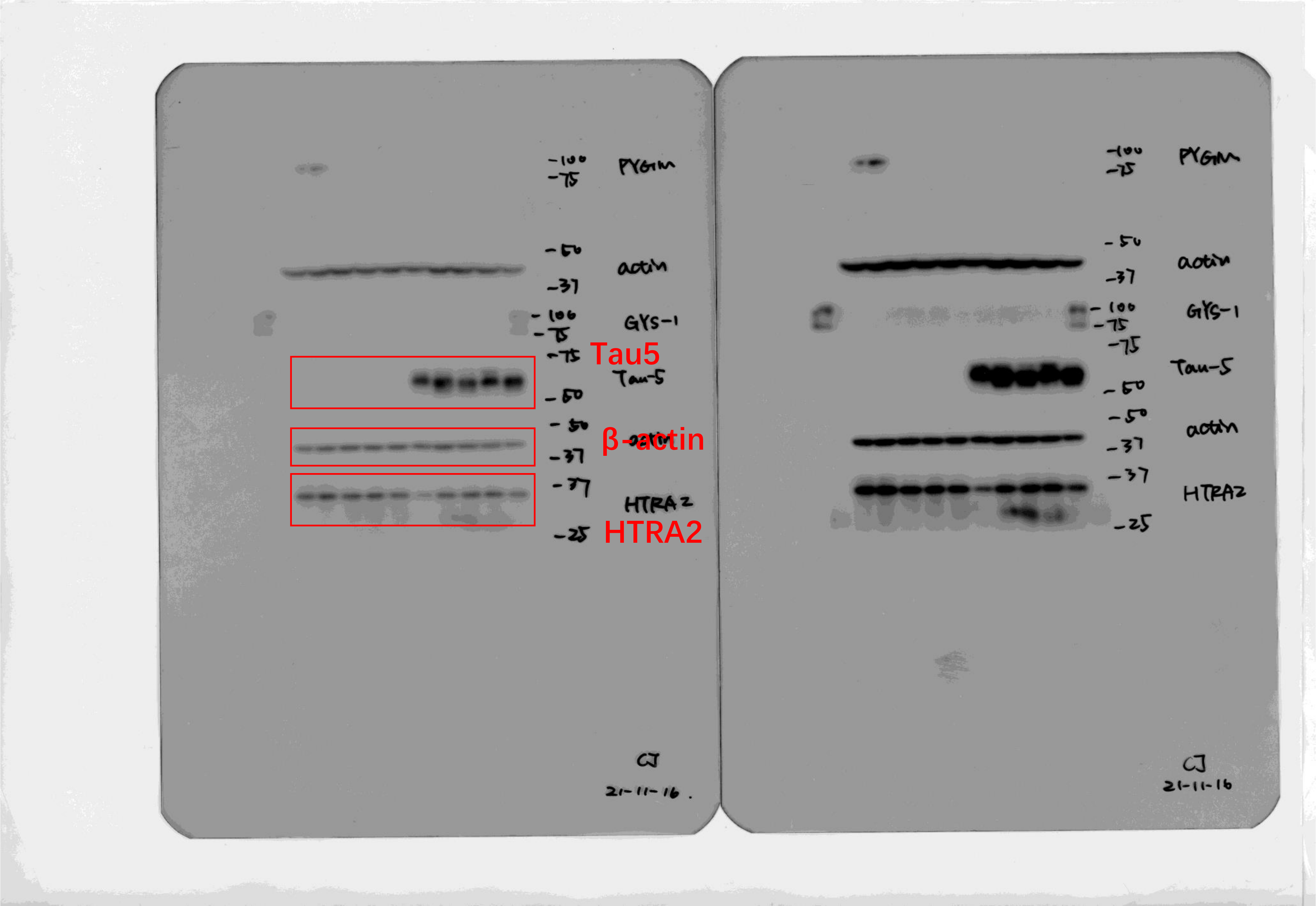

Full unedited gel for Figure 5F

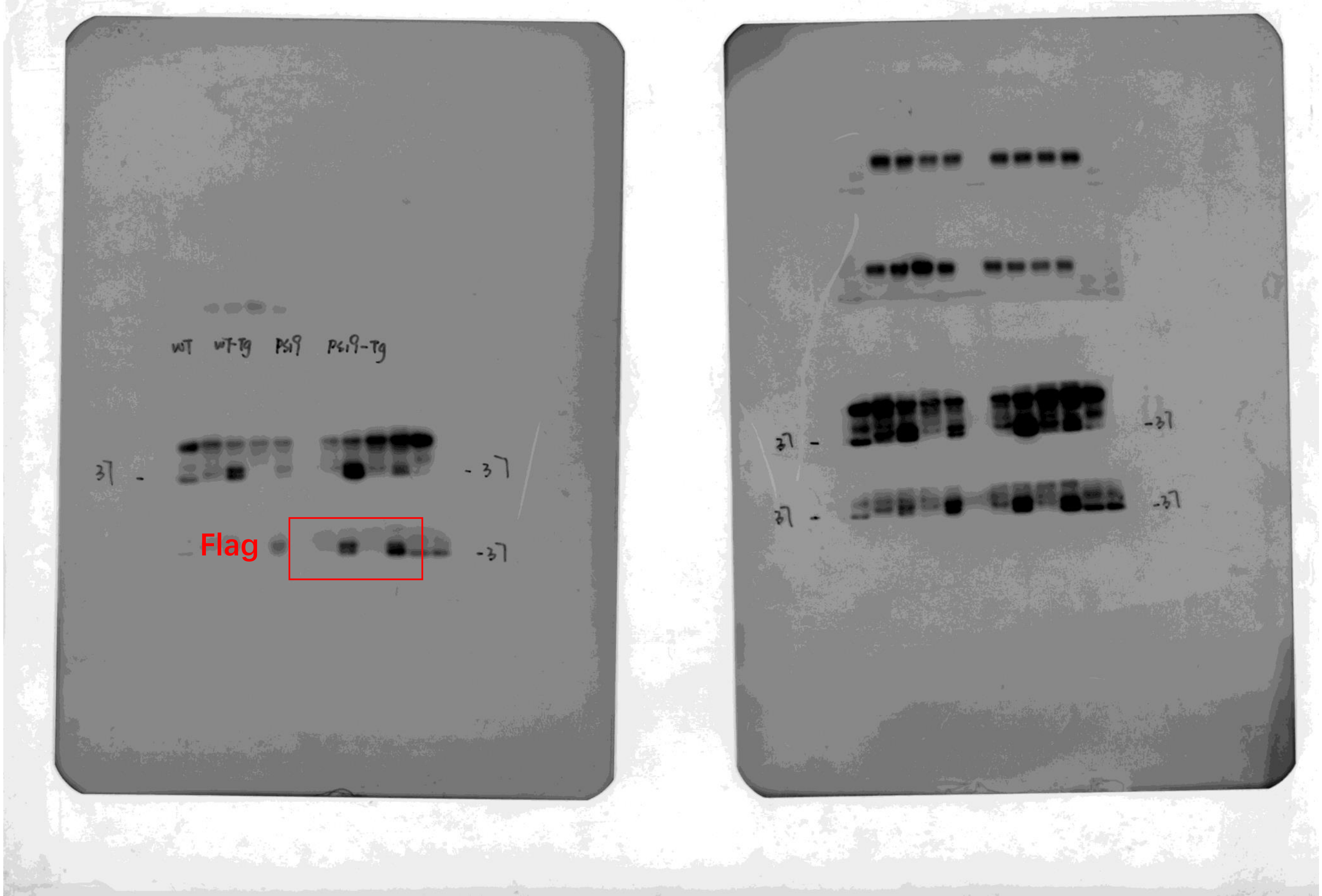

Full unedited gel for Figure 5F

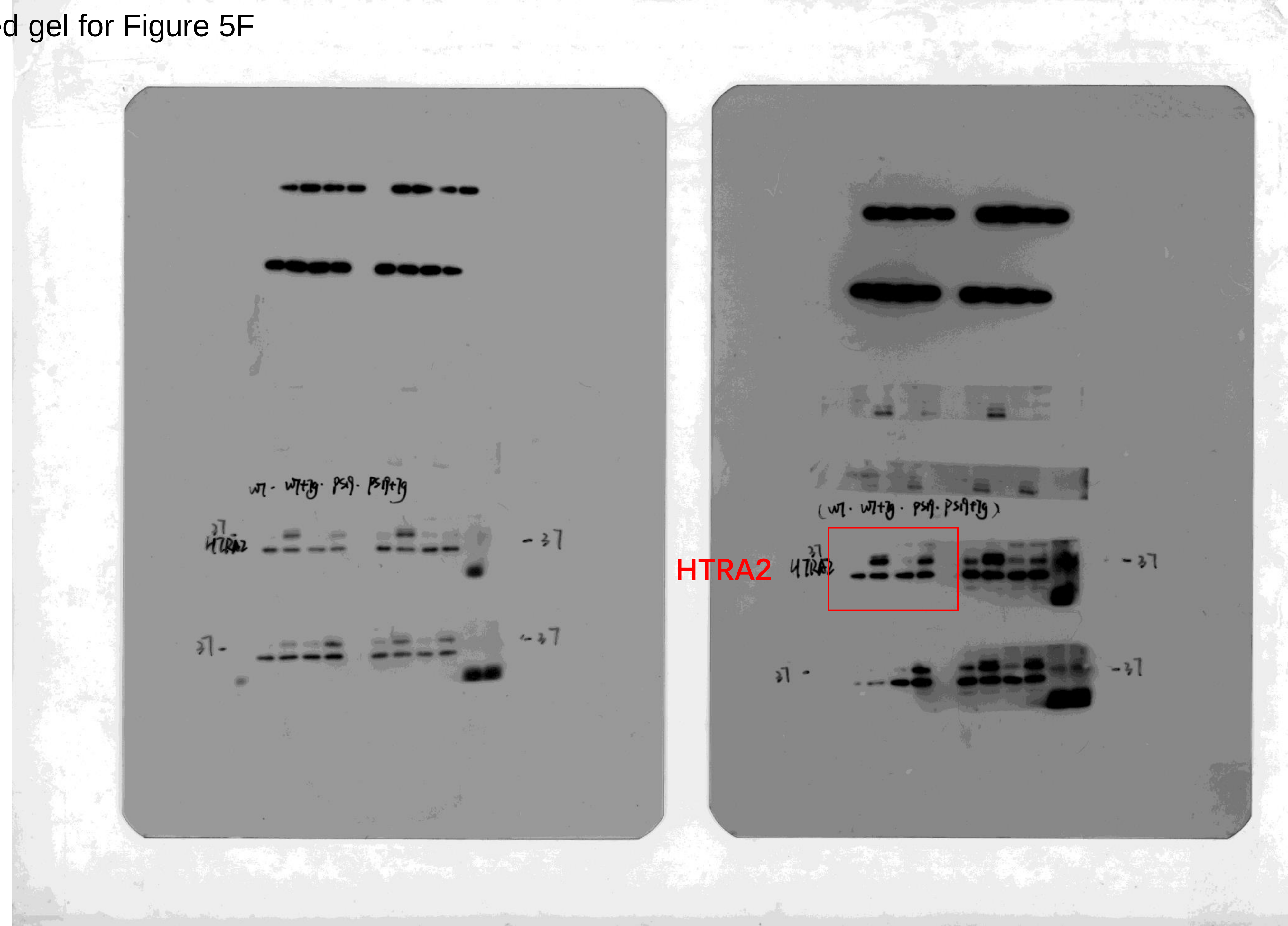

Full unedited gel for Figure 5F

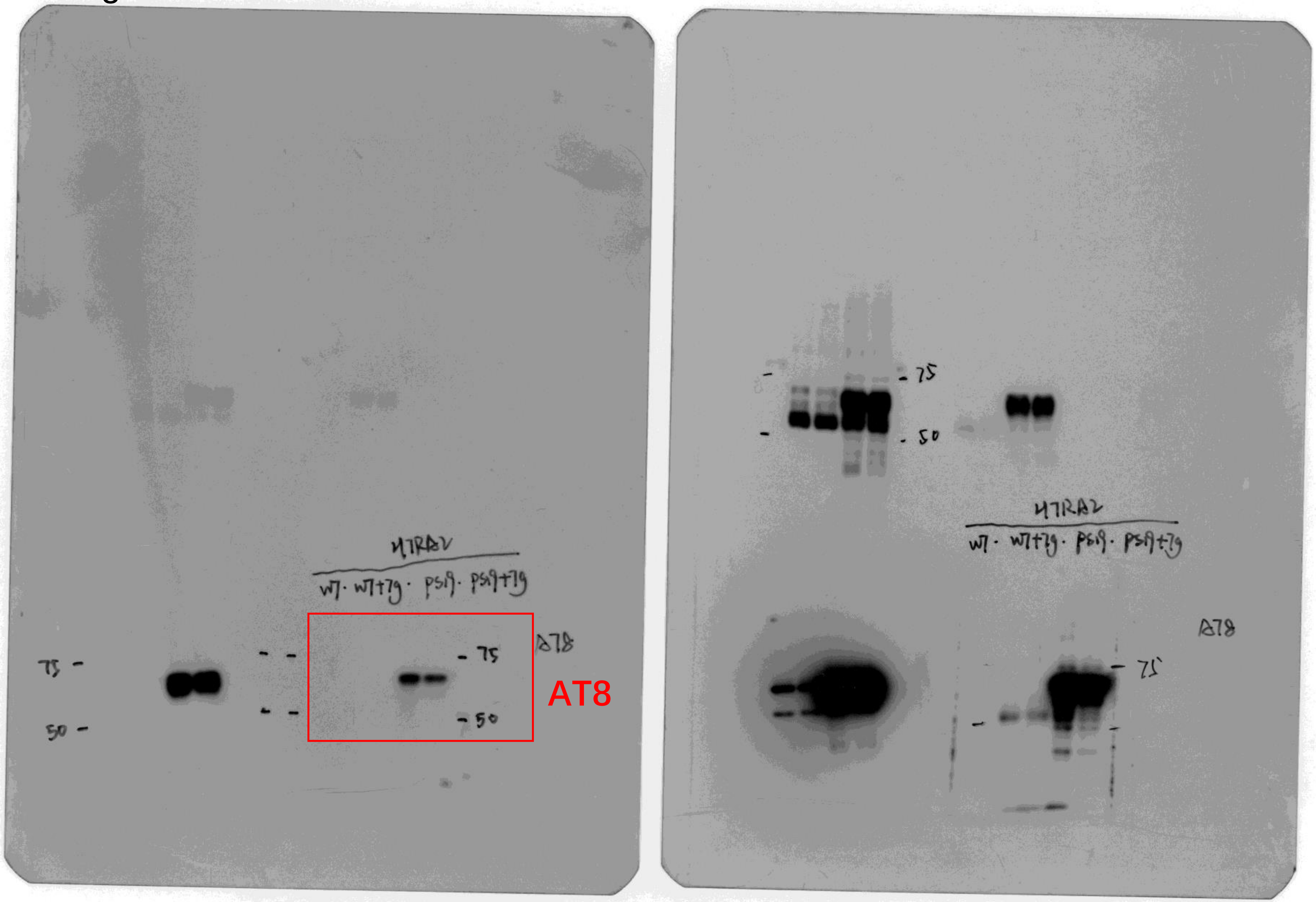

Full unedited gel for Figure 5F

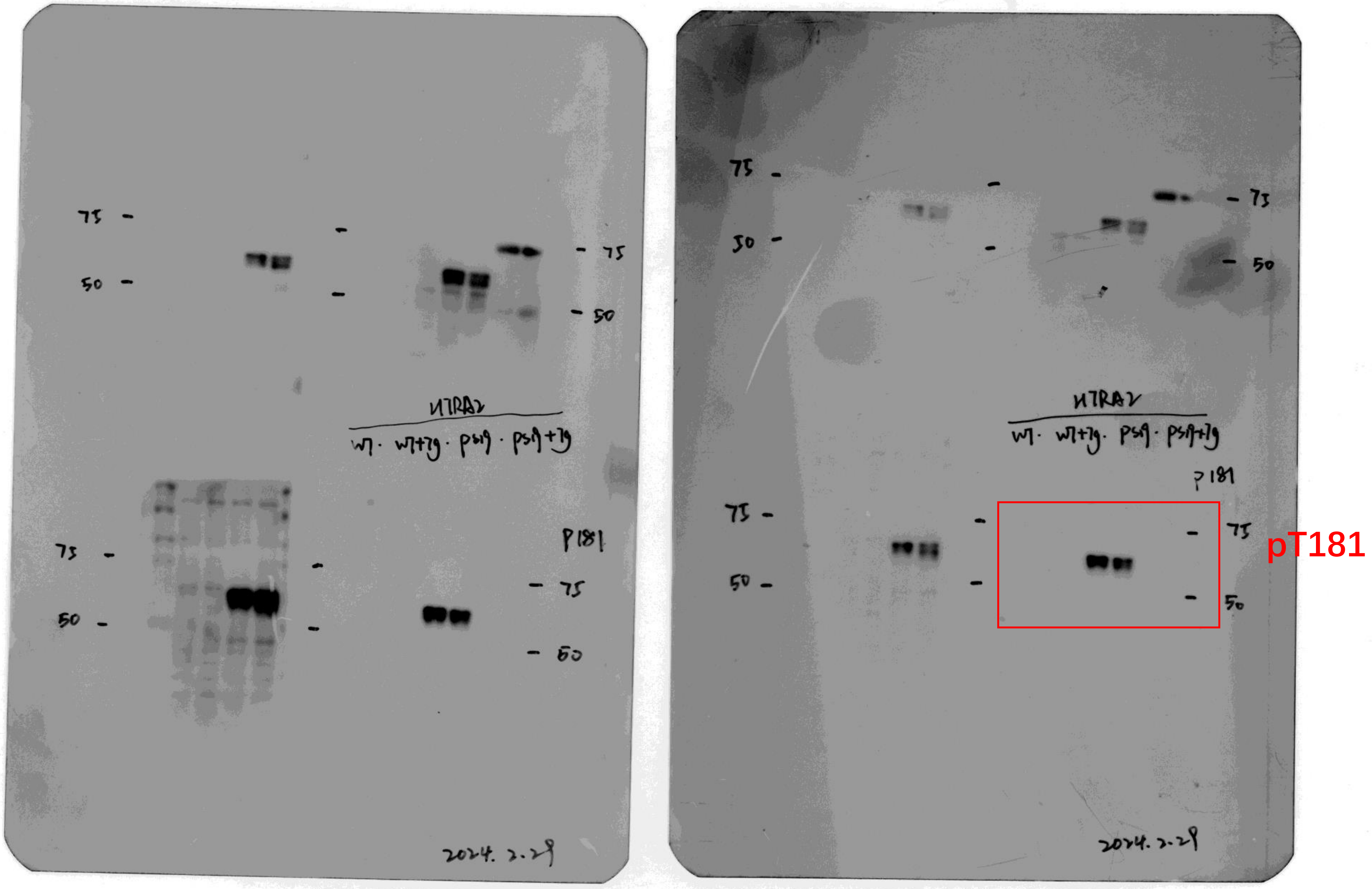

Full unedited gel for Figure 5F

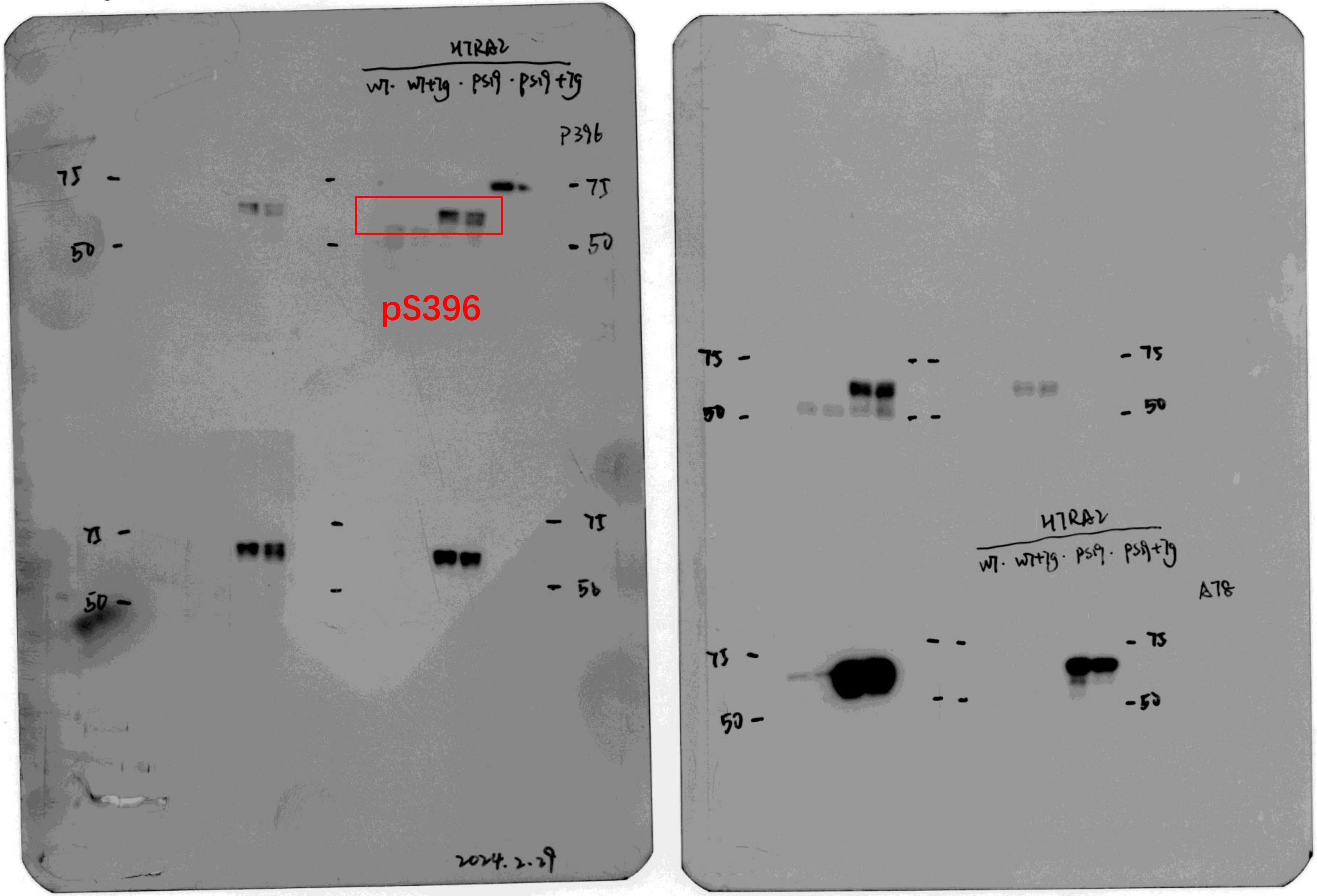

Full unedited gel for Figure 5F

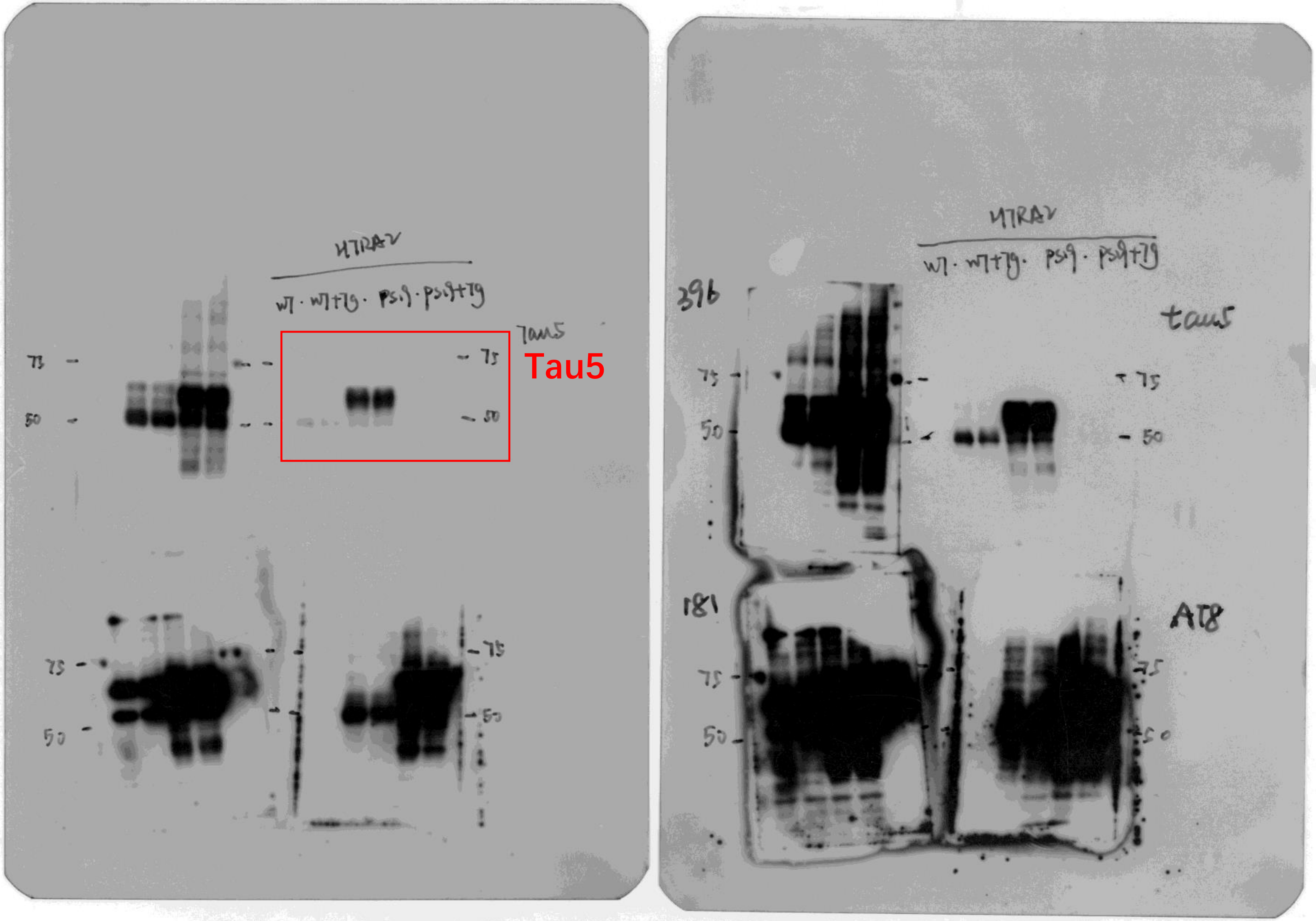

Full unedited gel for Figure 5F

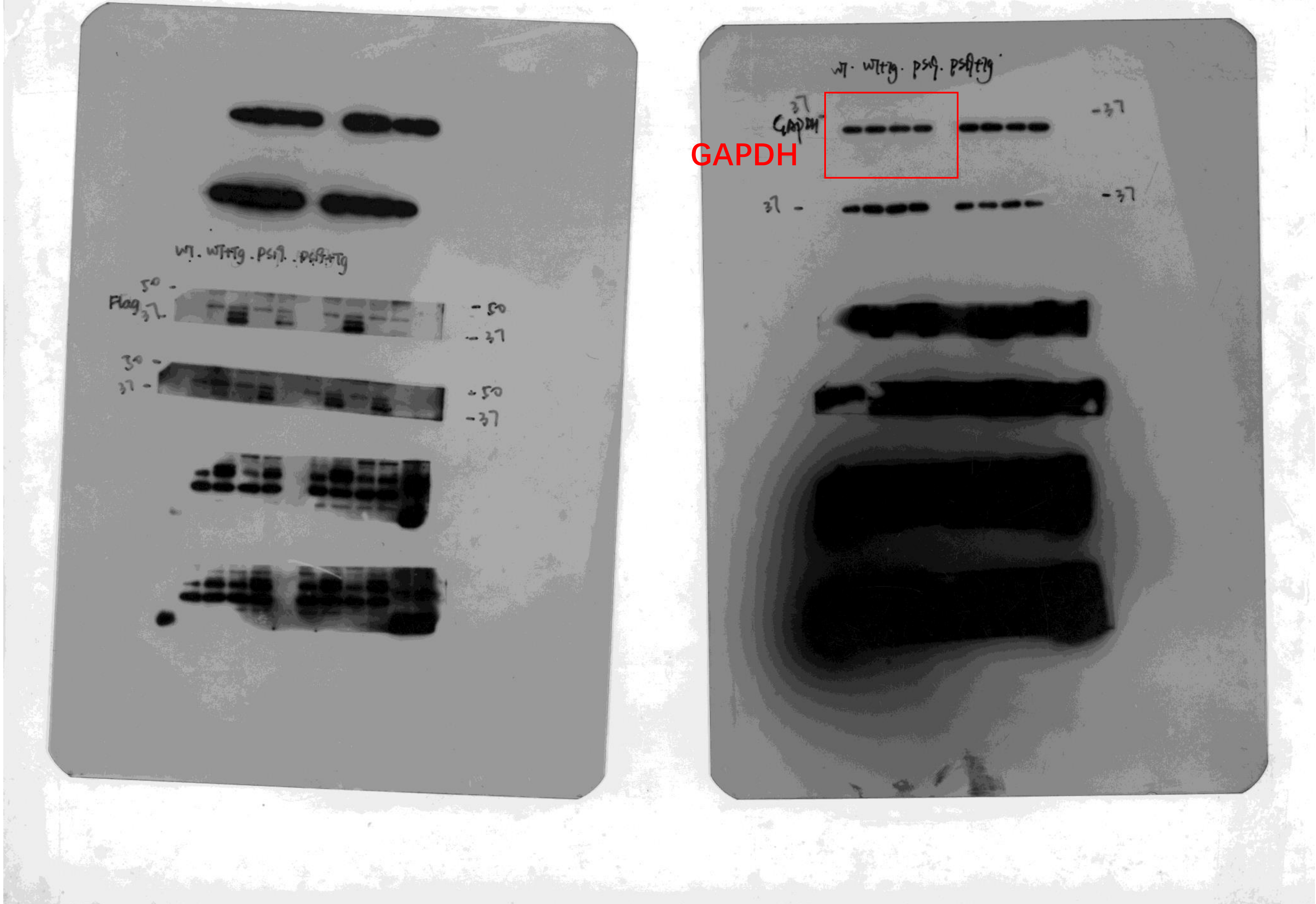

Full unedited gel for Figure 5G

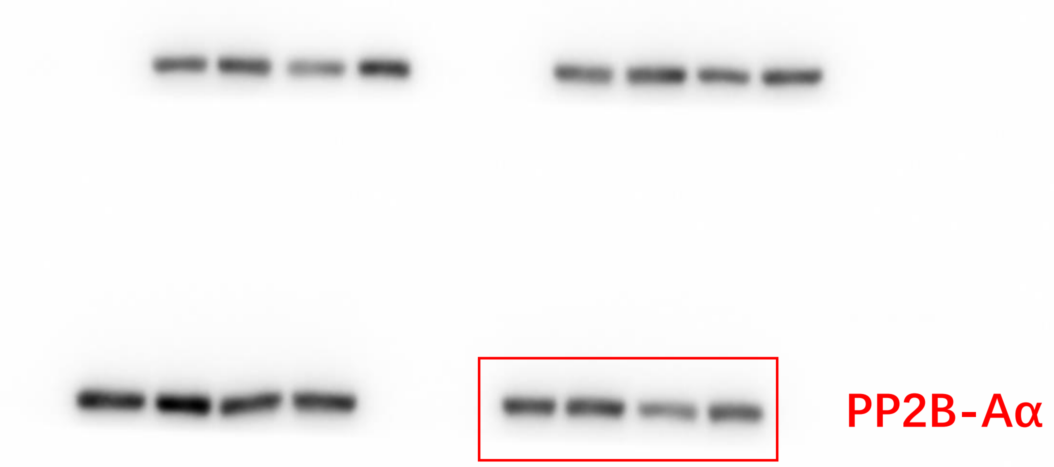

The membrane was imaged with Azure Biosystems 300

Full unedited gel for Figure 5G

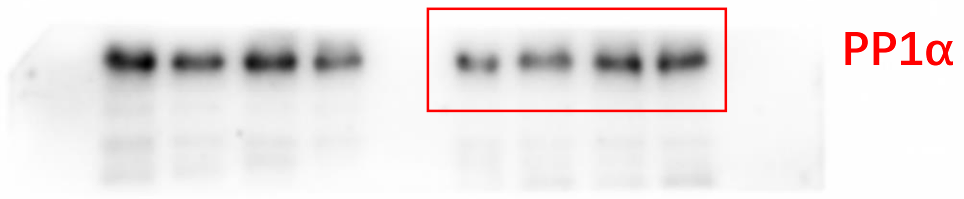

The membrane was imaged with Azure Biosystems 300

Full unedited gel for Figure 5G

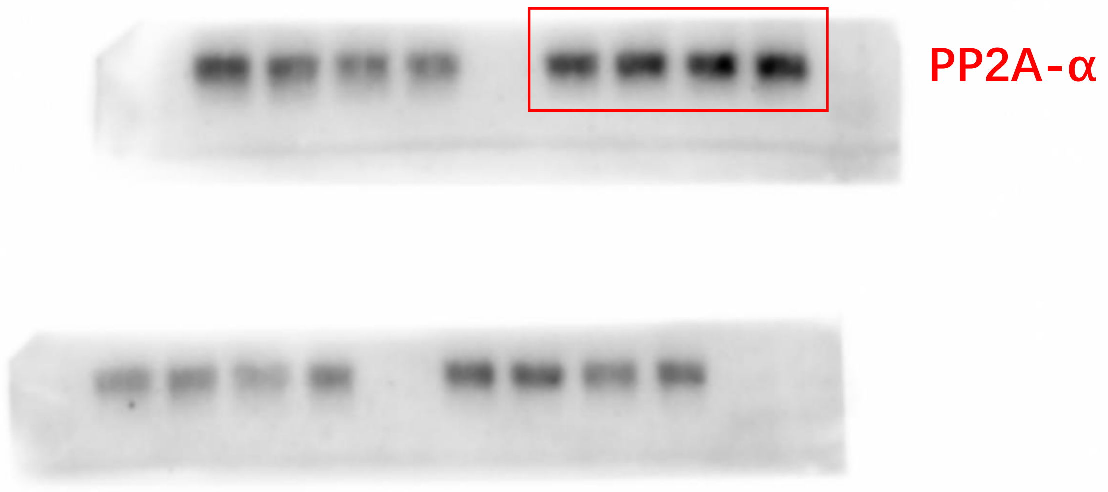

The membrane was imaged with Azure Biosystems 300

Full unedited gel for Figure 5G

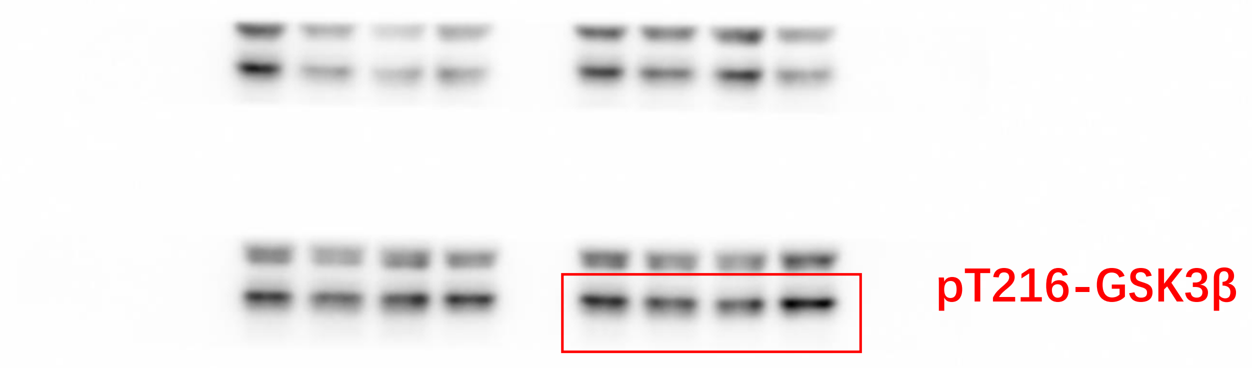

The membrane was imaged with Azure Biosystems 300

Full unedited gel for Figure 5G

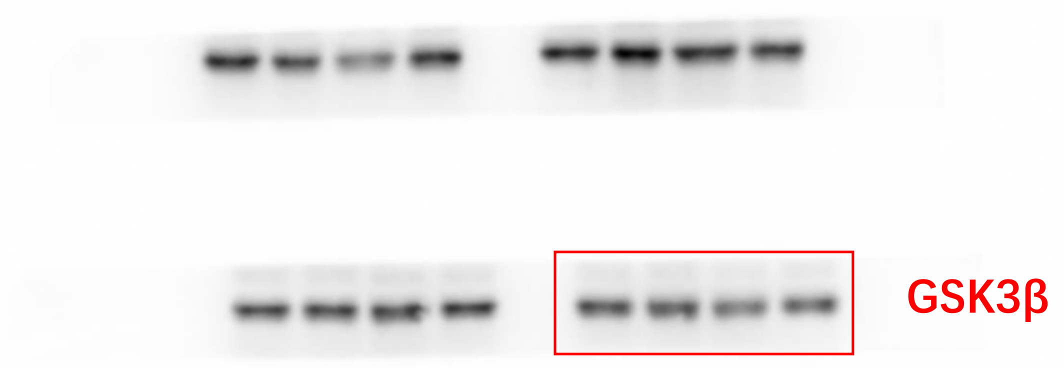

The membrane was imaged with Azure Biosystems 300

Full unedited gel for Figure 5G

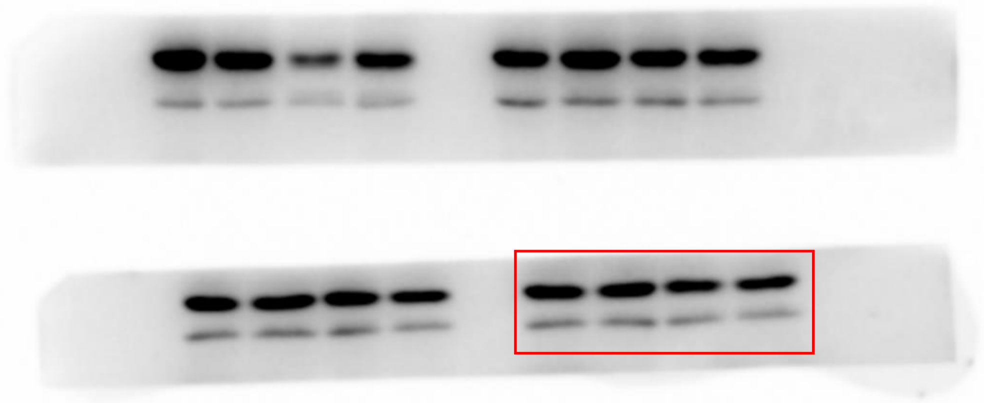

The membrane was imaged with Azure Biosystems 300

Full unedited gel for Figure 5G

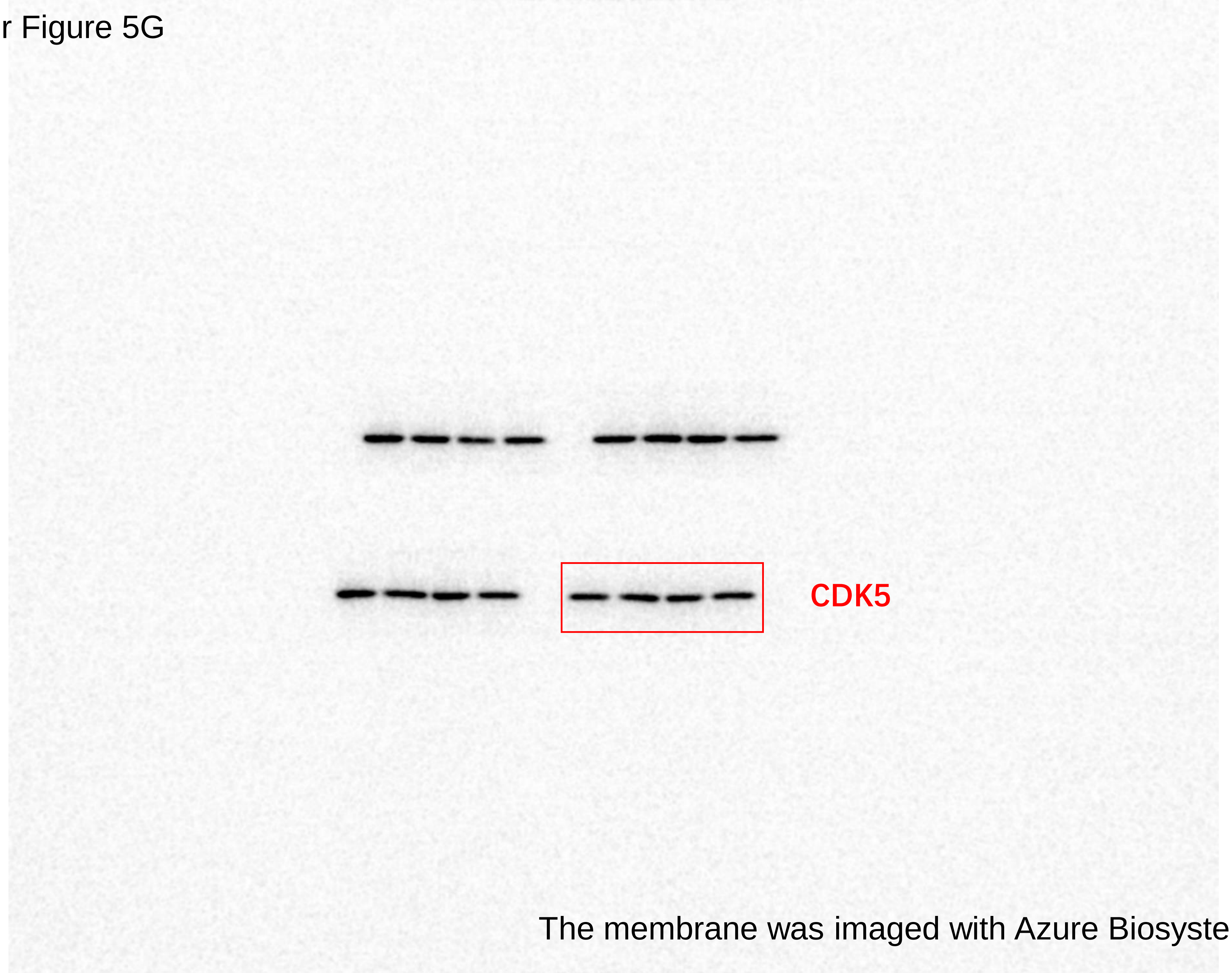

The membrane was imaged with Azure Biosystems 300

Full unedited gel for Figure 5G

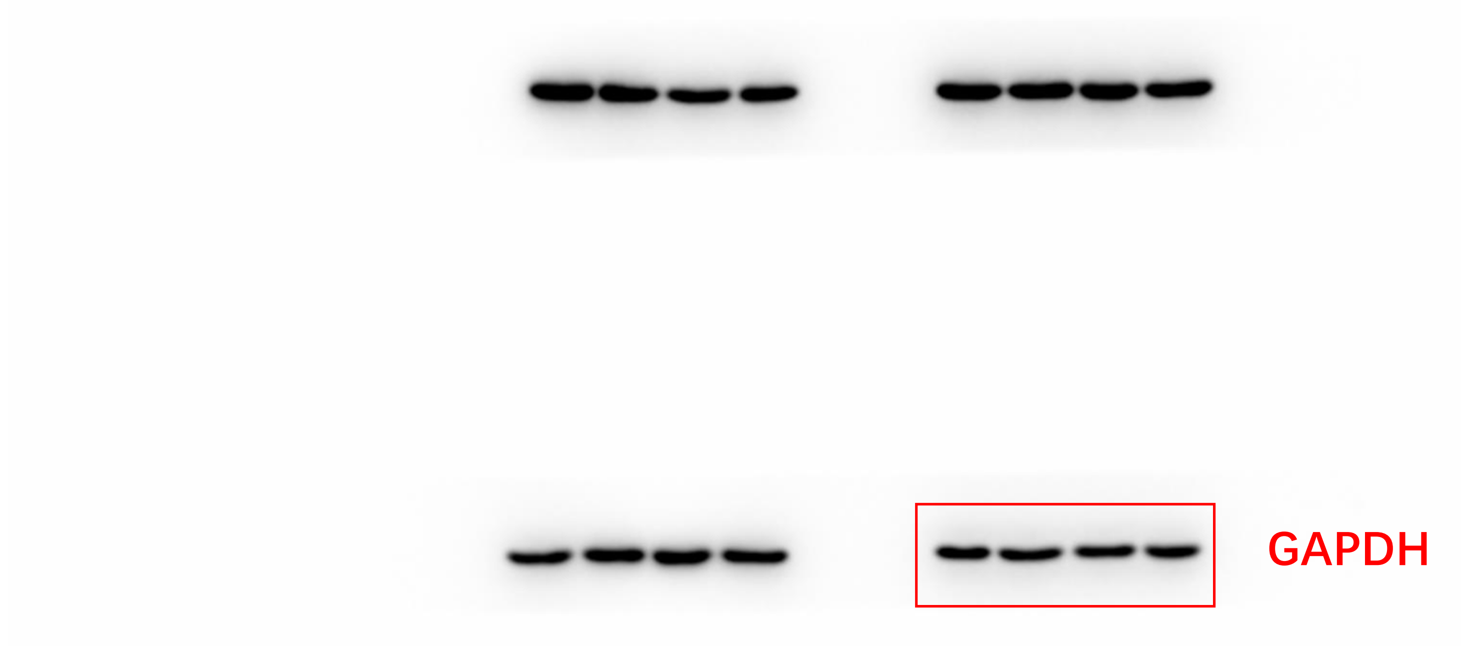

The membrane was imaged with Azure Biosystems 300

Full unedited gel for Supplemental Figure 2A

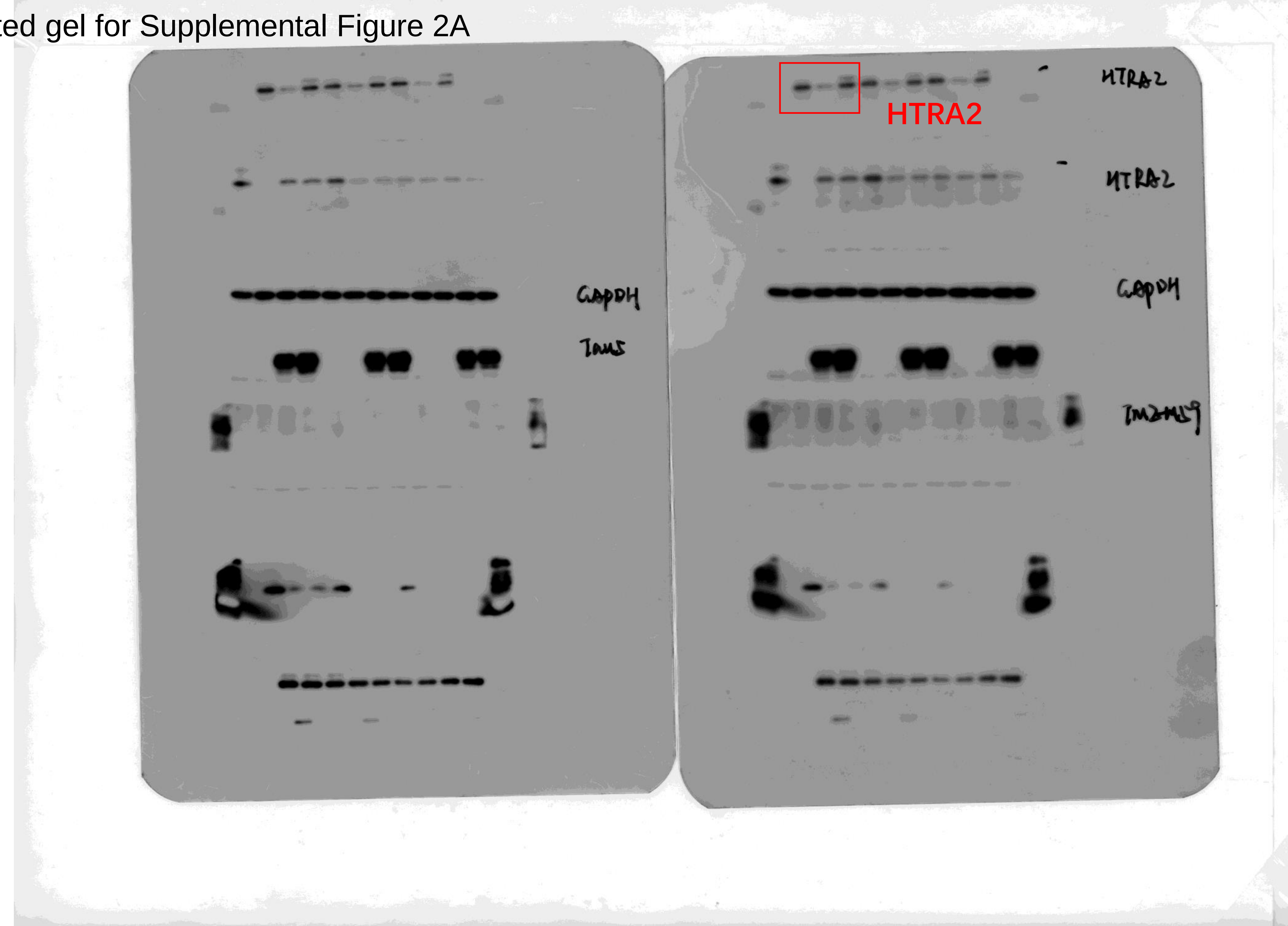

Full unedited gel for Supplemental Figure 2A

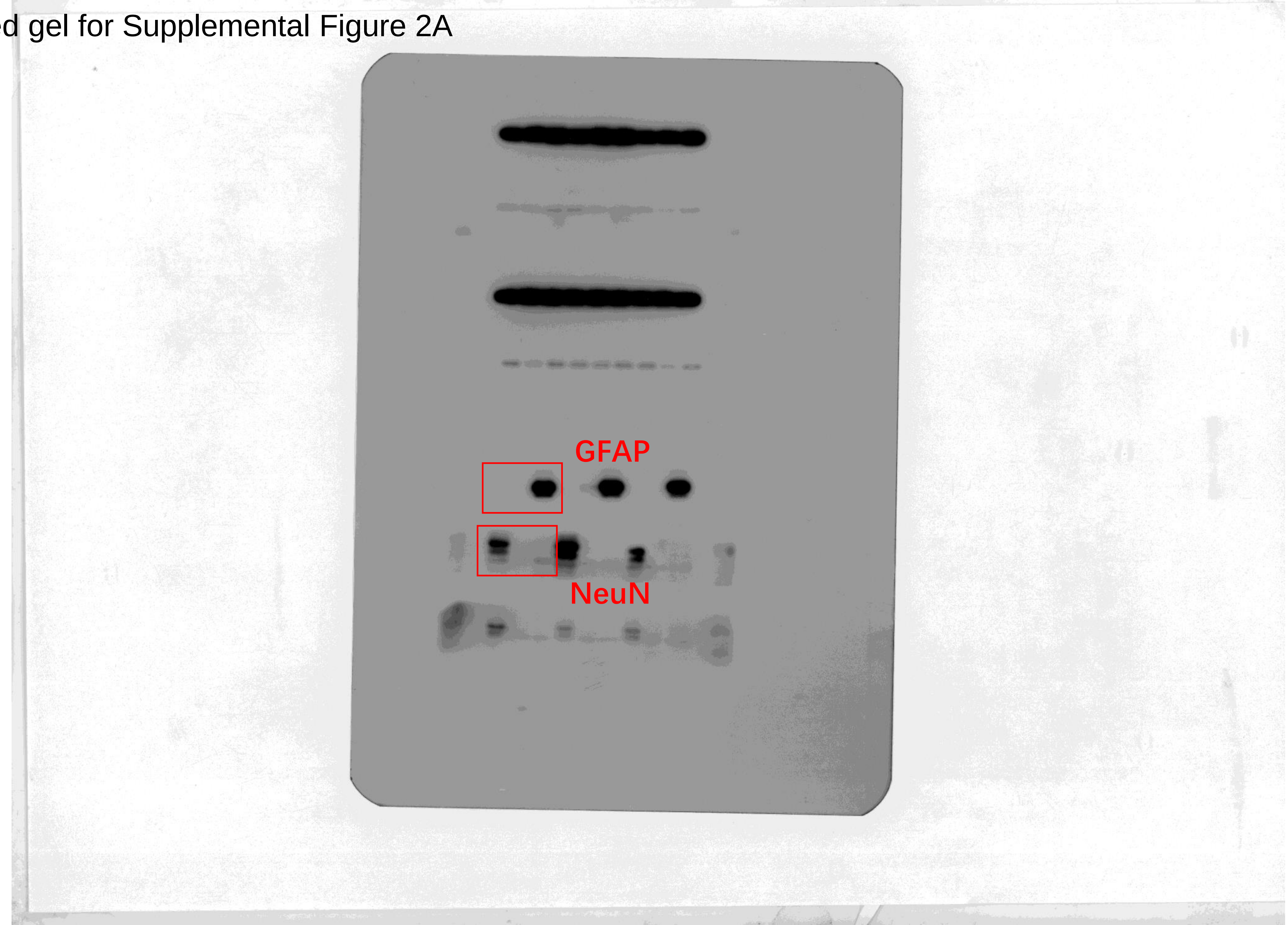

Full unedited gel for Supplemental Figure 2A

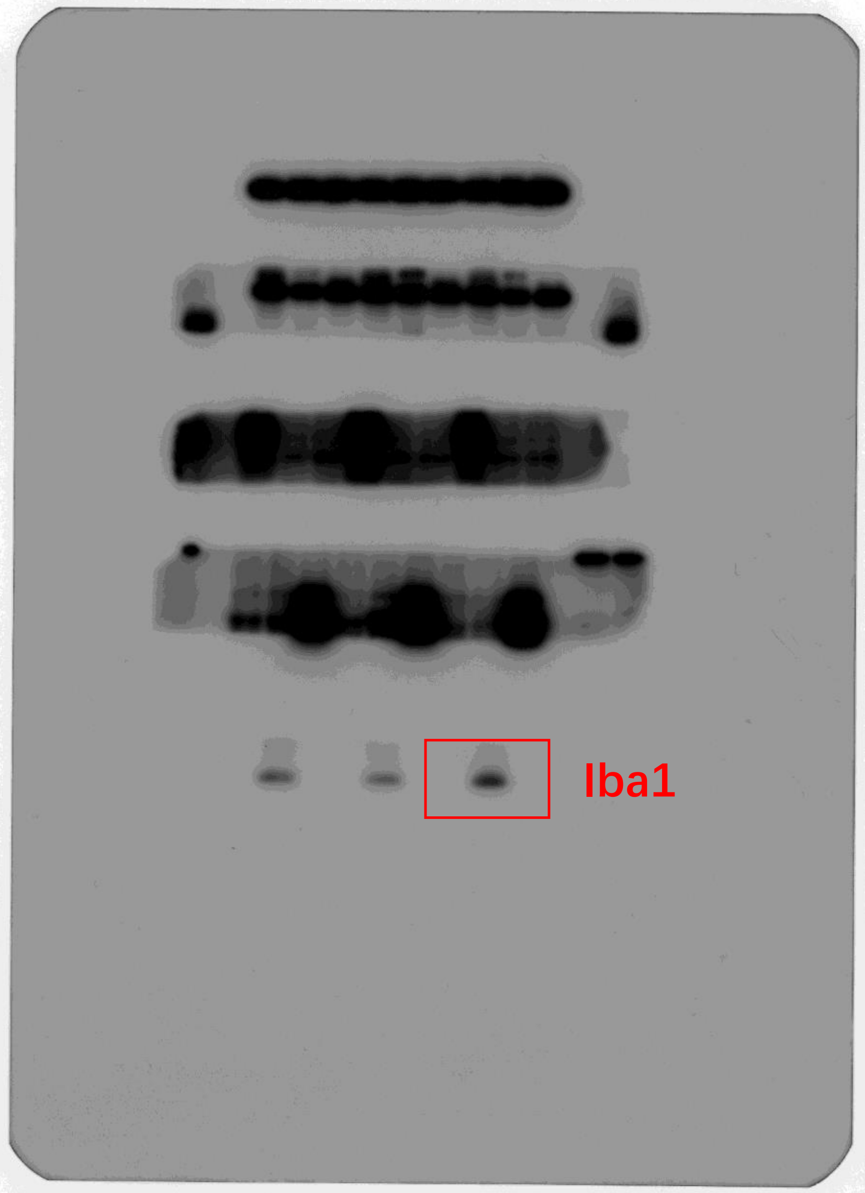

Full unedited gel for Supplemental Figure 2A

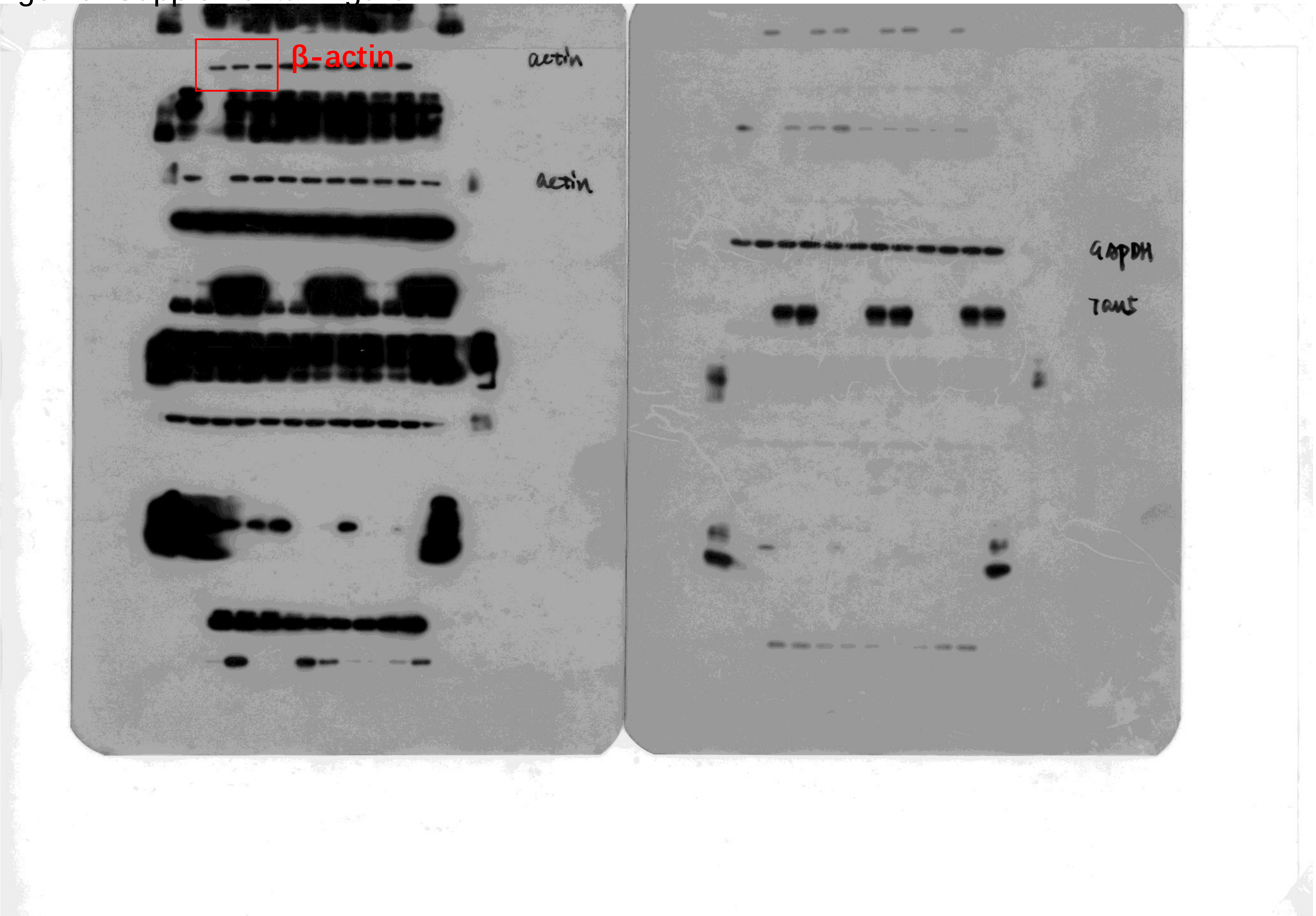

Full unedited gel for Supplemental Figure 3A

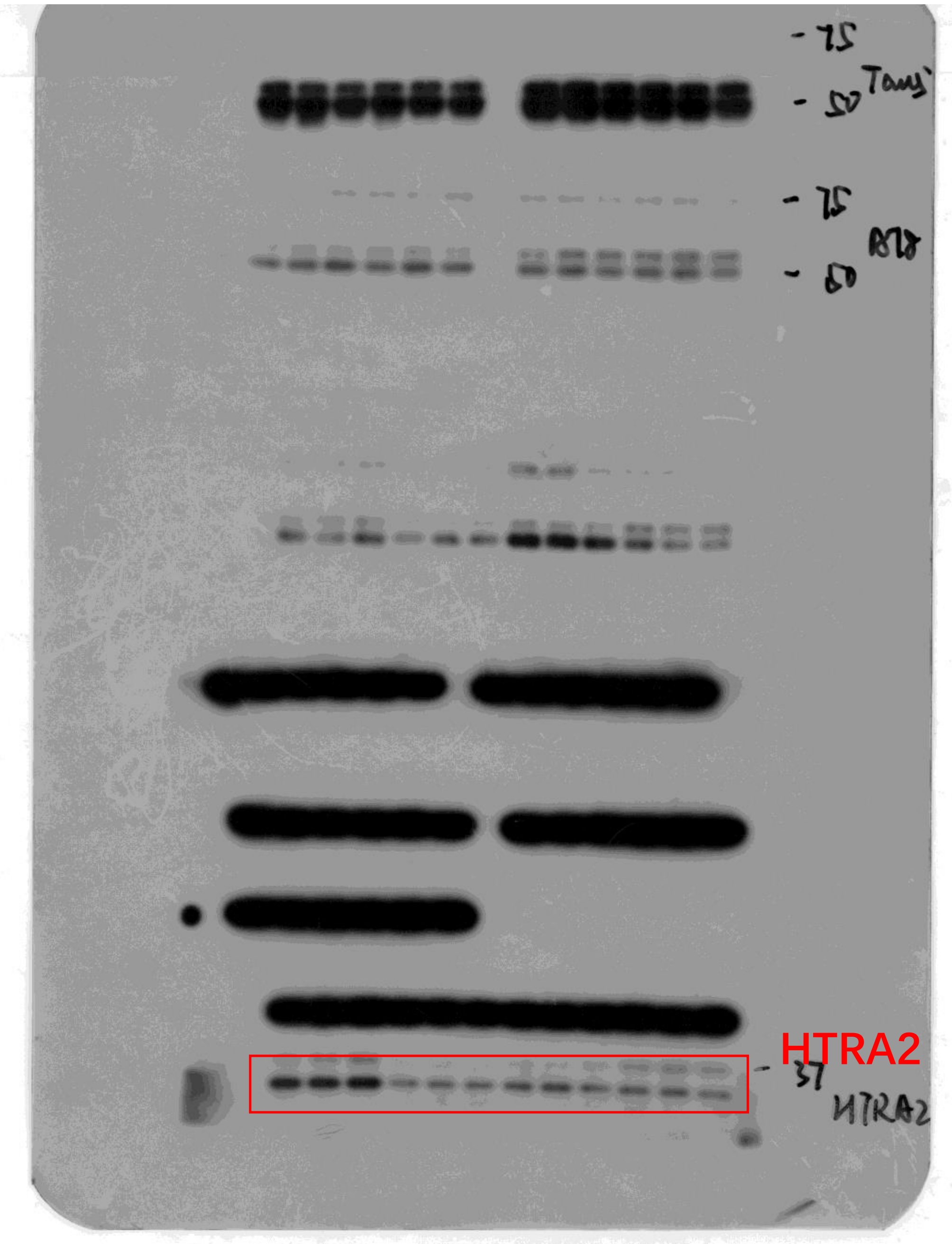

Full unedited gel for Supplemental Figure 3A

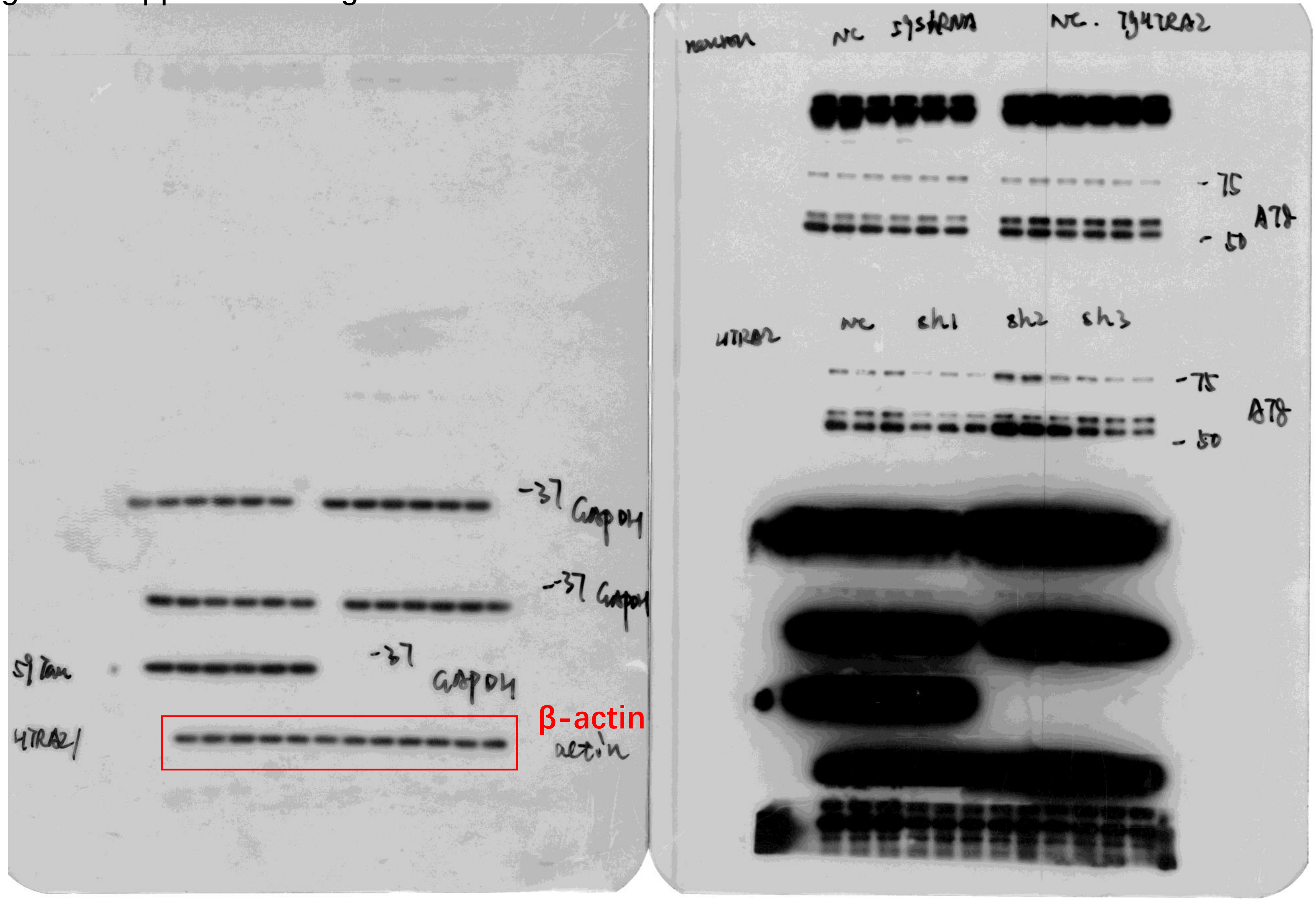

Full unedited gel for Supplemental Figure 4J

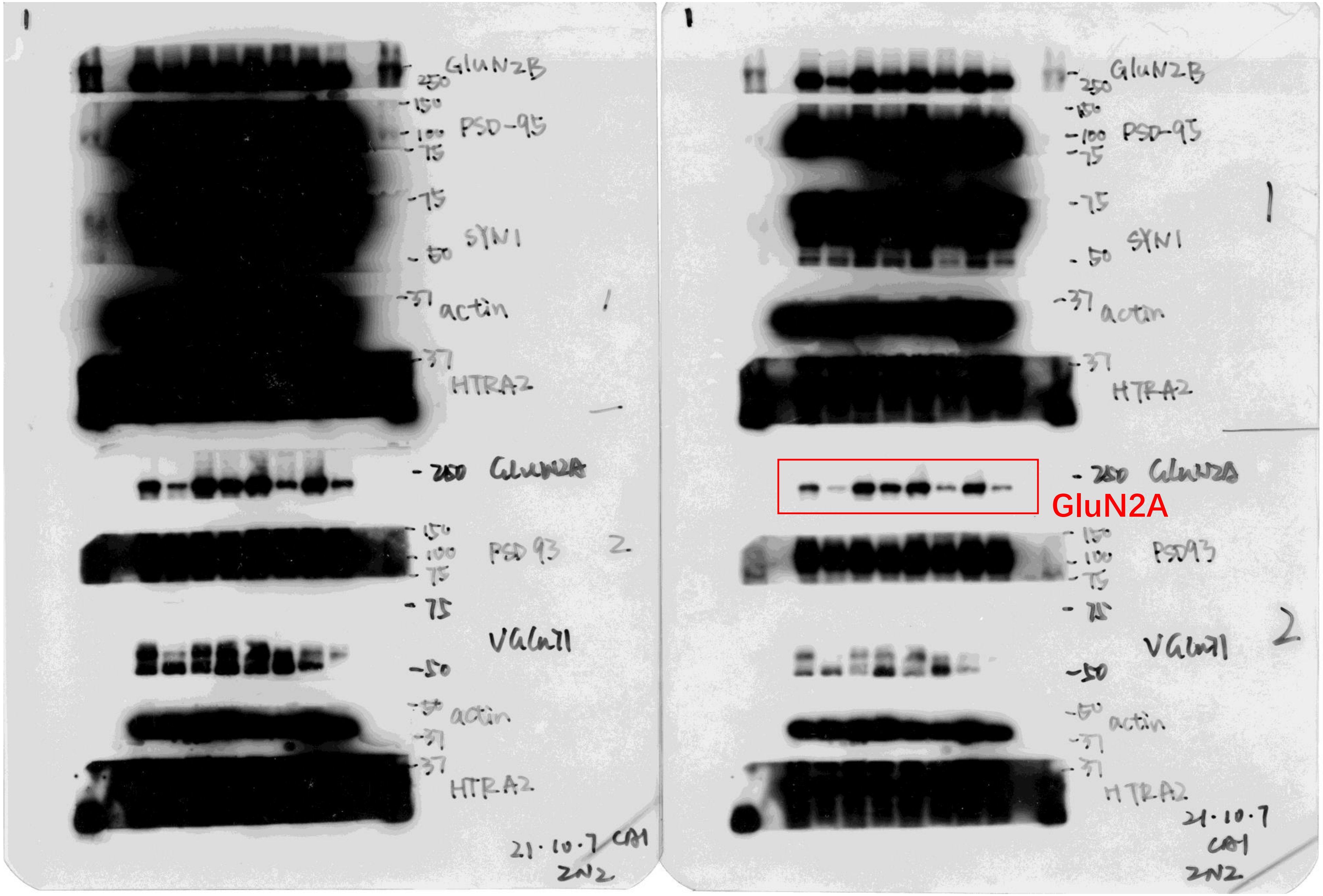

Full unedited gel for Supplemental Figure 4J

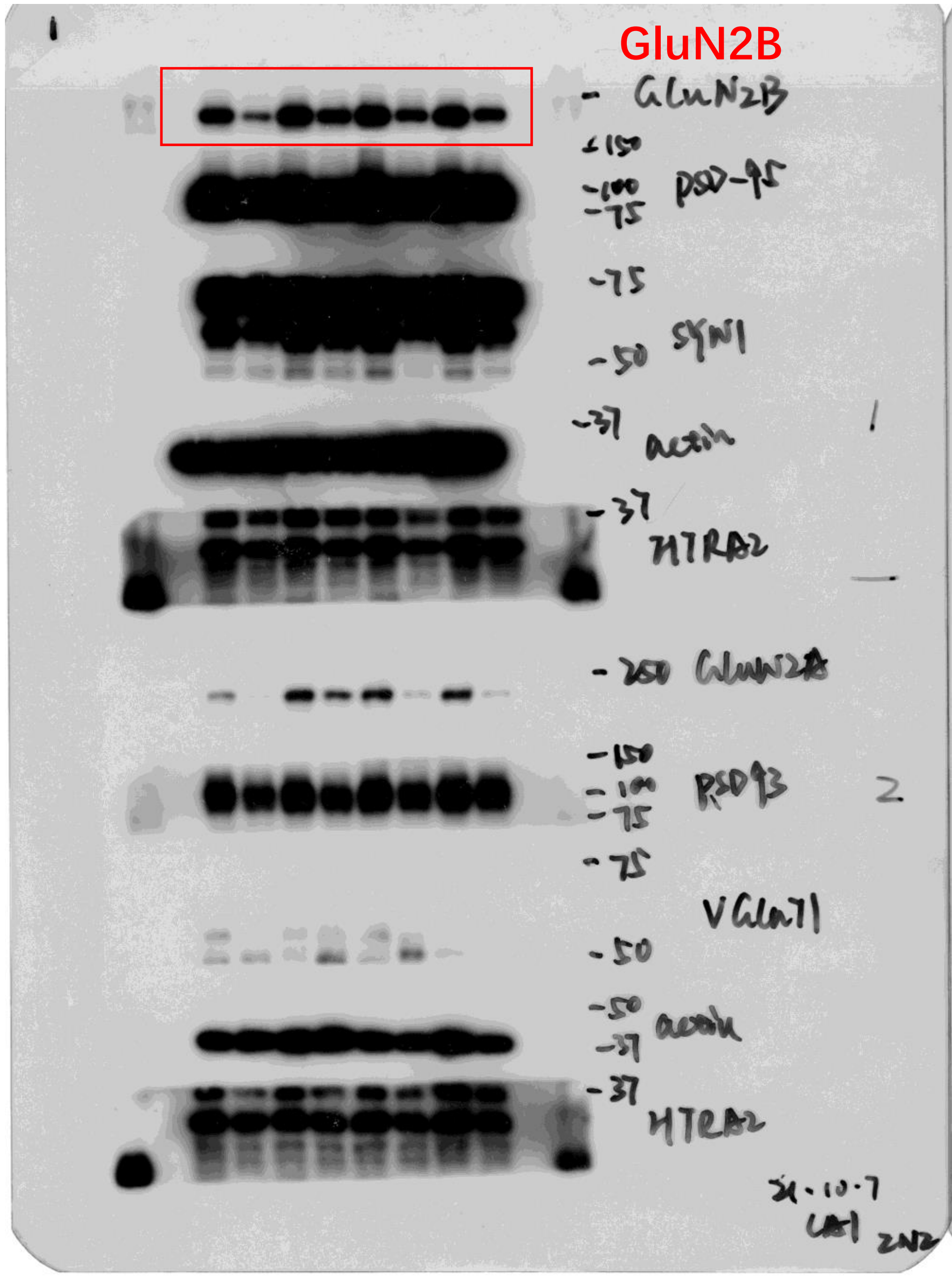

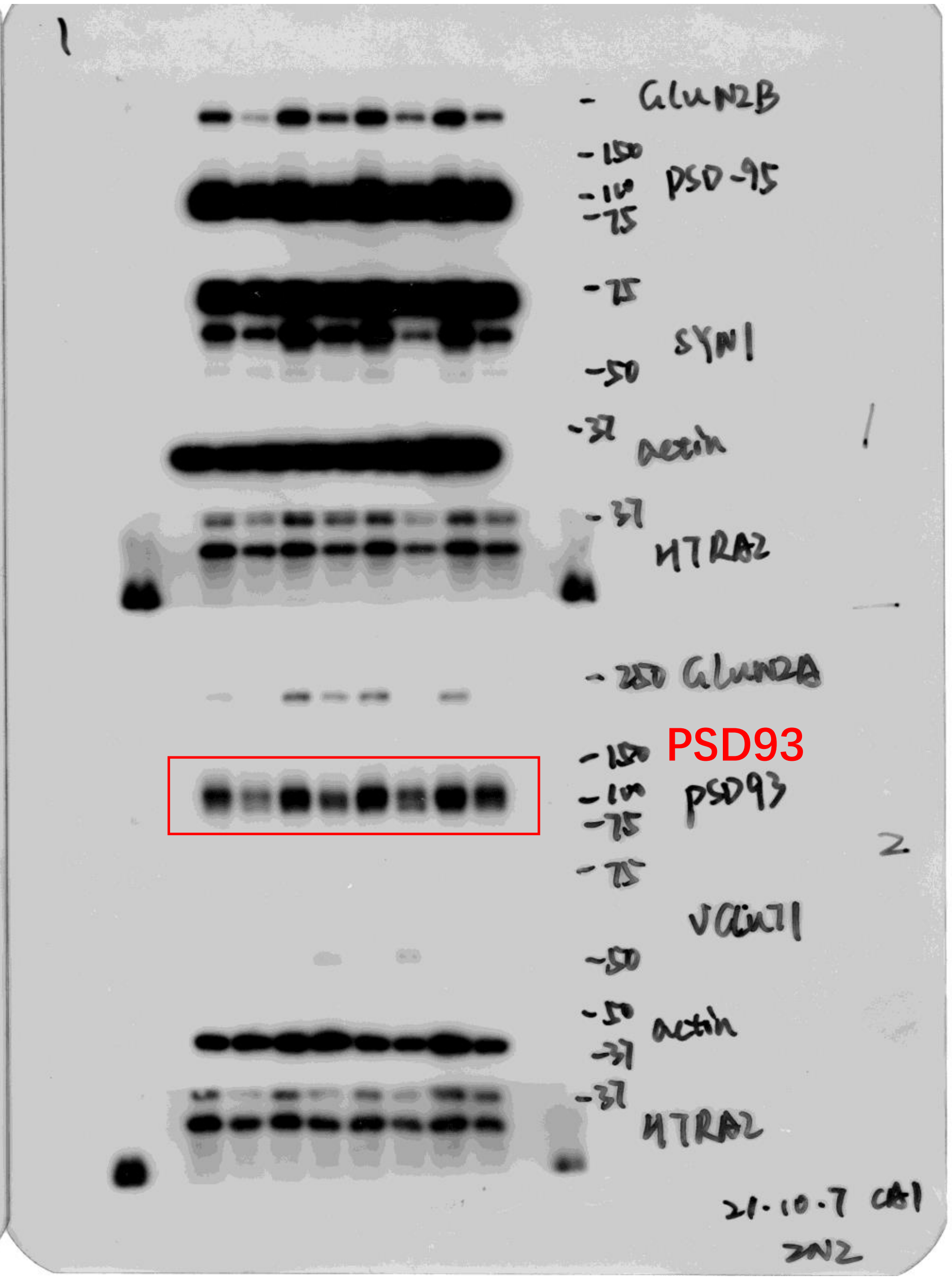

Full unedited gel for Supplemental Figure 4J

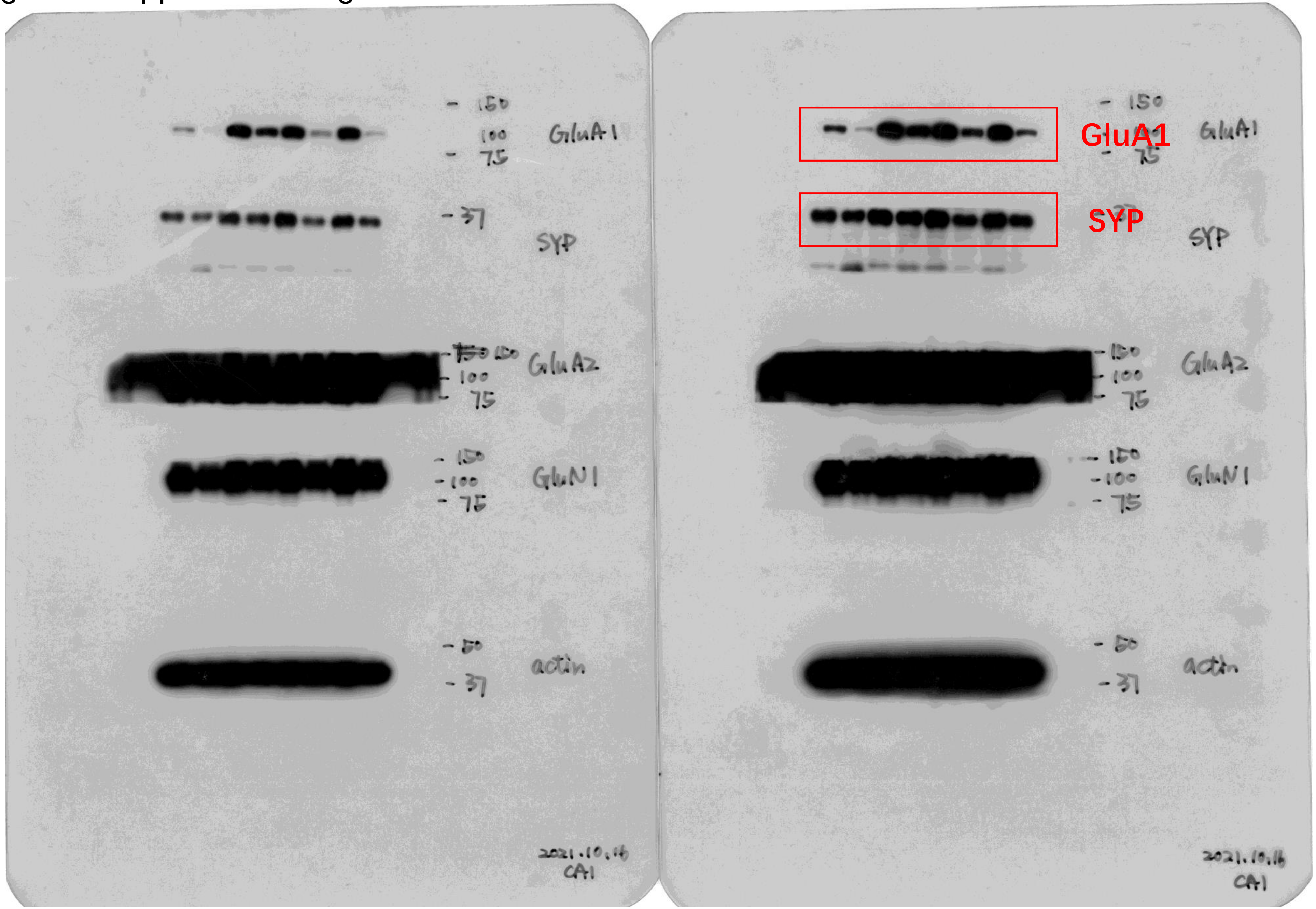

Full unedited gel for Supplemental Figure 4J

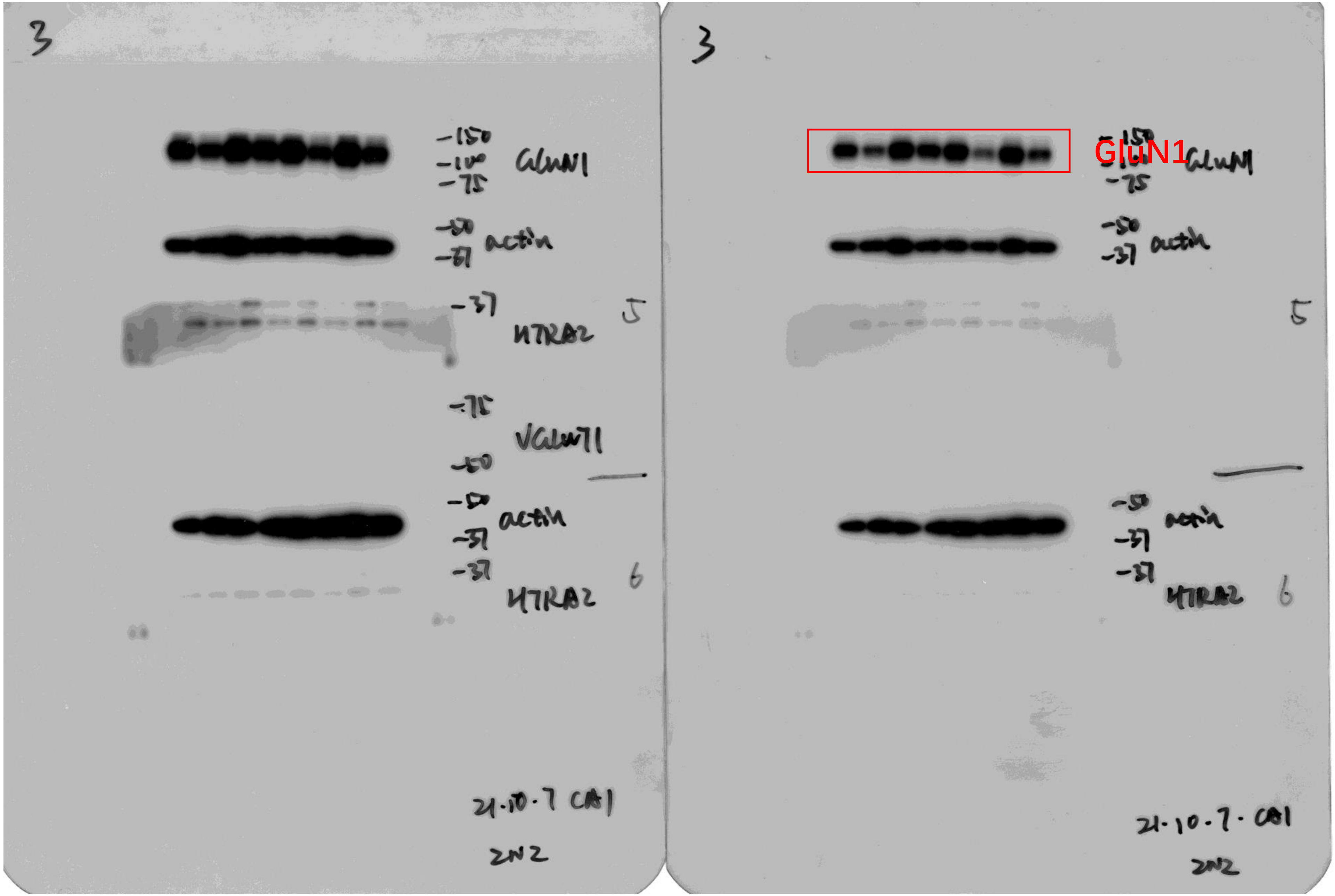

Full unedited gel for Supplemental Figure 4J

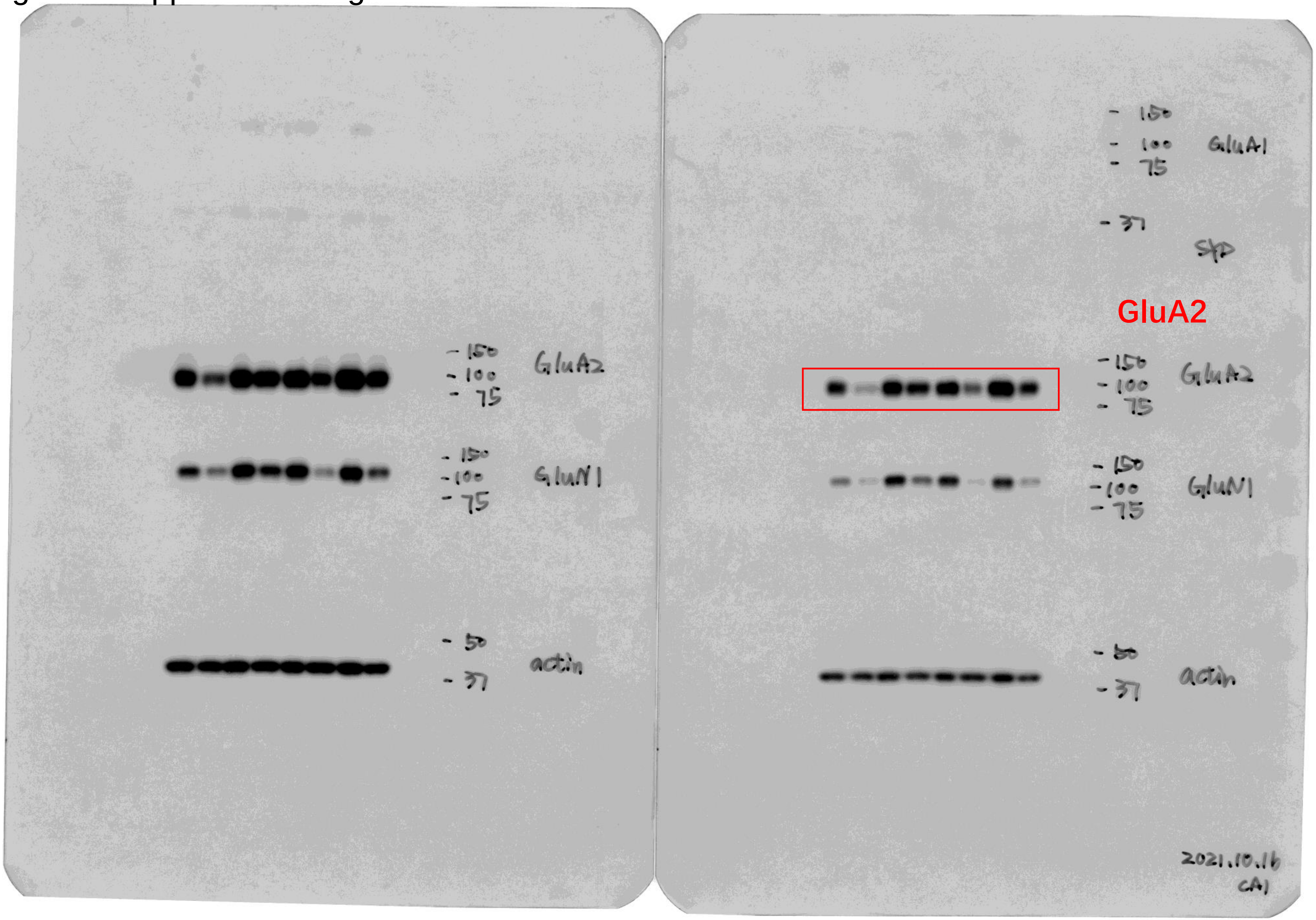

Full unedited gel for Supplemental Figure 4J

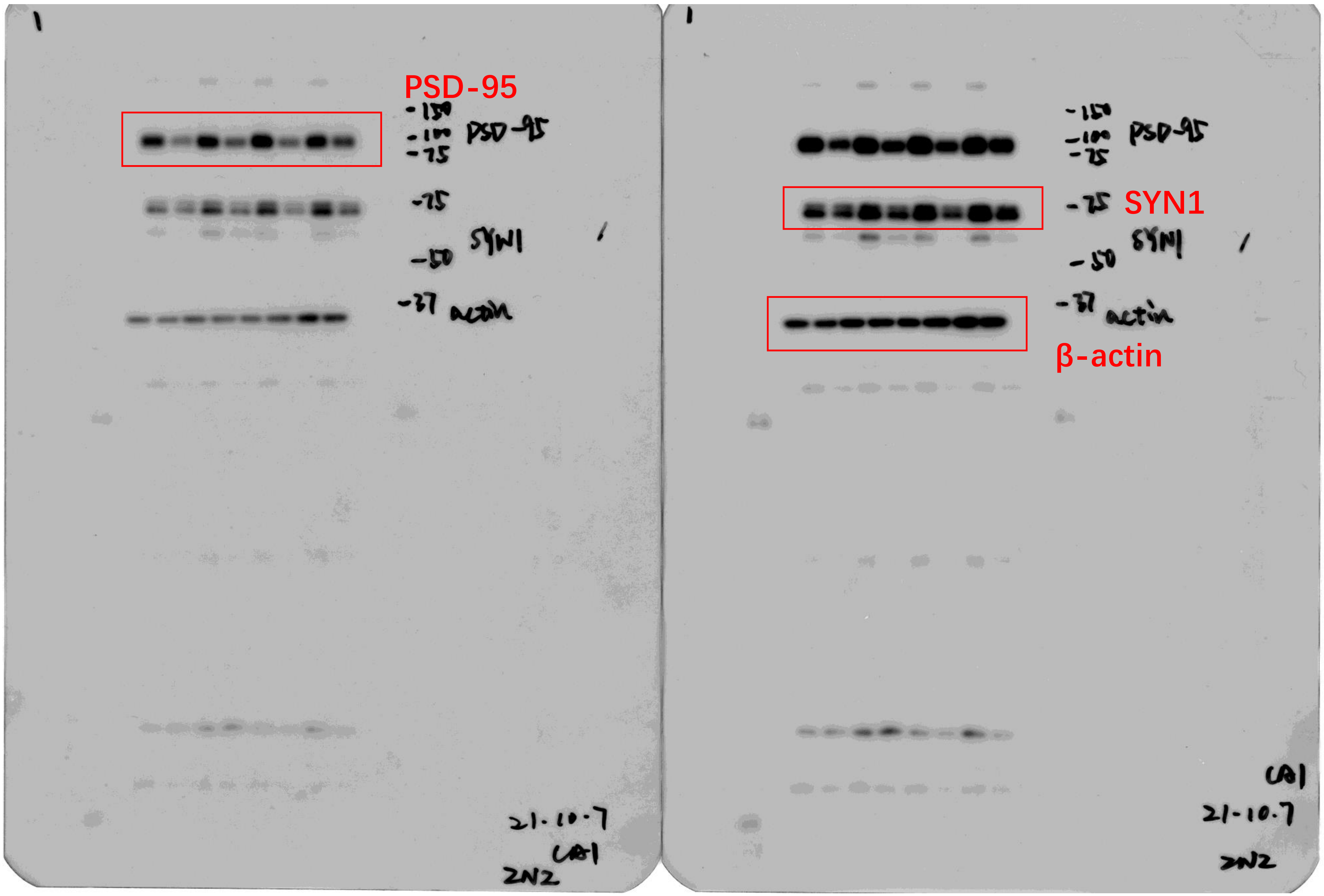

Full unedited gel for Supplemental Figure 4J

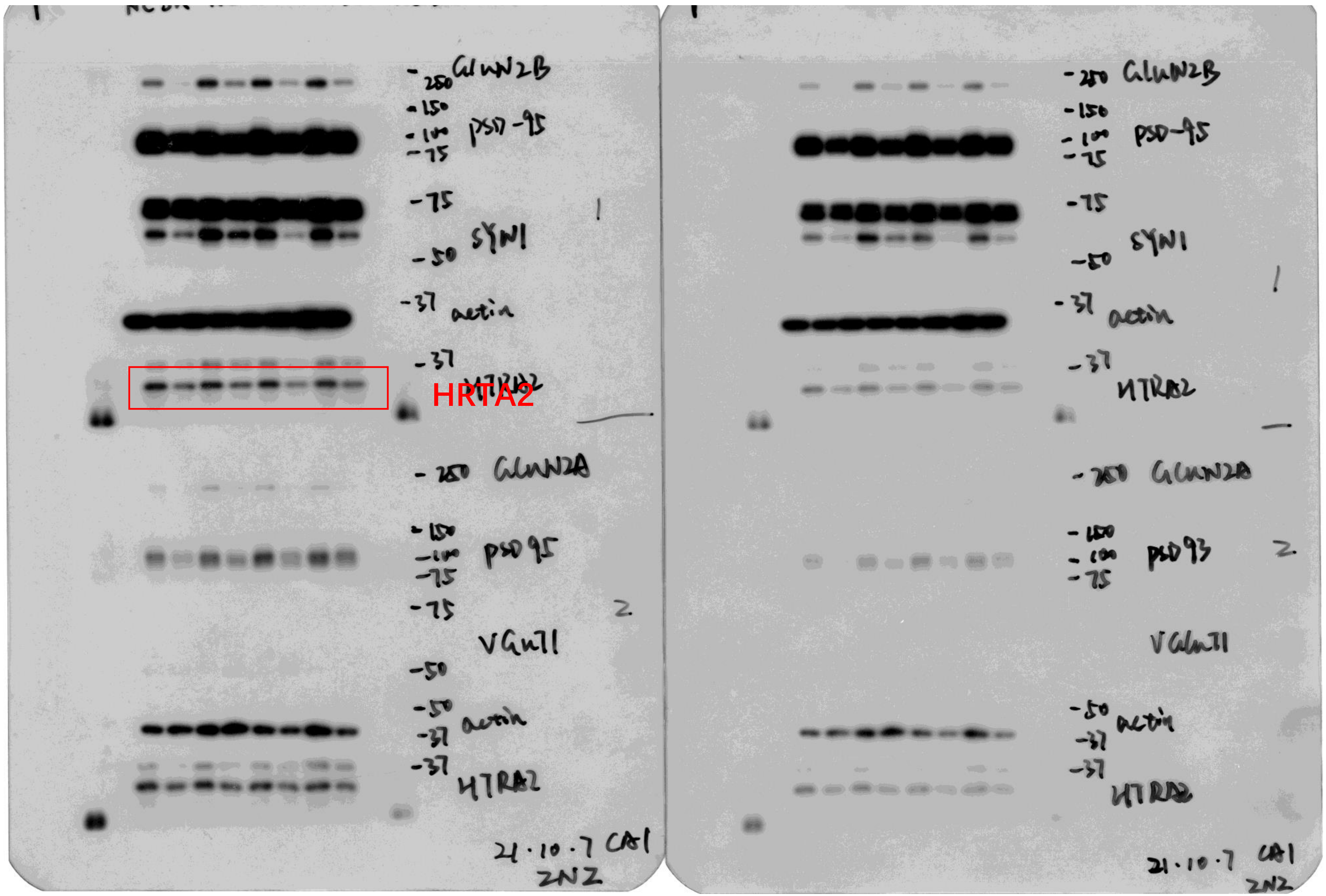

Full unedited gel for Supplemental Figure 6

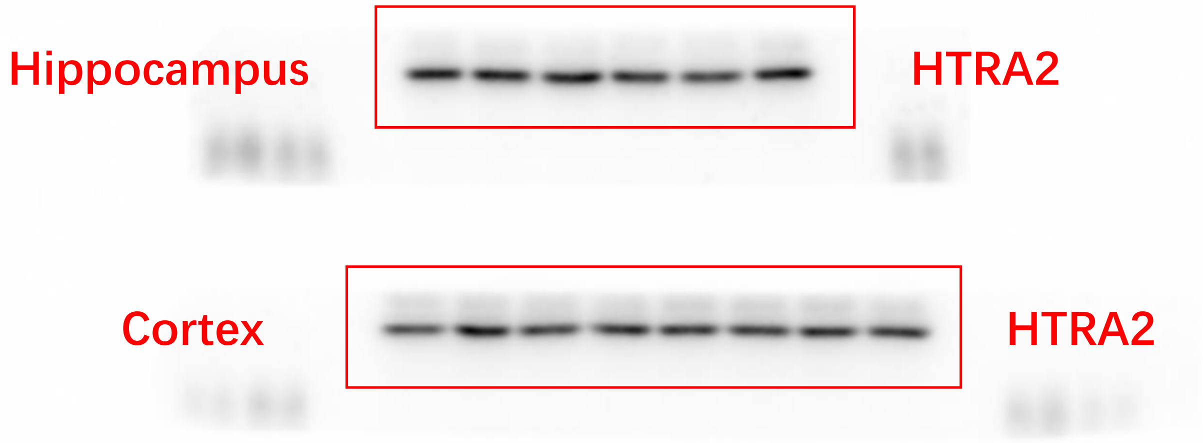

The membrane was imaged with Azure Biosystems 300

Full unedited gel for Supplemental Figure 6

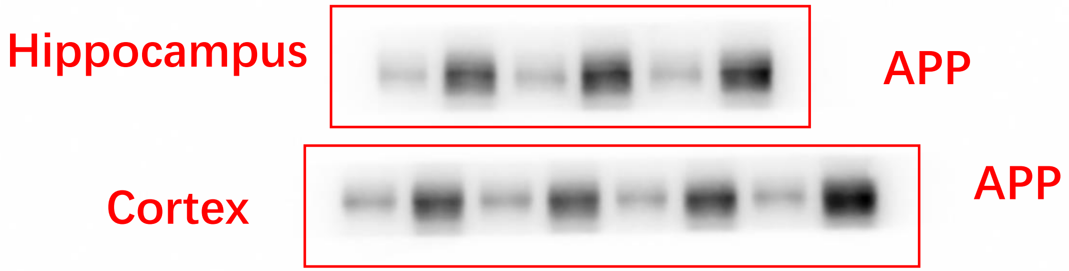

The membrane was imaged with Azure Biosystems 300

Full unedited gel for Supplemental Figure 6

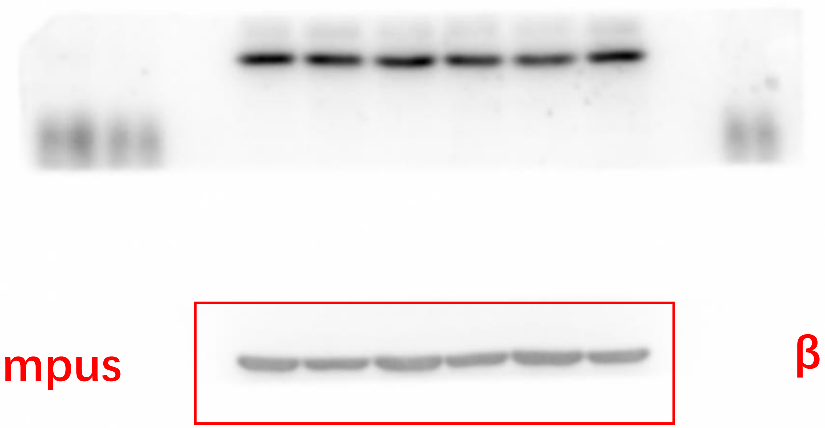

Hippocampus

β-actin

The membrane was imaged with Azure Biosystems 300

Full unedited gel for Supplemental Figure 6

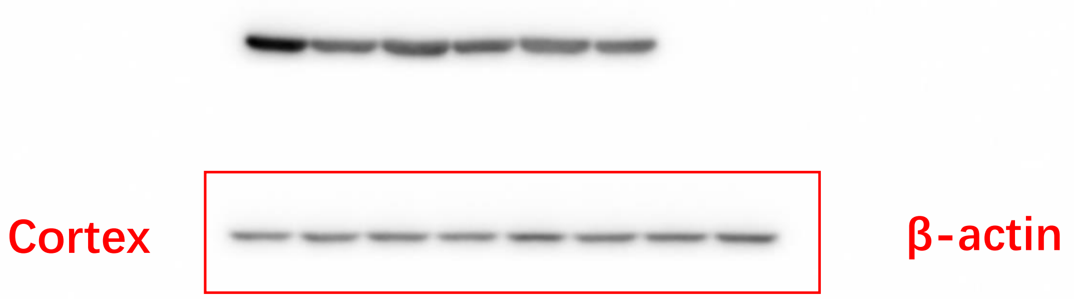

The membrane was imaged with Azure Biosystems 300
